# Genetically predicted androgenic profiles and adverse cardiac markers: a sex‐specific Mendelian randomization study

**DOI:** 10.1002/ehf2.14527

**Published:** 2023-09-22

**Authors:** Jun Yu Chen, Maddalena Ardissino, Rohin K. Reddy, Amy Marie Mason, Zahra Raisi‐Estabragh, Emanuele Di Angelantonio, Stephen Burgess, Fu Siong Ng

**Affiliations:** ^1^ National Heart and Lung Institute Imperial College London London UK; ^2^ British Heart Foundation Cardiovascular Epidemiology Unit, Department of Public Health and Primary Care University of Cambridge Cambridge UK; ^3^ Heart and Lung Research Institute University of Cambridge Cambridge UK; ^4^ William Harvey Research Institute, NIHR Barts Biomedical Research Centre Queen Mary University of London London UK; ^5^ Barts Heart Centre, St Bartholomew's Hospital Barts Health NHS Trust London UK; ^6^ National Institute for Health and Care Research Blood and Transplant Research Unit in Donor Health and Behaviour University of Cambridge Cambridge UK; ^7^ British Heart Foundation Centre of Research Excellence University of Cambridge Cambridge UK; ^8^ Health Data Research UK Cambridge Wellcome Genome Campus and University of Cambridge Cambridge UK; ^9^ Health Data Science Research Centre Human Technopole Milan Italy; ^10^ Medical Research Council Biostatistics Unit University of Cambridge Cambridge UK

**Keywords:** SHBG, Testosterone, Sex hormones, Heart failure, CMR, Mendelian randomization

## Abstract

**Aims:**

Observational evidence suggests associations between sex hormone levels and heart failure (HF). We used sex‐specific genetic variants associated with androgenic sex hormone profiles to investigate the causal relevance of androgenic sex hormone profiles on cardiac structure and function and HF using Mendelian randomization (MR).

**Methods and results:**

Sex‐specific uncorrelated genome‐wide significant (*P* < 5 × 10^−8^) variants predicting sex hormone‐binding globulin (SHBG), total testosterone, and bioavailable testosterone were extracted from summary statistics of genome‐wide association study (GWAS) on 425 097 participants in the UK Biobank. Sex‐specific gene–outcome association estimates were computed for left ventricular ejection fraction (LVEF), left ventricular end‐diastolic and end‐systolic volumes (LVEDV and LVESV, respectively), left ventricular stroke volume (LVSV), cardiac index, and cardiac output in 11 528 female and 14 356 male UK Biobank Imaging Study participants and for incident or prevalent HF in an external cohort of 47 309 cases and 930 014 controls. Inverse‐variance weighted MR was the primary analysis method. In females, higher genetically predicted bioavailable testosterone was associated with lower LVEDV [*β* per nmol/L = −0.11 (−0.19 to −0.03), *P* = 0.006], lower LVESV [*β* = −0.09 (−0.17 to −0.01), *P* = 0.022], lower LVSV [*β* = −0.11 (−0.18 to −0.03), *P* = 0.005], lower cardiac output [*β* = −0.08 (−0.16 to 0.00), *P* = 0.046], and lower cardiac index [*β* = −0.08 (−0.16 to −0.01), *P* = 0.034] and a higher risk of HF [odds ratio 1.10 (1.01–1.19), *P* = 0.026] on external validation analysis in larger scale, sex‐adjusted GWAS data. Higher genetically predicted SHBG was associated with higher LVEDV [*β* per nmol/L = 0.17 (0.08–0.25), *P* = 2 × 10^−4^], higher LVESV [*β* = 0.13 (0.05–0.22), *P* = 0.003], and higher LVSV [*β* = 0.18 (0.08–0.28), *P* = 2 × 10^−4^]. In males, higher genetically predicted total and bioavailable testosterone was associated with lower LVESV [*β* = −0.07 (−0.12 to −0.02), *P* = 0.007] and LVEF [*β* = −0.11 (−0.18 to −0.04), *P* = 0.003], respectively.

**Conclusions:**

This study supports a causal effect of pro‐androgenic sex hormone profiles in females on adverse markers of left ventricular structure and function typically associated with HF with preserved ejection fraction and with HF. There was weaker evidence of association in males.

## Introduction

Heart failure (HF) is a major global health threat, affecting 64.3 million worldwide.^1^, ^2^ Trends in lifetime HF risk vary substantially between sexes, suggesting a potential role of sex hormones in its pathogenesis.

Hyperandrogenic states, characterized by increased levels of bioavailable testosterone, commonly occur in both males and females due to physiological, pathological, and iatrogenic factors. Physiologically, females are known to switch to a proportionally more pro‐androgenic hormonal profile after the menopause,^3^ and a transient physiological increase in testosterone levels is known to occur after exercise in both males and females.^4^ Pathologically, hyperandrogenic states might occur with adrenal tumours and, for females, in the much more common polycystic ovarian syndrome (PCOS).^5^ Finally, iatrogenic increases might occur secondary to the use of testosterone‐based hormonal therapies. In males, the use of testosterone replacement therapy peaked as high as 3.2% among adults in the United States in 2011, reducing to 1.3% in 2016 following research linking its use to heightened cardiovascular risk.^6^, ^7^ In birth‐assigned females, the use of testosterone‐based gender‐affirming hormonal therapy is also on the rise. While up‐to‐date figures regarding prescription of these therapies are currently lacking, population‐based estimates of gender incongruence for birth‐assigned female persons range from 0.4% to 1.2%.^8^ Additionally, multiple hormone replacement therapy (HRT) preparations used during the peri‐menopause or post‐menopause contain testosterone.^9^

The cardiovascular associations of hyperandrogenism vary between males and females. In males, an inverse association between androgen levels and cardiovascular risk has been reported,^10^ and concordantly, androgen deprivation therapies have been associated with improved cardiometabolic risk profiles.^11^ However, prior studies have reported a paradoxically higher rate of major cardiovascular events.^7^ Additionally, randomized studies of active testosterone administration among at‐risk males have been generally null,^12^, ^13^ and some even describe harm.^14^, ^15^ The relationship is more complex in females. Observational studies in pre‐menopausal females describe an association between pro‐androgenic hormonal profiles and cardiovascular risk,^16^ and PCOS is known to be associated with adverse cardiometabolic profile and higher cardiovascular event rates.^17^ In post‐menopausal females, results are conflicted, with some observational studies reporting a protective effect of higher androgen levels^18^, ^19^ and others a detrimental one.^20^, ^21^ Cohort studies in transgender men undergoing testosterone‐based hormone therapy have reported a higher risk of cardiovascular disease and adverse cardiometabolic profiles.^22^, ^23^

The observational nature of currently available evidence is a major limitation to causal inference because observational associations are liable to residual confounding and bias. Mendelian randomization (MR) is a method that leverages ‘natural’ randomization to high or low genetic predisposition to risk factors within an instrumental variable framework to explore the causal relevance of risk factors on an outcome. As the allocation to genetic risk is random, this is akin to randomization in a clinical trial and thus less liable to influence by observational confounding.

Improving current understanding regarding sex hormones and cardiovascular risk is a key public health priority. First, it might lead to improved life course risk stratification relating to physiological, pathological, or iatrogenic hormonal changes. Second, it may better inform care for people receiving testosterone‐based hormonal therapy by improving knowledge of cardiovascular effects.

This study aims to leverage genetic variants associated with bioavailable testosterone, as well the closely related measures of sex hormone‐binding globulin (SHBG) and total testosterone, to evaluate their causal relevance on multiple cardiac magnetic resonance (CMR) imaging markers of cardiac structure and function, and HF risk.

## Methods

### Ethics and data access

The study is reported using the methodological framework from the Strengthening the Reporting of Observational Studies in Epidemiology using Mendelian Randomization (STROBE‐MR) guidelines^24^ and is in line with recommendations outlined in the Sex and Gender Equity in Research guidelines.^25^ For the purpose of sex‐specific analyses, sex was defined as that which was genetically inferred.

Summary‐level data from publicly available genome‐wide association study (GWAS) summary statistics were used and are available to download freely from the cited publications. Relevant ethical approval and participant consent were obtained in each individual study. Individual‐level data in the UK Biobank were accessed under Application 13784. Participants provided written informed consent for data release for research purposes within approved applications. Further details on the protocol of the UK Biobank can be found in previous publications.^26^, ^27^, ^28^

All statistical analyses were performed using R Version 4.1.1 (2021‐02‐15)^29^ using the MendelianRandomization^30^ and TwoSampleMR packages.^30^

### Instrumental variable selection

**Figure 1:**
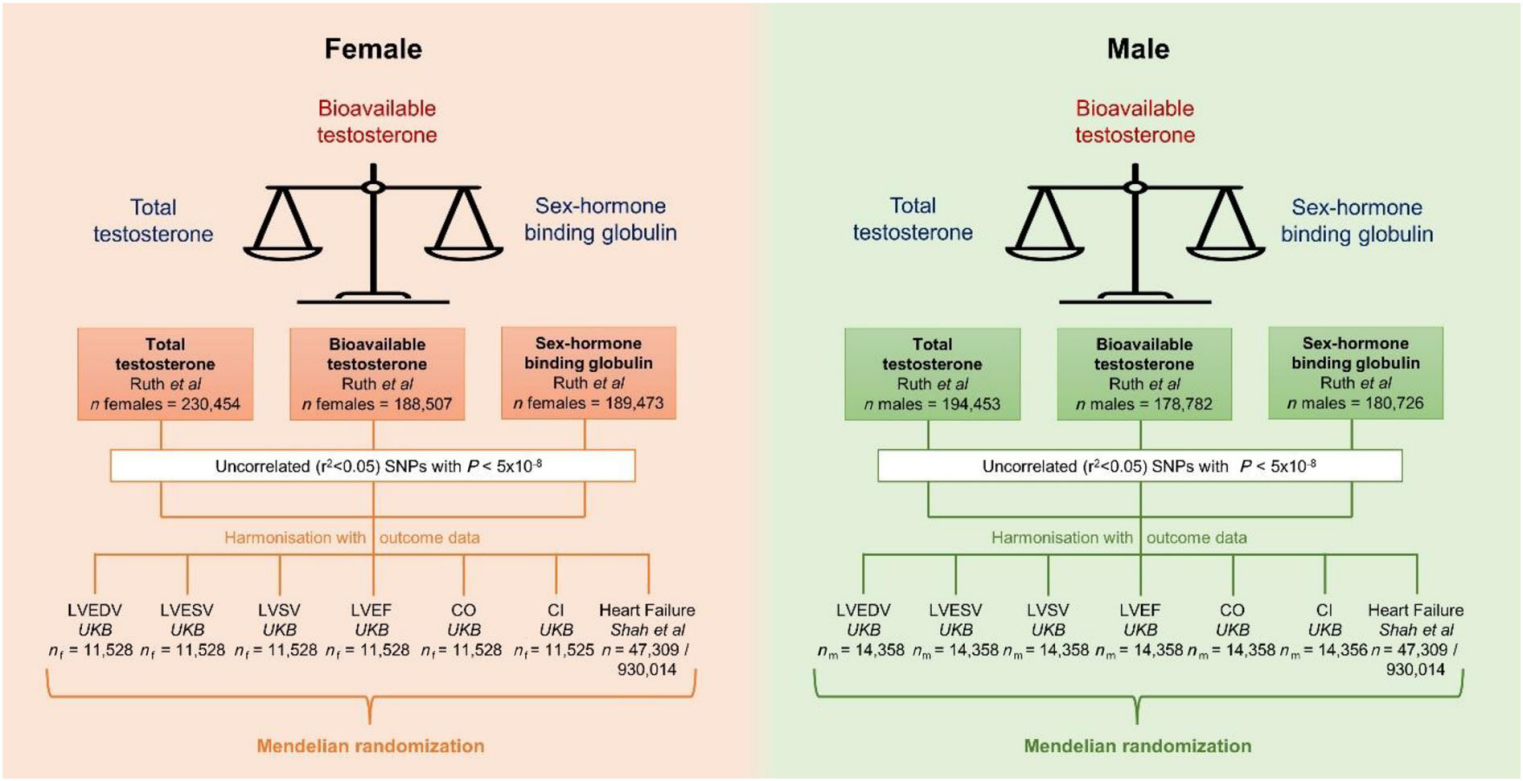
Summary of study methodology. CI, cardiac index; CO, cardiac output; LVEDV, left ventricular end‐diastolic volume; LVEF, left ventricular ejection fraction; LVESV, left ventricular end‐systolic volume; LVSV, left ventricular stroke volume; SNPs, single nucleotide polymorphisms; UKB, UK Biobank.

Sex‐specific instrumental variants were extracted from Ruth *et al*.'s GWAS on 425 097 UK Biobank participants of predominantly European ancestry.^31^ The primary exposure considered in this study was bioavailable testosterone (males *n* = 178 782, females *n* = 188 507, unit = nmol/L). As testosterone exists in a physiological equilibrium between its bioavailable form and its bound form to SHBG, we additionally studied the secondary exposures of SHBG (males *n* = 180 726, females *n* = 189 473, unit = nmol/L) and total testosterone (males *n* = 194 453, females *n* = 230 454, unit = nmol/L). Given their equilibrium state, it would be expected that any direct association observed for the exposure of bioavailable testosterone would be reflected by an inverse association for SHBG and vice versa. Sex‐specific, uncorrelated (*r* ^2^ < 0.05) single nucleotide polymorphisms (SNPs) associated with each exposure at genome‐wide significance (*P* < 5 × 10^−8^) were selected as instrumental variants. The list of instrumental variants for each exposure, with respective effect sizes and effect alleles, is provided in Supporting Information, *Tables* *S1*–*S6*. A summary of the study methodology and data sources is presented in *Figure* *1*.

### Study outcomes

Outcomes included CMR measures of cardiac structure and function, including left ventricular (LV) end‐systolic volume (LVESV), LV end‐diastolic volume (LVEDV), LV ejection fraction (LVEF), LV stroke volume (LVSV), cardiac output (CO), and cardiac index (CI), and clinical diagnosis of HF.

The methods used to derive measures of cardiac structure and function in the UK Biobank Imaging Study have been previously described.^32^ The definition of HF in the UK Biobank cohort is presented in Supporting Information, *Table* *S7*, which includes diagnoses recorded across all hospital inpatient records. Sex‐specific gene–outcome association estimates were extracted from the UK Biobank and UK Biobank Imaging Study. In females, sample sizes were as follows: 11 528 participants for LVEF, LVEDV, LVESV, LVSV, and CO and 11 525 participants for CI. In males, sample sizes were as follows: 14 358 participants for LVEF, LVEDV, LVESV, LVSV, and CO and 14 356 participants for CI. Genetic association estimates for the outcome of HF were extracted from the results of Shah *et al*.'s GWAS on 47 309 HF cases and 930 014 controls.^33^ This GWAS was not sex‐specific, but the association estimates were adjusted for sex. Validation analysis was performed on sex‐specific data from the UK Biobank to look for consistency of estimates, including 4128 cases and 194 687 controls for females and 7713 cases and 161 014 controls for males.

### Mendelian randomization

Inverse‐variance weighted (IVW) MR with multiplicative random effects^34^ was used for primary analysis for all models.^35^ Results are presented as odds ratios (ORs) with 95% confidence intervals for each unit increment in genetically predicted exposure on HF risk. For the CMR outcomes, results are presented as beta coefficients (*β*) and 95% confidence intervals, indicating the expected unit change in CMR parameter for each nmol/L increment in the genetically predicted exposure.

### Sensitivity analyses

Sensitivity analyses were carried out to ascertain potential violations of the instrumental variable assumptions. These include that: (i) the instruments associate with exposures of interest (relevance); (ii) do not have confounders associated with the outcome (independence); and (iii) no horizontal/direct pleiotropic effects exist (exclusion restriction).

To assess the relevance assumption, instrument strength was quantified using *F*‐statistics, reported in Supporting Information, *Table* *S8*. To assess the exclusion restriction assumption, sensitivity analysis was carried out using weighted median MR and MR‐Egger.^36^ The weighted mean estimate has been shown to provide consistent results in simulations when up to 50% of instrumental variants included in the analysis violate this assumption.^37^ The MR‐Egger test detects evidence of directional pleiotropy under a weaker assumption that the instrument strength is independent of direct effects (InSIDE assumption)^36^ through the introduction of an intercept term.

Where evidence of potential pleiotropy was discovered, instrumental variants were examined in more detail through a phenome‐wide association search using PhenoScanner (Supporting Information, *Table* *S9*). Among phenotypes associated with instrumental variants, candidate phenotypes to adjust for in multivariable MR (MVMR^38^) analyses were chosen based on prior knowledge of causal associations of the phenotypes with HF. Association estimates between genetically predicted sex hormones and the outcome in question were adjusted by the associations of the instrumental SNPs with body mass index (BMI) and systolic blood pressure (SBP), extracted respectively from Pulit *et al*.'s^39^ (*n* = 806 834 participants) and Evangelou *et al*.'s^40^ (*n* = 738 168 participants) GWAS summary statistics. The strength of instruments for the sex hormone trait in the modified multivariable analyses was quantified using conditional *F*‐statistics.^41^

## Results

### Females

#### Measures of cardiac structure

In females, higher genetically predicted bioavailable testosterone was associated with lower LVEDV [*β* per nmol/L = −0.11 (−0.19 to −0.03), *P* = 0.006], lower LVESV [*β* = −0.09 (−0.17 to −0.01), *P* = 0.022], and lower LVSV [*β* = −0.11 (−0.18 to −0.03), *P* = 0.005]. As would be expected, the associations for SHBG were in the opposite direction: genetically predicted SHBG was associated with higher LVEDV [*β* per nmol/L = 0.17 (0.08–0.25), *P* = 2 × 10^−4^], higher LVESV [*β* = 0.13 (0.05–0.22), *P* = 0.003], and higher LVSV [*β* = 0.18 (0.08–0.28), *P* = 2 × 10^−4^]. Higher genetically predicted total testosterone was associated with higher LVEDV [*β* per nmol/L = 0.06 (0.00–0.11), *P* = 0.036], but no significant difference in LVESV or LVSV.

**Figure 2:**
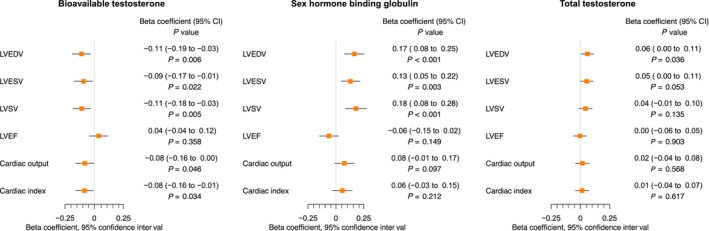
Cardiovascular magnetic resonance in females. Mendelian randomization estimates for the effects of bioavailable testosterone, sex hormone‐binding globulin, and total testosterone on cardiac magnetic resonance outcomes in females. CI, confidence interval; LVEDV, left ventricular end‐diastolic volume; LVEF, left ventricular ejection fraction; LVESV, left ventricular end‐systolic volume; LVSV, left ventricular stroke volume.

The results are reported in *Figure* *2* and *Table* *1*. Sensitivity analyses revealed evidence of potential directional pleiotropy in the association between bioavailable testosterone and LVSV [MR‐Egger intercept test *P* = 0.043; MR‐Egger *β* = 0.01 (−0.13 to 0.15), *P* = 0.933], as displayed in *Table* *2*. Adjustment for genetically predicted SBP on multivariable analysis did not attenuate the association between bioavailable testosterone and LVSV [*β* = −0.12 (−0.21 to −0.03), *P* = 0.0.013, conditional *F*‐statistic = 65.4]; however, adjustment for BMI did [*β* = 0.01 (−0.12 to 0.10), *P* = 0.843, conditional *F*‐statistic = 37.7], indicating likely pleiotropy through BMI.

**Table 1:** Mendelian randomization estimates for the effects of androgenic sex hormones on cardiovascular outcomes, using an inverse‐variance weighted model

Exposure	Outcome	SNPs	*β*/(*OR)	Lower 95% CI	Upper 95% CI	*P* value
Female bioavailable testosterone (per 1‐standard deviation increase)	LVEF	175	0.04	−0.04	0.12	0.358
	LVEDV	175	−0.11	−0.19	−0.03	0.006
	LVESV	175	−0.09	−0.17	−0.01	0.022
	LVSV	175	−0.11	−0.18	−0.03	0.005
	Cardiac output	175	−0.08	−0.16	0.00	0.046
	Cardiac index	175	−0.08	−0.16	−0.01	0.034
	Heart failure^a^	175	1.13	0.96	1.34	0.147
Female total testosterone (per 1‐standard deviation increase)	LVEF	243	0.00	−0.06	0.05	0.903
	LVEDV	243	0.06	0.00	0.11	0.036
	LVESV	243	0.05	0.00	0.11	0.053
	LVSV	242	0.04	−0.01	0.10	0.135
	Cardiac output	243	0.02	−0.04	0.08	0.568
	Cardiac index	243	0.01	−0.04	0.07	0.617
	Heart failure^a^	243	1.08	0.96	1.21	0.199
Female sex hormone‐binding globulin (per 1‐standard deviation increase)	LVEF	348	−0.06	−0.15	0.02	0.149
	LVEDV	347	0.17	0.08	0.25	2 × 10^−4^
	LVESV	348	0.13	0.05	0.22	0.003
	LVSV	347	0.18	0.08	0.28	2 × 10^−4^
	Cardiac output	347	0.08	−0.01	0.17	0.097
	Cardiac index	347	0.06	−0.03	0.15	0.212
	Heart failure^a^	348	0.95	0.80	1.13	0.544
Male bioavailable testosterone (per 1‐standard deviation increase)	LVEF	121	−0.11	−0.18	−0.04	0.003
	LVEDV	121	−0.03	−0.1	0.03	0.322
	LVESV	121	−0.03	−0.09	0.03	0.367
	LVSV	121	−0.02	−0.09	0.04	0.462
	Cardiac output	121	−0.01	−0.08	0.05	0.681
	Cardiac index	121	−0.01	−0.08	0.05	0.719
	Heart failure^a^	103	0.94	0.82	1.06	0.314
Male total testosterone (per 1‐standard deviation increase)	LVEF	226	−0.01	−0.06	0.04	0.766
	LVEDV	225	−0.04	−0.09	0.01	0.090
	LVESV	225	−0.07	−0.12	−0.02	0.007
	LVSV	226	−0.01	−0.06	0.03	0.567
	Cardiac output	226	−0.01	−0.06	0.04	0.732
	Cardiac index	226	−0.01	−0.06	0.04	0.786
	Heart failure^a^	216	0.94	0.86	1.02	0.125
Male sex hormone‐binding globulin (per 1‐standard deviation increase)	LVEF	353	0.04	−0.06	0.13	0.446
	LVEDV	352	0.00	−0.09	0.09	0.964
	LVESV	352	−0.02	−0.11	0.07	0.724
	LVSV	353	0.01	−0.08	0.10	0.880
	Cardiac output	353	0.00	−0.09	0.09	0.981
	Cardiac index	353	−0.01	−0.10	0.08	0.873
	Heart failure^a^	338	1.05	0.90	1.21	0.563

CI, confidence interval; LVEDV, left ventricular end‐diastolic volume; LVEF, left ventricular ejection fraction; LVESV, left ventricular end‐systolic volume; LVSV, left ventricular stroke volume; OR, odds ratio; SNPs, single nucleotide polymorphisms.

^a^ Outcomes where odds ratio is presented.

**Table 2:** Mendelian randomization (MR) sensitivity analyses for the effects of reproductive factors on cardiovascular outcomes, using weighted median MR and MR‐Egger models

Exposure	Outcome	Method	SNPs	*β*/OR	Lower 95% CI	Upper 95% CI	*P* value
Female bioavailable testosterone (per 1‐standard deviation increase)	LVEF	Weighted median	175	0.00	−0.13	0.14	0.952
		MR‐Egger	175	0.09	−0.05	0.23	0.211
						Intercept	0.373
	LVEDV	Weighted median	175	−0.07	−0.20	0.06	0.283
		MR‐Egger	175	−0.04	−0.18	0.10	0.604
						Intercept	0.212
	LVESV	Weighted median	175	−0.09	−0.22	0.04	0.186
		MR‐Egger	175	−0.05	−0.19	0.09	0.511
						Intercept	0.437
	LVSV	Weighted median	175	0.01	−0.12	0.14	0.867
		MR‐Egger	175	0.01	−0.13	0.14	0.933
						Intercept	0.043
	Cardiac output	Weighted median	175	0.02	−0.11	0.14	0.815
		MR‐Egger	175	0.10	−0.03	0.24	0.143
						Intercept	0.002
	Cardiac index	Weighted median	175	−0.01	−0.14	0.11	0.821
		MR‐Egger	175	0.08	−0.06	0.21	0.249
						Intercept	0.005
	Heart failure	Weighted median	175	1.06	0.79	1.44	0.687
		MR‐Egger	175	1.21	0.90	1.62	0.215
						Intercept	0.589
Female total testosterone (per 1‐standard deviation increase)	LVEF	Weighted median	243	−0.04	−0.15	0.07	0.472
		MR‐Egger	243	0.03	−0.07	0.13	0.575
						Intercept	0.450
	LVEDV	Weighted median	243	0.09	−0.02	0.20	0.108
		MR‐Egger	243	0.04	−0.06	0.14	0.439
						Intercept	0.644
	LVESV	Weighted median	243	0.10	−0.01	0.21	0.069
		MR‐Egger	243	0.04	−0.06	0.14	0.435
						Intercept	0.725
	LVSV	Weighted median	242	0.03	−0.09	0.14	0.651
		MR‐Egger	242	0.02	−0.08	0.13	0.696
						Intercept	0.599
	Cardiac output	Weighted median	243	0.00	−0.11	0.12	0.942
		MR‐Egger	243	0.01	−0.10	0.11	0.905
						Intercept	0.813
	Cardiac index	Weighted median	243	−0.04	−0.14	0.07	0.478
		MR‐Egger	243	−0.02	−0.12	0.09	0.779
						Intercept	0.502
	Heart failure	Weighted median	243	1.28	1.06	1.54	0.009
		MR‐Egger	243	1.33	1.08	1.64	0.007
						Intercept	0.017
Female sex hormone‐binding globulin (per 1‐standard deviation increase)	LVEF	Weighted median	348	−0.06	−0.21	0.08	0.411
		MR‐Egger	348	−0.08	−0.21	0.05	0.219
						Intercept	0.735
	LVEDV	Weighted median	347	0.06	−0.09	0.22	0.440
		MR‐Egger	347	0.13	0.01	0.26	0.041
						Intercept	0.513
	LVESV	Weighted median	348	0.07	−0.07	0.22	0.323
		MR‐Egger	348	0.13	0.00	0.25	0.044
						Intercept	0.971
	LVSV	Weighted median	347	−0.01	−0.17	0.16	0.925
		MR‐Egger	347	0.09	−0.05	0.23	0.224
						Intercept	0.075
	Cardiac output	Weighted median	347	−0.13	−0.29	0.04	0.133
		MR‐Egger	347	−0.04	−0.17	0.09	0.553
						Intercept	0.018
	Cardiac index	Weighted median	347	−0.13	−0.29	0.03	0.106
		MR‐Egger	347	−0.05	−0.18	0.09	0.492
						Intercept	0.035
	Heart failure	Weighted median	348	0.95	0.70	1.29	0.750
		MR‐Egger	348	0.90	0.69	1.16	0.419
						Intercept	0.588
Male bioavailable testosterone (per 1‐standard deviation increase)	LVEF	Weighted median	121	−0.18	−0.28	−0.07	0.001
		MR‐Egger	121	−0.18	−0.29	−0.07	0.002
						Intercept	0.118
	LVEDV	Weighted median	121	−0.07	−0.17	0.04	0.206
		MR‐Egger	121	−0.10	−0.20	0.00	0.065
						Intercept	0.112
	LVESV	Weighted median	121	−0.08	−0.19	0.02	0.128
		MR‐Egger	121	−0.06	−0.17	0.04	0.220
						Intercept	0.393
	LVSV	Weighted median	121	−0.06	−0.17	0.04	0.256
		MR‐Egger	121	−0.09	−0.19	0.02	0.098
						Intercept	0.124
	Cardiac output	Weighted median	121	−0.03	−0.14	0.07	0.517
		MR‐Egger	121	−0.07	−0.17	0.03	0.168
						Intercept	0.148
	Cardiac index	Weighted median	121	−0.04	−0.15	0.06	0.414
		MR‐Egger	121	−0.08	−0.18	0.03	0.146
						Intercept	0.114
	Heart failure	Weighted median	103	0.84	0.70	1.01	0.064
		MR‐Egger	103	0.96	0.76	1.20	0.711
						Intercept	0.814
Male total testosterone (per 1‐standard deviation increase)	LVEF	Weighted median	226	0.04	−0.06	0.13	0.424
		MR‐Egger	226	0.03	−0.05	0.11	0.452
						Intercept	0.225
	LVEDV	Weighted median	225	−0.09	−0.18	−0.01	0.033
		MR‐Egger	225	−0.03	−0.11	0.05	0.463
						Intercept	0.655
	LVESV	Weighted median	225	−0.12	−0.20	−0.03	0.007
		MR‐Egger	225	−0.05	−0.13	0.03	0.198
						Intercept	0.592
	LVSV	Weighted median	226	−0.06	−0.14	0.03	0.194
		MR‐Egger	226	−0.01	−0.08	0.07	0.857
						Intercept	0.812
	Cardiac output	Weighted median	226	−0.05	−0.14	0.03	0.231
		MR‐Egger	226	0.00	−0.08	0.08	0.994
						Intercept	0.770
	Cardiac index	Weighted median	226	−0.05	−0.13	0.04	0.290
		MR‐Egger	226	0.00	−0.08	0.07	0.949
						Intercept	0.887
	Heart failure	Weighted median	216	0.90	0.78	1.03	0.127
		MR‐Egger	216	1.03	0.90	1.18	0.673
						Intercept	0.071
Male sex hormone‐binding globulin (per 1‐standard deviation increase)	LVEF	Weighted median	353	0.17	0.01	0.33	0.037
		MR‐Egger	353	0.13	−0.01	0.26	0.060
						Intercept	0.057
	LVEDV	Weighted median	352	0.00	−0.15	0.15	0.986
		MR‐Egger	352	−0.03	−0.16	0.09	0.615
						Intercept	0.499
	LVESV	Weighted median	352	−0.14	−0.30	0.01	0.074
		MR‐Egger	352	−0.09	−0.22	0.03	0.154
						Intercept	0.093
	LVSV	Weighted median	353	−0.03	−0.17	0.11	0.634
		MR‐Egger	353	0.01	−0.11	0.14	0.831
						Intercept	0.879
	Cardiac output	Weighted median	353	−0.06	−0.22	0.09	0.406
		MR‐Egger	353	0.02	−0.10	0.14	0.768
						Intercept	0.654
	Cardiac index	Weighted median	353	−0.08	−0.22	0.06	0.257
		MR‐Egger	353	0.02	−0.11	0.14	0.786
						Intercept	0.579
	Heart failure	Weighted median	338	1.20	0.95	1.51	0.122
		MR‐Egger	338	0.97	0.78	1.20	0.760
						Intercept	0.310

CI, confidence interval; LVEDV, left ventricular end‐diastolic volume; LVEF, left ventricular ejection fraction; LVESV, left ventricular end‐systolic volume; LVSV, left ventricular stroke volume; OR, odds ratio; SNPs, single nucleotide polymorphisms.

#### Measures of cardiac function

In females, higher genetically predicted bioavailable testosterone was associated with a lower CO [*β* = −0.08 (−0.16 to 0.00), *P* = 0.046] and a lower CI [*β* = −0.08 (−0.16 to −0.01), *P* = 0.034]. No associations were evident for the secondary exposures of genetically predicted SHBG and genetically predicted total testosterone with either outcome, and none of the exposures were associated with LVEF. The results are reported in *Figure* *2* and *Table* *1*.

Sensitivity analyses revealed potential directional pleiotropy in the association of bioavailable testosterone with CO (MR‐Egger intercept test *P* = 0.002) and CI (MR‐Egger intercept test *P* = 0.005), as displayed in *Table* *2*. Adjustment for genetically predicted SBP on multivariable analysis mildly attenuated the association between bioavailable testosterone and CO [*β* = −0.08 (0.01 to −0.17), *P* = 0.062, conditional *F*‐statistic = 65.4] and did not attenuate the association with CI [*β* = −0.10 (−0.18 to −0.01), *P* = 0.021, conditional *F*‐statistic = 65.4]. However, adjustment for BMI attenuated the association with both CO [*β* = 0.09 (−0.02 to 0.19), *P* = 0.112, conditional *F*‐statistic = 37.7] and CI [*β* = 0.06 (−0.05 to 0.17), *P* = 0.021, conditional *F*‐statistic = 37.7], indicating likely pleiotropy through BMI.

#### Heart failure

Higher genetically predicted bioavailable testosterone in females was associated with higher odds of HF [OR 1.10 (1.01–1.19), *P* = 0.026]. In the validation analysis on the smaller UK Biobank cohort, this association was not significant though the association estimate remained consistent in both direction and magnitude [OR 1.13 (0.96–1.34), *P* = 0.147].

**Figure 3:**
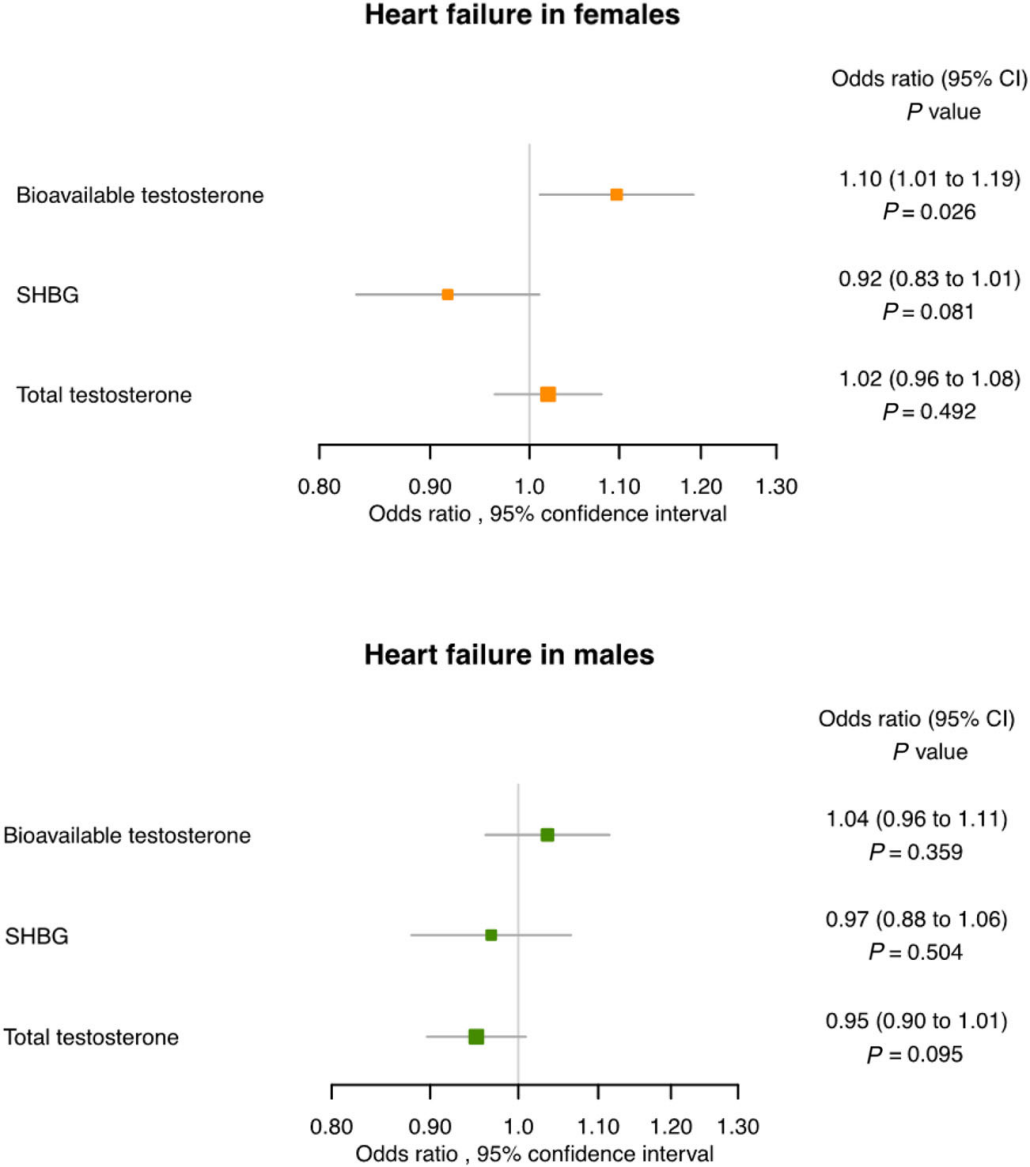
Heart failure in females and males. Mendelian randomization estimates for the effects of bioavailable testosterone, total testosterone, and sex hormone‐binding globulin (SHBG) on the risk of heart failure in females and males. CI, confidence interval.

There was no significant association with HF for genetically predicted SHBG and genetically predicted total testosterone in both the primary and validation analyses, as reported in *Tables* *1* and *3* and *Figure* *3*.

**Table 3:** Mendelian randomization estimates for the effects of reproductive factors on heart failure, using an inverse‐variance weighted model

Exposure	Outcome	Number of SNPs	Odds ratio	Lower 95% CI	Upper 95% CI	*P* value
Female bioavailable testosterone	Heart failure	132	1.10	1.01	1.19	0.026
Female total testosterone		185	1.02	0.96	1.08	0.492
Female sex hormone‐binding globulin		275	0.92	0.83	1.01	0.081
Male bioavailable testosterone		82	1.04	0.96	1.11	0.359
Male total testosterone		154	0.95	0.90	1.01	0.095
Male sex hormone‐binding globulin		256	0.97	0.88	1.06	0.504

CI, confidence interval; SNPs, single nucleotide polymorphisms.

### Males

#### Measures of cardiac structure

**Figure 4:**
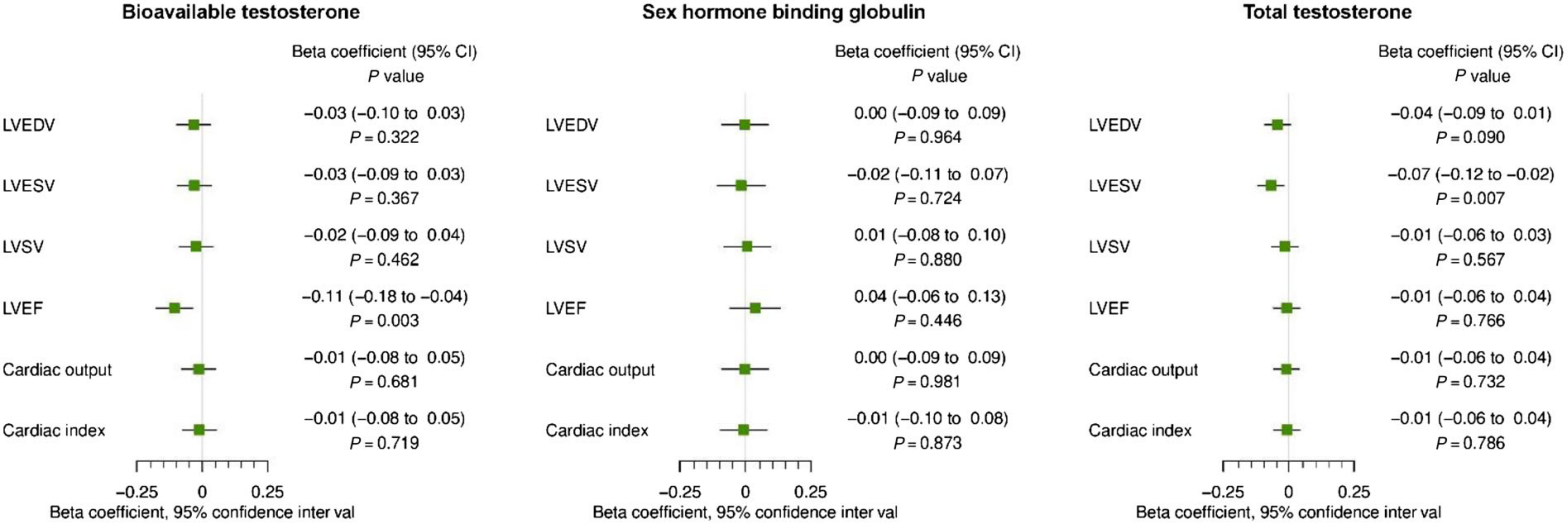
Cardiovascular magnetic resonance in males. Mendelian randomization estimates for the effects of bioavailable testosterone, sex hormone‐binding globulin, and total testosterone on cardiac magnetic resonance outcomes in males. CI, confidence interval; LVEDV, left ventricular end‐diastolic volume; LVEF, left ventricular ejection fraction; LVESV, left ventricular end‐systolic volume; LVSV, left ventricular stroke volume.

Higher genetically predicted total testosterone was associated with a lower LVESV in males [*β* = −0.07 (−0.12 to −0.02), *P* = 0.007], but no associations were observed for genetically predicted bioavailable testosterone and SHBG, as reported in *Figure* *4*. Sensitivity analyses were consistent with the primary analyses, as displayed in *Table* *2*. In males, none of the exposures were associated with either LVEDV or LVSV. The results are reported in *Figure* *3*.

#### Measures of cardiac function

In males, higher genetically predicted bioavailable testosterone was associated with a lower LVEF [*β* = −0.11 (−0.18 to −0.04), *P* = 0.003]. No associations with LVEF were observed for genetically predicted SHBG and total testosterone. Additionally, as displayed in *Figure* *4*, none of the genetically predicted exposures were associated with CO or CI.

#### Heart failure

In the analysis on the external cohort, higher genetically predicted bioavailable testosterone was not associated with HF [OR 1.04 (0.96–1.11), *P* = 0.359], and neither were genetically predicted total testosterone or SHBG (*Table* *3* and *Figure* *4*). The null results were consistent in the validation analysis in the UK Biobank, as reported in *Table* *1*.

## Discussion

In this study, we explored associations between androgenic sex hormone profiles and multiple markers of cardiac structure and function. A pro‐androgenic profile was associated with adverse LV remodelling with features of greater cardiac ageing broadly reflective of an HF with preserved ejection fraction (HFpEF) phenotype. These relationships appeared more prominent in females, in whom higher bioavailable testosterone (and lower SHBG) was associated with a smaller structure (lower LVEDV, LVESV, and LVSV) and function (lower LVSV, CO, and CI). A pro‐androgenic profile was associated with a greater likelihood of clinical HF in females, but not in males. Thus, our results demonstrate androgenic hormones as a proponent of poorer cardiovascular health, in particular HF, which appears more important in females than males.

Several observational studies have explored the association between androgenic sex hormone profiles and cardiovascular risk in females, linking to both menopausal and pathological conditions. Post‐menopausal females exhibit proportionally more androgenic sex hormone profiles, which has been, albeit inconsistently,^21^, ^42^, ^43^ suggested to associate with increased cardiovascular risk.^19^, ^20^ This pattern is reflected in females with PCOS, who exhibit higher absolute androgen levels and have also been reported to suffer higher risk of cardiovascular disease. However, drawing causal inference from observational studies is limited due to the residual confounding. The present study provides genetic evidence to support a causal role of higher androgen levels in females on adverse cardiac remodelling. This study adds to current literature as the first to employ MR to explore this question, limiting the influence of observational confounding and therefore providing more reliable evidence of causal relationships.

There are several potential mechanisms behind the findings of this study. Females have a 20‐fold to 25‐fold lower circulating androgen concentration compared with males.^44^ Long‐term androgen receptor activation in females has been shown to predispose to insulin resistance, combined with up‐regulation of genes involved in HDL catabolism leading to dyslipidaemia.^16^ Indeed, Ruth *et al*. have previously described an association between higher androgen levels and worse cardiometabolic profiles in females using MR.^31^ Additionally, hormonal changes occurring in the peri‐menopause, when androgenic transient hormones increase and then decline to 15% of pre‐menopausal levels post‐menopause,^44^ have been shown to accelerate adverse arterial remodelling through proliferation of vascular smooth muscle cells,^45^, ^46^ leading to greater arterial stiffness and thus accelerated age‐related hypertension, contributing to augmented cardiovascular risk. Physiological levels of testosterone increase nitric oxide synthesis through gene induction, which is not seen with high levels of testosterone.^46^ Finally, the menopause transition and its concurrent proportionally androgenic state have been associated with significant changes in body composition,^47^ including increased deposition of visceral fat. Increased visceral fat, especially in the pericardial compartment^48^ and abdominal area,^46^ might further augment cardiovascular risk through both endocrine and inflammatory paracrine mechanisms. It is important to note that the above mechanisms form a complex picture and existing research is contradictory alluding to multifactorial explanations linking androgens to cardiovascular risk.

In males, higher androgen levels were associated with reduced LVEF and LVESV. These results are contradictory with a prior MR analysis^49^ and also with available observational evidence. In the prior MR analysis, genetically predicted testosterone was associated with HF.^49^ The different findings could be attributed to the different health statuses in their study population compared with the UK Biobank, comprising a relatively healthy cohort. Furthermore, there were low numbers of participants with HF used in the prior study^49^ and survival bias could have played a part in the UK Biobank. In observational studies, androgen deprivation use as a treatment for prostate cancer^50^ or due to hypogonadism,^7^, ^51^, ^52^ have been associated with cardiovascular risk. A potential explanation for this apparent equivocality might relate to non‐linear effects, whereby those with both extremely low and high androgen levels suffer increased risk. Overall, given the important inconsistencies in available evidence, cardiac pharmacovigilance in the context of both androgen‐depriving and androgen‐enhancing therapies in males remains an important priority, and further research is needed to explore potential non‐linear effects or establish effect modifiers.

In this study, we identified potential pleiotropic effects in the association between bioavailable testosterone and cardiac function in females. This finding requires further discussion. First, this only involves one of the exposures, bioavailable testosterone, and the specific outcomes of LVSV, CO, and CI (not LVESV, LVEDV, and HF). Given the biologically plausible and consistent results observed across other outcomes and exposures, the potential pleiotropy discovered in some specific associations does not substantially alter the broader interpretation of study findings. Second, we further explored this by highlighting potential culprit phenotypes of BMI and SBP and demonstrated a more likely role of BMI in the pleiotropic effects. This suggests that BMI might either directly or indirectly be responsible for at least part of the observed associations, highlighting it as a key target for surveillance and risk modification.

These results carry important clinical implications. First, the results support a potentially adverse mechanistic role of androgens in the cardiovascular association of multiple pathological states characterized by higher androgen levels in females, such as PCOS, and cardiac disease. The results therefore encourage further research to elucidate whether androgens are an important or primary mediator of this association, as they represent therapeutic targets amenable to intervention. Second, they support further research exploring the long‐term cardiovascular health of individuals taking testosterone‐based hormone treatments. These include post‐menopausal females taking HRT preparations that contain testosterone, as well as individuals undergoing gender‐affirming hormone treatments. Gender‐affirming hormone treatment use has increased significantly in recent years, and creating a high‐quality evidence base regarding long‐term cardiovascular health should be a key priority to ensure high standards of care. Evidence among existing studies is conflicting, with some suggesting an increased risk of cardiovascular disease in transgender males undergoing hormone therapy^53^, ^54^ yet others suggesting no effect.^22^, ^55^, ^56^ Importantly, higher risk of hypertension has been reported, suggesting mechanistic effects on the vascular system consistent with the HFpEF imaging phenotype observed in this study.

There are several limitations to discuss. First, magnitude of effect estimates for reported associations cannot be interpreted intuitively as real‐life unit changes in outcome expected per unit change in the exposure through pharmacological modification. Measures of exposures in MR studies reflect a lifetime exposure, rather than a time‐limited modification in the exposure secondary to pharmacotherapy. Second, we were unable to expand the analysis to other sex hormones (e.g. oestradiol and progesterone) and additional measures of cardiac structure and function (e.g. LV mass) due to the lack of sufficiently powered data available. This should be an important target for future research. Finally, in this study, we utilized two‐sample MR methods in a one‐sample setting as both gene–exposure and gene–outcome association data were derived from the UK Biobank. However, this has been shown to be unlikely to significantly bias analyses in large‐scale biobanks.^57^

## Conclusions

In conclusion, these data support a causal association between bioavailable testosterone in females and adverse measures of cardiac structure and function in a pattern broadly consistent with HFpEF, as well as a higher risk of HF. In males, higher androgen levels were associated with reduced LVEF and LVESV. The results call for further investigation of the prognostic value of sex‐specific risk factors typically associated with pro‐androgenic states (e.g. PCOS) for cardiovascular risk stratification of females. By highlighting a likely causal role of testosterone, they also encourage exploration of whether this might be a modifiable mediator of the increased cardiovascular risk that is known to occur in PCOS. Additionally, they encourage pharmacovigilance and ongoing research to characterize the long‐term cardiovascular health of individuals undergoing testosterone‐based therapies for both gender affirmation and hormone replacement.

## Conflict of interest

None declared.

## Funding

This work was supported by core funding from the British Heart Foundation (BHF) (RG/13/13/30194 and RG/18/13/33946), BHF Cambridge Centre of Research Excellence (RE/18/1/34212), and NIHR Cambridge Biomedical Research Centre (BRC‐1215‐20014). This work was also supported by Health Data Research UK, which is funded by the UK Medical Research Council, Engineering and Physical Sciences Research Council, Economic and Social Research Council, Department of Health and Social Care (England), Chief Scientist Office of the Scottish Government Health and Social Care Directorates, Health and Social Care Research and Development Division (Welsh Government), Public Health Agency (Northern Ireland), BHF, and Wellcome. M.A. was supported by the NIHR Academic Clinical Fellowship. J.Y.C. and R.K.R. were supported by the NIHR Imperial Biomedical Research Centre HEE Specialised Foundation post. A.M.M. was supported by the EU/EFPIA Innovative Medicines Initiative Joint Undertaking BigData@Heart (Grant No. 116074). Z.R.‐E. was supported by the BHF Clinical Research Training Fellowship (Grant No. FS/17/81/33318). S.B. was supported by the Sir Henry Dale Fellowship jointly funded by the Wellcome Trust and the Royal Society (Grant No. 204623/Z/16/Z). E.D.A. has no relevant funding. F.S.N. was supported by the NIHR Imperial Biomedical Research Centre funding and the BHF (RG/16/3/32175).

## Supporting information


**Table S1.** Genome‐wide significant single nucleotide polymophisms (SNP) used as instrumental variants for bioavailable testosterone in females.
**Table S2.** Genome‐wide significant single nucleotide polymorphisms (SNP) used as instrumental variants for total testosterone in females.
**Table S3.** Genome‐wide significant single nucleotide polymorphisms (SNP) used as instrumental variants for sex hormone binding globulin in females.
**Table S4.** Genome‐wide significant single nucleotide polymorphisms (SNP) used as instrumental variants for bioavailable testosterone in males.
**Table S5.** Genome‐wide significant single nucleotide polymorphisms (SNP) used as instrumental variants for total testosterone in males.
**Table S6.** Genome‐wide significant single nucleotide polymorphisms (SNP) used as instrumental variants for sex hormone binding globulin in males.
**Table S7.** Definitions for heart failure used within this study, based on the World Health Organization International Statistical Classification of Diseases and Related Health Problems 10th Revision (ICD‐10) codes.
**Table S8.** F‐statistics for instrument strength in all analyses.
**Table S9.** Genome‐wide significant (*P* < 5.10^−8^) associations of all instrumental variants used for bioavailable testosterone in females.
