# Supplementary material for: Genetically predicted androgenic profiles and adverse cardiac markers: a sex‐specific Mendelian randomization study
Source: ESC Heart Fail. 2023 Sep 22;10(6):3525–37. doi: 10.1002/ehf2.14527 (PMC10682908; doi:10.1002/ehf2.14527)
Supplement: Supplementary file 1 — Table S1. Genome‐wide significant single nucleotide polymophisms (SNP) used as instrumental variants for bioavailable testosterone in females. Table S2. Genome‐wide significant single nucleotide polymorphisms (SNP) used as instrumental variants for total testosterone in females. Table S3. Genome‐wide significant single nucleotide polymorphisms (SNP) used as instrumental variants for sex hormone binding globulin in females. Table S4. Genome‐wide significant single nucleotide polymorphisms (SNP) used as instrumental variants for bioavailable testosterone in males. Table S5. Genome‐wide significant single nucleotide polymorphisms (SNP) used as instrumental variants for total testosterone in males. Table S6. Genome‐wide significant single nucleotide polymorphisms (SNP) used as instrumental variants for sex hormone binding globulin in males. Table S7. Definitions for heart failure used within this study, based on the World Health Organization International Statistical Classification of Diseases and Related Health Problems 10th Revision (ICD‐10) codes. Table S8. F‐statistics for instrument strength in all analyses. Table S9. Genome‐wide significant (P < 5.10−8) associations of all instrumental variants used for bioavailable testosterone in females. [file EHF2-10-3525-s001.docx]

**Supplementary Data**

# Genetically predicted androgenic profiles and adverse cardiac markers – a sex-specific Mendelian randomisation study

# Jun Yu Chen*^1^, Maddalena Ardissino*^1^, Rohin K Reddy^1^, Amy Marie Mason^2^, Emanuele Di Angelantonio^3^, Stephen Burgess^2,3^, Fu Siong Ng^1^

*co-first authors with equal contribution

^1^National Heart and Lung Institute, Imperial College London, London, UK

### ^2^Medical Research Council Biostatistics Unit, University of Cambridge, Cambridge, UK

### ^3^Cardiovascular Epidemiology Unit, Department of Public Health and Primary Care, University of Cambridge, Cambridge, UK

**Contents**

**Table S1** – Genome-wide significant single nucleotide polymophisms (SNP) used as instrumental variants for bioavailable testosterone in females. ………………………......……………………………...…………………...…….3

**Table S2** – Genome-wide significant single nucleotide polymorphisms (SNP) used as instrumental variants for total testosterone in females.. ………………………......……………………………...…………………………………….15

**Table S3** – Genome-wide significant single nucleotide polymorphisms (SNP) used as instrumental variants for sex hormone binding globulin in females.. ………………………………..…………………..................………………...28

**Table S4** – Genome-wide significant single nucleotide polymorphisms (SNP) used as instrumental variants for bioavailable testosterone in males.. ………………………......……………………………...…………………...…….46

**Table S5** – Genome-wide significant single nucleotide polymorphisms (SNP) used as instrumental variants for total testosterone in males.. ………………………......……………………………...…………………………...……….53

**Table S6** – Genome-wide significant single nucleotide polymorphisms (SNP) used as instrumental variants for sex hormone binding globulin in males………………………………….………………………......……………………………...…….……………….65

**Table S7 –** Definitions for heart failure used within UK Biobank, based on the World Health Organization International Statistical Classification of Diseases and Related Health Problems 9^th^ and 10th Revision (ICD-9 and ICD-10) codes…………………………………..…………………..…...……………………………..…...……...…...83

**Table S8 –** F-statistics for instrument strength in all analyses.………………………………………….. …...…….…85

**Table S9 –** Genome-wide significant (p<5.10^-8^) associations of all instrumental variants used for bioavailable testosterone in females.……… ………..……..……………………...……………………………..……………..……86

**Table S1** – Genome-wide significant single nucleotide polymophisms (SNP) used as instrumental variants for bioavailable testosterone in females.

| **SNP** | **Chr** | **Pos** | **Effect allele** | **Non-effect allele** | **Effect allele frequency** | **Beta** | **Standard error** | **P-value** |
| --- | --- | --- | --- | --- | --- | --- | --- | --- |
| rs1989147 | 1 | 7909373 | C | T | 0.807 | 0.024 | 0.003 | 7.00E-14 |
| rs114165349 | 1 | 27021913 | C | G | 0.024 | 0.093 | 0.008 | 3.80E-28 |
| rs12135478 | 1 | 39539043 | G | A | 0.331 | 0.015 | 0.003 | 8.80E-09 |
| rs529143892 | 1 | 51087836 | C | CA | 0.644 | 0.019 | 0.003 | 1.30E-12 |
| rs2391139 | 1 | 92727610 | T | G | 0.202 | 0.025 | 0.003 | 2.60E-15 |
| rs7418101 | 1 | 93756192 | G | A | 0.622 | 0.016 | 0.003 | 4.10E-10 |
| rs6684361 | 1 | 101737743 | C | T | 0.307 | 0.04 | 0.003 | 5.40E-48 |
| rs140584594 | 1 | 110232983 | G | A | 0.73 | 0.021 | 0.003 | 2.40E-13 |
| rs2282248 | 1 | 111736594 | T | C | 0.328 | 0.015 | 0.003 | 1.30E-08 |
| rs351370 | 1 | 113054659 | T | C | 0.587 | 0.016 | 0.003 | 1.50E-10 |
| 1:154580015_TC_T | 1 | 154580015 | TC | T | 0.524 | 0.015 | 0.003 | 1.80E-08 |
| rs12564492 | 1 | 168234645 | A | G | 0.703 | 0.015 | 0.003 | 5.00E-09 |
| rs2266782 | 1 | 171076966 | A | G | 0.417 | 0.016 | 0.003 | 2.60E-11 |
| rs2152318 | 1 | 179293511 | T | C | 0.245 | 0.032 | 0.003 | 1.80E-27 |
| rs549035841 | 1 | 218548749 | C | CT | 0.709 | 0.016 | 0.003 | 4.00E-09 |
| rs34269793 | 2 | 12555164 | C | T | 0.053 | 0.033 | 0.006 | 2.20E-10 |
| 2:20421849_AACAC_A | 2 | 20421849 | A | AACAC | 0.36 | 0.022 | 0.003 | 1.70E-18 |
| rs62144584 | 2 | 25004937 | T | C | 0.728 | 0.019 | 0.003 | 1.60E-10 |
| rs2374456 | 2 | 43271621 | G | C | 0.584 | 0.016 | 0.003 | 7.40E-10 |
| rs13030651 | 2 | 43508205 | G | A | 0.449 | 0.017 | 0.003 | 7.00E-12 |
| rs13020003 | 2 | 48976886 | C | A | 0.327 | 0.017 | 0.003 | 4.40E-10 |
| rs9636441 | 2 | 62437709 | C | T | 0.29 | 0.017 | 0.003 | 6.70E-10 |
| rs13430258 | 2 | 62535082 | G | T | 0.534 | 0.016 | 0.003 | 1.90E-10 |
| rs3832126 | 2 | 64906752 | CT | C | 0.726 | 0.032 | 0.003 | 3.50E-29 |
| rs113247979 | 2 | 103338672 | T | C | 0.993 | 0.082 | 0.015 | 4.60E-09 |
| rs590097 | 2 | 111934107 | G | T | 0.646 | 0.034 | 0.003 | 4.90E-38 |
| rs11683361 | 2 | 112380559 | G | C | 0.807 | 0.022 | 0.003 | 2.10E-12 |
| rs371347025 | 2 | 178226530 | T | TAA | 0.395 | 0.014 | 0.003 | 8.10E-10 |
| rs78058190 | 2 | 219699999 | A | G | 0.051 | 0.041 | 0.006 | 2.00E-10 |
| rs1515098 | 2 | 227073854 | T | C | 0.68 | 0.015 | 0.003 | 9.00E-09 |
| rs3732218 | 2 | 234627304 | G | A | 0.918 | 0.025 | 0.005 | 3.10E-08 |
| rs573833 | 3 | 10534247 | T | C | 0.463 | 0.015 | 0.003 | 4.80E-09 |
| rs4135247 | 3 | 12396588 | G | A | 0.433 | 0.015 | 0.003 | 1.10E-08 |
| rs62231822 | 3 | 14423060 | C | T | 0.901 | 0.029 | 0.004 | 8.30E-12 |
| rs13072623 | 3 | 20163556 | G | A | 0.433 | 0.015 | 0.003 | 4.50E-09 |
| rs6792725 | 3 | 24520283 | A | G | 0.308 | 0.017 | 0.003 | 5.00E-10 |
| rs6788984 | 3 | 41107173 | A | G | 0.856 | 0.02 | 0.004 | 2.40E-08 |
| rs13092573 | 3 | 46988561 | T | C | 0.351 | 0.016 | 0.003 | 3.00E-09 |
| rs62263023 | 3 | 48260304 | A | T | 0.091 | 0.028 | 0.004 | 7.00E-11 |
| rs563306865 | 3 | 49528748 | GT | G | 0.377 | 0.019 | 0.003 | 1.10E-11 |
| rs77031559 | 3 | 50902213 | G | A | 0.066 | 0.027 | 0.005 | 9.20E-09 |
| rs6772177 | 3 | 52497778 | T | C | 0.172 | 0.02 | 0.003 | 2.40E-10 |
| rs687339 | 3 | 135932359 | T | C | 0.773 | 0.04 | 0.003 | 4.10E-39 |
| rs7633673 | 3 | 152084243 | G | A | 0.594 | 0.018 | 0.003 | 2.60E-13 |
| rs4368453 | 3 | 156852141 | T | C | 0.302 | 0.017 | 0.003 | 3.60E-09 |
| rs5855544 | 3 | 195942080 | T | TG | 0.429 | 0.015 | 0.003 | 3.30E-10 |
| rs4690098 | 4 | 3447156 | T | C | 0.235 | 0.027 | 0.003 | 4.00E-18 |
| rs114303452 | 4 | 3449915 | A | G | 0.989 | 0.092 | 0.012 | 1.80E-13 |
| rs12645584 | 4 | 17919066 | T | C | 0.731 | 0.017 | 0.003 | 3.90E-09 |
| 4:69468698_ATAGAGGAAGGCCACTGTCTCCTGCCTGCCCCTGGGAACTGAATGTC_A | 4 | 69468698 | A | ATAGAGGAAGGCCACTGTCTCCTGCCTGCCCCTGGGAACTGAATGTC | 0.732 | 0.023 | 0.003 | 4.70E-13 |
| rs71633359 | 4 | 88183820 | C | T | 0.318 | 0.023 | 0.003 | 4.00E-16 |
| rs11733695 | 4 | 100122916 | G | A | 0.993 | 0.081 | 0.015 | 3.90E-08 |
| rs17287714 | 4 | 159871166 | T | C | 0.882 | 0.022 | 0.004 | 3.70E-08 |
| rs12189146 | 5 | 35239886 | G | A | 0.95 | 0.032 | 0.006 | 1.80E-08 |
| rs40270 | 5 | 55804552 | C | A | 0.773 | 0.019 | 0.003 | 4.00E-10 |
| rs34341 | 5 | 74934009 | T | A | 0.576 | 0.015 | 0.003 | 2.90E-09 |
| rs1119208 | 5 | 76488613 | C | T | 0.649 | 0.017 | 0.003 | 1.20E-11 |
| rs168189 | 5 | 77974427 | T | C | 0.28 | 0.02 | 0.003 | 5.40E-13 |
| rs12658172 | 5 | 124205385 | G | C | 0.842 | 0.03 | 0.004 | 1.80E-18 |
| rs73350117 | 5 | 127877729 | A | C | 0.828 | 0.02 | 0.003 | 4.80E-10 |
| rs1432679 | 5 | 158244083 | C | T | 0.446 | 0.013 | 0.003 | 4.70E-08 |
| rs13153019 | 5 | 176782218 | C | T | 0.249 | 0.018 | 0.003 | 2.30E-09 |
| rs9379084 | 6 | 7231843 | A | G | 0.116 | 0.022 | 0.004 | 2.90E-08 |
| rs4712976 | 6 | 25842203 | C | T | 0.732 | 0.019 | 0.003 | 3.30E-11 |
| rs28641793 | 6 | 31974355 | A | C | 0.012 | 0.084 | 0.012 | 6.20E-14 |
| rs199746610 | 6 | 34364965 | C | A | 0.967 | 0.041 | 0.007 | 2.40E-09 |
| rs62396733 | 6 | 41679691 | T | C | 0.137 | 0.021 | 0.004 | 5.70E-09 |
| rs1214759 | 6 | 43352980 | G | A | 0.679 | 0.018 | 0.003 | 2.80E-12 |
| rs2397112 | 6 | 52684333 | A | G | 0.574 | 0.016 | 0.003 | 4.00E-10 |
| rs386705174 | 6 | 119171422 | GT | G | 0.779 | 0.039 | 0.003 | 1.70E-39 |
| rs77491205 | 6 | 127197741 | G | GTC | 0.618 | 0.02 | 0.003 | 1.40E-12 |
| 6:130386212_GGAGA_G | 6 | 130386212 | G | GGAGA | 0.674 | 0.017 | 0.003 | 3.60E-09 |
| rs9399469 | 6 | 144318529 | A | T | 0.622 | 0.02 | 0.003 | 2.60E-14 |
| rs4869893 | 6 | 157117322 | C | A | 0.282 | 0.016 | 0.003 | 1.10E-08 |
| rs381194 | 6 | 160712194 | G | A | 0.528 | 0.013 | 0.003 | 5.00E-08 |
| rs36019311 | 7 | 73051990 | GTA | G | 0.13 | 0.029 | 0.004 | 3.10E-14 |
| rs17853284 | 7 | 75610876 | C | T | 0.995 | 0.17 | 0.019 | 1.70E-21 |
| rs764029425 | 7 | 97984321 | T | TG | 0.184 | 0.036 | 0.003 | 9.60E-30 |
| rs45446698 | 7 | 99332948 | T | G | 0.958 | 0.161 | 0.006 | 5.10E-148 |
| rs7780066 | 7 | 137801915 | A | G | 0.793 | 0.023 | 0.003 | 3.10E-13 |
| rs9987289 | 8 | 9183358 | A | G | 0.092 | 0.034 | 0.004 | 5.50E-15 |
| rs199787521 | 8 | 23400588 | TTTA | T | 0.24 | 0.017 | 0.003 | 6.90E-10 |
| 8:25248334_GT_G | 8 | 25248334 | GT | G | 0.366 | 0.014 | 0.003 | 4.20E-08 |
| rs191780890 | 8 | 49275276 | G | A | 0.012 | 0.066 | 0.012 | 1.70E-08 |
| rs10504255 | 8 | 59398461 | G | A | 0.337 | 0.017 | 0.003 | 6.10E-10 |
| 8:81418150_ATTTT_A | 8 | 81418150 | ATTTT | A | 0.738 | 0.023 | 0.003 | 1.20E-16 |
| rs113347955 | 8 | 97136852 | G | A | 0.976 | 0.045 | 0.008 | 1.20E-08 |
| rs35783704 | 8 | 105966258 | A | G | 0.101 | 0.033 | 0.004 | 4.70E-16 |
| rs11784903 | 8 | 116979531 | C | T | 0.526 | 0.014 | 0.003 | 3.60E-08 |
| rs12543598 | 8 | 143955318 | T | G | 0.446 | 0.02 | 0.003 | 6.60E-15 |
| rs12683780 | 9 | 16252807 | A | C | 0.669 | 0.018 | 0.003 | 4.70E-12 |
| rs10757893 | 9 | 29599001 | A | G | 0.575 | 0.014 | 0.003 | 9.30E-09 |
| rs61237993 | 9 | 34130435 | A | G | 0.128 | 0.029 | 0.004 | 1.60E-15 |
| rs199816133 | 9 | 86381318 | C | CA | 0.744 | 0.029 | 0.003 | 1.10E-23 |
| 9:114826326_TG_T | 9 | 114826326 | TG | T | 0.793 | 0.022 | 0.003 | 7.00E-13 |
| rs61856128 | 10 | 5063382 | C | A | 0.704 | 0.035 | 0.003 | 1.40E-36 |
| rs1171617 | 10 | 61467182 | T | G | 0.767 | 0.03 | 0.003 | 3.60E-23 |
| 10:65224458_CA_C | 10 | 65224458 | CA | C | 0.479 | 0.032 | 0.003 | 2.50E-36 |
| rs7089122 | 10 | 93647412 | T | C | 0.178 | 0.024 | 0.003 | 1.70E-14 |
| rs9527 | 10 | 104623578 | C | T | 0.758 | 0.023 | 0.003 | 5.90E-15 |
| rs7078330 | 10 | 122834163 | T | C | 0.088 | 0.031 | 0.004 | 5.70E-13 |
| rs6486122 | 11 | 13361524 | T | C | 0.69 | 0.017 | 0.003 | 1.70E-09 |
| rs11031005 | 11 | 30226356 | C | T | 0.144 | 0.023 | 0.004 | 1.50E-10 |
| rs113172275 | 11 | 62905115 | C | T | 0.066 | 0.049 | 0.005 | 1.40E-22 |
| rs35008345 | 11 | 64323613 | C | T | 0.995 | 0.131 | 0.017 | 9.10E-14 |
| rs3814707 | 11 | 65560785 | G | A | 0.758 | 0.02 | 0.003 | 5.50E-11 |
| rs746258907 | 11 | 68095711 | AG | A | 0.472 | 0.016 | 0.003 | 7.70E-10 |
| rs171021 | 11 | 72317557 | C | T | 0.703 | 0.018 | 0.003 | 1.20E-10 |
| rs11235688 | 11 | 72947934 | G | A | 0.583 | 0.016 | 0.003 | 7.00E-12 |
| rs850294 | 11 | 123437669 | T | C | 0.114 | 0.034 | 0.004 | 5.20E-18 |
| rs56196860 | 12 | 2908330 | C | A | 0.969 | 0.048 | 0.007 | 1.40E-10 |
| rs76895963 | 12 | 4384844 | T | G | 0.979 | 0.088 | 0.01 | 5.50E-21 |
| rs4149056 | 12 | 21331549 | C | T | 0.151 | 0.043 | 0.004 | 3.00E-35 |
| rs11047261 | 12 | 24257228 | G | A | 0.041 | 0.037 | 0.006 | 6.40E-09 |
| 12:25412725_AATTATTGTGT_A | 12 | 25412725 | A | AATTATTGTGT | 0.071 | 0.028 | 0.005 | 7.70E-09 |
| rs56205943 | 12 | 57679414 | G | A | 0.759 | 0.021 | 0.003 | 3.90E-13 |
| rs6538437 | 12 | 93989735 | T | A | 0.289 | 0.016 | 0.003 | 3.10E-09 |
| rs7301634 | 12 | 98901200 | A | G | 0.81 | 0.019 | 0.003 | 2.80E-09 |
| rs61755050 | 12 | 100926308 | C | T | 0.006 | 0.091 | 0.016 | 7.80E-09 |
| rs10778215 | 12 | 103537266 | A | T | 0.472 | 0.024 | 0.003 | 2.60E-22 |
| rs7314285 | 12 | 111522026 | T | G | 0.932 | 0.038 | 0.005 | 9.80E-14 |
| rs7139079 | 12 | 121415293 | G | A | 0.407 | 0.022 | 0.003 | 1.10E-16 |
| rs2954111 | 12 | 122134415 | C | T | 0.361 | 0.017 | 0.003 | 3.10E-10 |
| rs629042 | 13 | 22318506 | C | G | 0.607 | 0.028 | 0.003 | 7.40E-29 |
| 13:22693754_TC_T | 13 | 22693754 | T | TC | 0.191 | 0.019 | 0.003 | 1.00E-09 |
| rs17245822 | 13 | 73131694 | C | A | 0.373 | 0.014 | 0.003 | 3.60E-08 |
| rs17128091 | 14 | 23714682 | G | C | 0.744 | 0.016 | 0.003 | 6.70E-09 |
| rs11621792 | 14 | 24871926 | T | C | 0.452 | 0.025 | 0.003 | 1.40E-22 |
| rs112635299 | 14 | 94838142 | G | T | 0.979 | 0.102 | 0.009 | 1.10E-31 |
| rs17580 | 14 | 94847262 | T | A | 0.952 | 0.04 | 0.006 | 6.90E-12 |
| 14:98567544_CAA_C | 14 | 98567544 | C | CAA | 0.291 | 0.018 | 0.003 | 4.80E-11 |
| rs7183977 | 15 | 40377092 | C | T | 0.348 | 0.029 | 0.003 | 7.80E-27 |
| rs10851395 | 15 | 40718534 | C | T | 0.524 | 0.019 | 0.003 | 1.10E-13 |
| 15:43674414_AAAAAAG_A | 15 | 43674414 | AAAAAAG | A | 0.915 | 0.024 | 0.005 | 1.90E-08 |
| 15:53094375_TTTTG_T | 15 | 53094375 | T | TTTTG | 0.114 | 0.03 | 0.004 | 4.40E-14 |
| rs149624078 | 15 | 53728710 | T | C | 0.014 | 0.079 | 0.011 | 3.10E-12 |
| rs71447853 | 15 | 65965207 | A | AT | 0.581 | 0.015 | 0.003 | 2.30E-09 |
| rs111475133 | 15 | 75810807 | T | TA | 0.803 | 0.026 | 0.003 | 3.20E-15 |
| rs62025141 | 15 | 79850183 | A | G | 0.855 | 0.026 | 0.004 | 1.20E-13 |
| rs71397837 | 15 | 85563561 | G | A | 0.83 | 0.02 | 0.003 | 9.60E-10 |
| rs56332871 | 15 | 96714816 | C | A | 0.728 | 0.03 | 0.003 | 9.70E-27 |
| rs879619 | 16 | 4015046 | G | A | 0.864 | 0.021 | 0.004 | 1.40E-09 |
| rs388430 | 16 | 4135562 | C | T | 0.683 | 0.017 | 0.003 | 2.60E-10 |
| rs8046391 | 16 | 30836648 | C | G | 0.272 | 0.017 | 0.003 | 8.80E-10 |
| rs58072681 | 16 | 81590541 | C | T | 0.07 | 0.061 | 0.005 | 8.30E-36 |
| rs56731455 | 16 | 88068078 | AT | A | 0.567 | 0.015 | 0.003 | 9.40E-09 |
| rs11078597 | 17 | 1618363 | T | C | 0.814 | 0.019 | 0.003 | 5.00E-09 |
| rs6258 | 17 | 7534678 | T | C | 0.007 | 0.555 | 0.015 | 7.00E-286 |
| rs727428 | 17 | 7537792 | T | C | 0.444 | 0.095 | 0.003 | 0.00E+00 |
| rs35596561 | 17 | 45632713 | CT | C | 0.495 | 0.025 | 0.003 | 8.30E-23 |
| rs11653686 | 17 | 47362991 | C | T | 0.913 | 0.066 | 0.004 | 8.30E-48 |
| rs4793788 | 17 | 53381796 | C | A | 0.364 | 0.016 | 0.003 | 3.80E-10 |
| 17:63685553_CA_C | 17 | 63685553 | C | CA | 0.332 | 0.017 | 0.003 | 1.10E-09 |
| rs8178824 | 17 | 64224775 | T | C | 0.03 | 0.058 | 0.007 | 5.10E-15 |
| rs34931250 | 17 | 66879927 | C | T | 0.939 | 0.04 | 0.005 | 3.10E-15 |
| rs2587507 | 17 | 77790135 | C | T | 0.507 | 0.014 | 0.003 | 9.60E-09 |
| 18:3812373_AT_A | 18 | 3812373 | A | AT | 0.688 | 0.019 | 0.003 | 2.90E-11 |
| rs117327231 | 18 | 71916636 | A | C | 0.023 | 0.097 | 0.008 | 2.80E-31 |
| rs7239564 | 18 | 71967031 | C | T | 0.856 | 0.031 | 0.004 | 5.00E-18 |
| rs112933999 | 19 | 1825480 | AT | A | 0.539 | 0.015 | 0.003 | 4.90E-10 |
| rs1640272 | 19 | 2800192 | A | T | 0.713 | 0.021 | 0.003 | 3.70E-14 |
| rs7248104 | 19 | 7224431 | G | A | 0.584 | 0.016 | 0.003 | 4.50E-10 |
| rs8111359 | 19 | 10471462 | C | T | 0.905 | 0.034 | 0.004 | 8.40E-16 |
| rs202200760 | 19 | 17346854 | G | C | 0.961 | 0.063 | 0.007 | 2.30E-19 |
| rs1688043 | 19 | 35553341 | C | T | 0.066 | 0.041 | 0.005 | 1.90E-16 |
| rs181255261 | 19 | 42929883 | A | G | 0.006 | 0.116 | 0.017 | 7.80E-12 |
| rs34954997 | 19 | 45417638 | C | CTTCG | 0.764 | 0.017 | 0.003 | 4.90E-09 |
| rs11879227 | 19 | 46193351 | A | G | 0.817 | 0.02 | 0.003 | 1.00E-09 |
| rs34255979 | 19 | 46384830 | C | T | 0.879 | 0.023 | 0.004 | 1.50E-09 |
| rs6129778 | 20 | 39870578 | C | A | 0.81 | 0.02 | 0.003 | 2.90E-09 |
| rs6073431 | 20 | 43040569 | C | T | 0.468 | 0.015 | 0.003 | 3.50E-09 |
| rs6020423 | 20 | 48909667 | C | T | 0.76 | 0.025 | 0.003 | 2.30E-18 |
| rs74652944 | 21 | 33750632 | C | T | 0.008 | 0.106 | 0.014 | 1.80E-13 |
| rs62223042 | 21 | 40683740 | G | A | 0.375 | 0.015 | 0.003 | 1.10E-09 |
| rs738409 | 22 | 44324727 | C | G | 0.784 | 0.017 | 0.003 | 1.70E-08 |
| rs6008259 | 22 | 46633782 | G | A | 0.821 | 0.021 | 0.003 | 5.30E-11 |
| rs7291444 | 22 | 46656246 | T | G | 0.852 | 0.021 | 0.004 | 9.80E-10 |
| rs182375344 | 23 | 109736206 | T | C | 0.635 | 0.017 | 0.003 | 5.90E-10 |
| rs67952556 | 23 | 135869176 | T | G | 0.524 | 0.015 | 0.003 | 9.00E-09 |
| rs5970442 | 23 | 152590165 | C | G | 0.496 | 0.027 | 0.003 | 8.00E-26 |

**Table S2** – Genome-wide significant single nucleotide polymorphisms (SNP) used as instrumental variants for total testosterone in females..

| **SNP** | **Variant chromosome** | **Variant position** | **Effect allele** | **Non-effect allele** | **Effect allele frequency** | **Beta** | **Standard error** | **P-value** |
| --- | --- | --- | --- | --- | --- | --- | --- | --- |
| rs571084788 | 1 | 7843711 | T | C | 0.807 | 0.024 | 0.004 | 1.40E-11 |
| rs10799713 | 1 | 22102328 | G | C | 0.212 | 0.027 | 0.003 | 9.20E-16 |
| rs7530117 | 1 | 31263360 | T | C | 0.369 | 0.018 | 0.003 | 1.30E-09 |
| rs4453027 | 1 | 41453453 | G | T | 0.575 | 0.023 | 0.003 | 1.40E-15 |
| rs1278526 | 1 | 50891213 | A | C | 0.524 | 0.019 | 0.003 | 2.10E-12 |
| rs7529520 | 1 | 57016477 | C | G | 0.486 | 0.018 | 0.003 | 6.20E-12 |
| rs505237 | 1 | 66738623 | G | A | 0.616 | 0.016 | 0.003 | 3.70E-08 |
| rs111882448 | 1 | 68846740 | CA | C | 0.351 | 0.017 | 0.003 | 3.80E-09 |
| rs4294422 | 1 | 93104655 | G | A | 0.359 | 0.019 | 0.003 | 6.80E-11 |
| rs6684361 | 1 | 1.02E+08 | C | T | 0.307 | 0.076 | 0.003 | 7.40E-141 |
| rs3032555 | 1 | 1.08E+08 | T | TCAG | 0.342 | 0.018 | 0.003 | 4.40E-09 |
| rs7519368 | 1 | 1.13E+08 | T | A | 0.727 | 0.033 | 0.003 | 3.10E-25 |
| rs72693130 | 1 | 1.2E+08 | A | G | 0.063 | 0.031 | 0.006 | 2.90E-08 |
| 1:150186390_AG_A | 1 | 1.5E+08 | A | AG | 0.422 | 0.017 | 0.003 | 1.70E-09 |
| rs58175144 | 1 | 1.51E+08 | CA | C | 0.303 | 0.021 | 0.003 | 3.50E-12 |
| rs1870940 | 1 | 1.55E+08 | G | A | 0.727 | 0.025 | 0.003 | 4.90E-17 |
| rs76830943 | 1 | 1.56E+08 | T | C | 0.833 | 0.024 | 0.004 | 3.00E-10 |
| rs12564492 | 1 | 1.68E+08 | A | G | 0.703 | 0.02 | 0.003 | 3.80E-11 |
| rs569421885 | 1 | 1.79E+08 | CA | C | 0.245 | 0.055 | 0.003 | 1.10E-62 |
| rs2062479 | 1 | 1.98E+08 | C | G | 0.524 | 0.016 | 0.003 | 1.80E-08 |
| rs17043570 | 1 | 2.17E+08 | T | C | 0.159 | 0.025 | 0.004 | 4.10E-12 |
| rs12078363 | 1 | 2.19E+08 | T | C | 0.681 | 0.028 | 0.003 | 1.00E-21 |
| rs10910476 | 1 | 2.35E+08 | C | T | 0.444 | 0.017 | 0.003 | 1.10E-09 |
| rs34269793 | 2 | 12555164 | C | T | 0.053 | 0.064 | 0.006 | 2.30E-25 |
| rs35397738 | 2 | 17766649 | TA | T | 0.348 | 0.02 | 0.003 | 2.40E-12 |
| rs3771243 | 2 | 20412227 | A | G | 0.39 | 0.029 | 0.003 | 1.40E-26 |
| rs11892043 | 2 | 24990082 | A | G | 0.728 | 0.022 | 0.003 | 2.40E-11 |
| rs1260326 | 2 | 27730940 | C | T | 0.607 | 0.037 | 0.003 | 4.70E-39 |
| 2:32504650_AT_A | 2 | 32504650 | A | AT | 0.69 | 0.017 | 0.003 | 4.30E-08 |
| rs2374456 | 2 | 43271621 | G | C | 0.584 | 0.024 | 0.003 | 9.30E-17 |
| rs7575635 | 2 | 43515427 | C | T | 0.806 | 0.038 | 0.004 | 1.40E-27 |
| rs3136354 | 2 | 48031372 | C | T | 0.501 | 0.024 | 0.003 | 3.50E-18 |
| rs11125180 | 2 | 48942282 | A | T | 0.909 | 0.033 | 0.005 | 5.40E-12 |
| rs7573187 | 2 | 62536671 | A | T | 0.535 | 0.019 | 0.003 | 1.30E-11 |
| rs1009360 | 2 | 65276049 | C | T | 0.416 | 0.018 | 0.003 | 1.50E-09 |
| rs10865479 | 2 | 86095432 | T | C | 0.715 | 0.022 | 0.003 | 2.40E-13 |
| rs113247979 | 2 | 1.03E+08 | T | C | 0.993 | 0.14 | 0.017 | 6.60E-17 |
| rs590097 | 2 | 1.12E+08 | G | T | 0.646 | 0.061 | 0.003 | 1.60E-100 |
| rs62162863 | 2 | 1.12E+08 | G | T | 0.577 | 0.023 | 0.003 | 1.10E-13 |
| rs10168169 | 2 | 1.12E+08 | T | C | 0.812 | 0.033 | 0.004 | 5.10E-20 |
| 2:135432074_CGCGCGCTACCATATCT_C | 2 | 1.35E+08 | C | CGCGCGCTACCATATCT | 0.615 | 0.017 | 0.003 | 1.30E-10 |
| rs59741822 | 2 | 1.37E+08 | G | A | 0.923 | 0.033 | 0.005 | 7.00E-12 |
| rs58723250 | 2 | 1.78E+08 | T | C | 0.2 | 0.035 | 0.004 | 2.20E-23 |
| rs36088520 | 2 | 1.98E+08 | T | C | 0.104 | 0.028 | 0.005 | 5.70E-09 |
| rs873779 | 2 | 2.09E+08 | C | T | 0.372 | 0.016 | 0.003 | 1.80E-09 |
| rs2011425 | 2 | 2.35E+08 | T | G | 0.921 | 0.033 | 0.005 | 6.60E-11 |
| rs10687319 | 2 | 2.42E+08 | AAG | A | 0.48 | 0.015 | 0.003 | 1.90E-08 |
| rs7618363 | 3 | 10545125 | C | G | 0.841 | 0.037 | 0.004 | 1.20E-21 |
| rs62231822 | 3 | 14423060 | C | T | 0.902 | 0.049 | 0.005 | 5.90E-26 |
| 3:20085213_CTT_C | 3 | 20085213 | C | CTT | 0.187 | 0.021 | 0.004 | 5.40E-09 |
| rs534141419 | 3 | 23478055 | A | AT | 0.625 | 0.018 | 0.003 | 4.50E-10 |
| 3:27554392_CT_C | 3 | 27554392 | C | CT | 0.027 | 0.054 | 0.009 | 1.80E-10 |
| rs17201704 | 3 | 41105589 | T | C | 0.859 | 0.043 | 0.004 | 2.90E-28 |
| rs4067 | 3 | 51738256 | G | A | 0.855 | 0.024 | 0.004 | 1.70E-10 |
| rs13094915 | 3 | 52507719 | G | C | 0.632 | 0.016 | 0.003 | 2.00E-09 |
| rs9832502 | 3 | 72398979 | A | G | 0.249 | 0.017 | 0.003 | 3.80E-09 |
| rs167096 | 3 | 73874699 | T | G | 0.26 | 0.017 | 0.003 | 4.30E-10 |
| 3:119786083_CA_C | 3 | 1.2E+08 | CA | C | 0.617 | 0.017 | 0.003 | 9.00E-09 |
| rs71624037 | 3 | 1.34E+08 | G | GT | 0.926 | 0.036 | 0.005 | 5.70E-11 |
| rs7633673 | 3 | 1.52E+08 | G | A | 0.594 | 0.026 | 0.003 | 7.50E-20 |
| 3:156851624_CA_C | 3 | 1.57E+08 | C | CA | 0.344 | 0.019 | 0.003 | 4.20E-11 |
| rs9850919 | 3 | 1.69E+08 | C | T | 0.405 | 0.02 | 0.003 | 2.80E-13 |
| rs5855544 | 3 | 1.96E+08 | T | TG | 0.429 | 0.033 | 0.003 | 1.30E-32 |
| rs77822621 | 4 | 1008212 | T | C | 0.042 | 0.048 | 0.007 | 9.30E-14 |
| rs3849653 | 4 | 53860084 | A | T | 0.541 | 0.019 | 0.003 | 6.60E-12 |
| rs200457494 | 4 | 55087241 | CA | C | 0.212 | 0.025 | 0.004 | 2.10E-12 |
| 4:57746479_TA_T | 4 | 57746479 | TA | T | 0.532 | 0.02 | 0.003 | 1.90E-12 |
| rs4632729 | 4 | 69946004 | A | G | 0.545 | 0.028 | 0.003 | 2.40E-22 |
| rs60117481 | 4 | 77420598 | G | GTATAAC | 0.354 | 0.019 | 0.003 | 6.70E-11 |
| rs1229984 | 4 | 1E+08 | C | T | 0.976 | 0.049 | 0.009 | 1.20E-08 |
| rs4586943 | 4 | 1.02E+08 | A | C | 0.282 | 0.018 | 0.003 | 6.90E-09 |
| rs28484580 | 4 | 1.03E+08 | G | A | 0.224 | 0.021 | 0.004 | 1.70E-08 |
| rs2903385 | 4 | 1.06E+08 | A | G | 0.486 | 0.025 | 0.003 | 1.60E-18 |
| rs4245930 | 4 | 1.09E+08 | G | A | 0.368 | 0.025 | 0.003 | 1.10E-19 |
| rs749512116 | 4 | 1.14E+08 | CAGAT | C | 0.816 | 0.033 | 0.004 | 6.90E-20 |
| rs371162363 | 4 | 1.78E+08 | G | A | 0.904 | 0.039 | 0.005 | 5.60E-15 |
| rs112694713 | 5 | 35247932 | A | G | 0.986 | 0.107 | 0.012 | 2.40E-19 |
| rs9687846 | 5 | 55861894 | G | A | 0.8 | 0.026 | 0.004 | 3.60E-12 |
| rs4431325 | 5 | 76461706 | T | C | 0.059 | 0.05 | 0.006 | 8.50E-18 |
| rs1119208 | 5 | 76488613 | C | T | 0.648 | 0.03 | 0.003 | 9.80E-26 |
| rs784420 | 5 | 77987524 | G | A | 0.289 | 0.039 | 0.003 | 1.20E-39 |
| rs11948639 | 5 | 1.23E+08 | T | C | 0.434 | 0.017 | 0.003 | 3.20E-09 |
| rs12658172 | 5 | 1.24E+08 | G | C | 0.842 | 0.052 | 0.004 | 5.80E-41 |
| rs13184921 | 5 | 1.28E+08 | T | C | 0.753 | 0.025 | 0.003 | 7.60E-16 |
| rs3776299 | 5 | 1.43E+08 | G | A | 0.549 | 0.017 | 0.003 | 3.60E-10 |
| rs34632394 | 5 | 1.43E+08 | CAT | C | 0.538 | 0.017 | 0.003 | 3.10E-10 |
| rs33959428 | 5 | 1.58E+08 | G | GA | 0.328 | 0.018 | 0.003 | 8.30E-09 |
| rs13153019 | 5 | 1.77E+08 | C | T | 0.249 | 0.024 | 0.003 | 1.50E-14 |
| rs75217853 | 6 | 1377048 | A | G | 0.098 | 0.035 | 0.005 | 1.30E-14 |
| rs267190 | 6 | 7842121 | G | T | 0.578 | 0.015 | 0.003 | 3.70E-08 |
| rs6904345 | 6 | 24697424 | T | C | 0.634 | 0.017 | 0.003 | 1.90E-08 |
| rs487624 | 6 | 25879539 | C | A | 0.568 | 0.019 | 0.003 | 1.40E-12 |
| rs2517582 | 6 | 30808762 | T | C | 0.384 | 0.028 | 0.003 | 1.60E-18 |
| rs184265581 | 6 | 31900754 | C | G | 0.012 | 0.132 | 0.013 | 6.40E-27 |
| 6:34753277_GA_G | 6 | 34753277 | G | GA | 0.239 | 0.018 | 0.003 | 2.30E-09 |
| rs1214761 | 6 | 43354431 | G | A | 0.679 | 0.031 | 0.003 | 6.20E-26 |
| rs2608652 | 6 | 52642768 | T | C | 0.525 | 0.015 | 0.003 | 1.30E-08 |
| rs1032388 | 6 | 1.19E+08 | C | T | 0.779 | 0.054 | 0.003 | 2.50E-61 |
| rs779008104 | 6 | 1.27E+08 | AAAACAGATAC | A | 0.566 | 0.024 | 0.003 | 1.90E-18 |
| rs577721086 | 6 | 1.27E+08 | T | C | 0.95 | 0.055 | 0.007 | 5.80E-17 |
| 6:144301525_GT_G | 6 | 1.44E+08 | G | GT | 0.66 | 0.028 | 0.003 | 1.90E-19 |
| rs2473140 | 6 | 1.45E+08 | C | T | 0.09 | 0.029 | 0.005 | 7.20E-10 |
| rs287884 | 6 | 1.57E+08 | A | T | 0.444 | 0.019 | 0.003 | 2.10E-11 |
| rs9457466 | 6 | 1.59E+08 | A | T | 0.363 | 0.017 | 0.003 | 1.50E-09 |
| rs2344744 | 6 | 1.71E+08 | G | T | 0.415 | 0.018 | 0.003 | 1.30E-09 |
| rs28612846 | 7 | 25950880 | G | A | 0.743 | 0.016 | 0.003 | 4.70E-08 |
| rs11427441 | 7 | 47522085 | CA | C | 0.654 | 0.02 | 0.003 | 1.30E-11 |
| rs6460528 | 7 | 69218337 | T | C | 0.454 | 0.015 | 0.003 | 4.30E-08 |
| rs13229619 | 7 | 73030175 | A | G | 0.129 | 0.054 | 0.004 | 8.30E-39 |
| rs17853284 | 7 | 75610876 | C | T | 0.995 | 0.304 | 0.021 | 5.30E-49 |
| 7:98188671_CCTT_C | 7 | 98188671 | CCTT | C | 0.995 | 0.164 | 0.022 | 1.20E-13 |
| rs45446698 | 7 | 99332948 | T | G | 0.958 | 0.371 | 0.007 | 1.5E-635 |
| rs62621812 | 7 | 1.27E+08 | A | G | 0.02 | 0.053 | 0.01 | 1.30E-08 |
| rs1872930 | 7 | 1.38E+08 | T | C | 0.793 | 0.051 | 0.003 | 1.70E-54 |
| rs9638084 | 7 | 1.56E+08 | G | A | 0.604 | 0.021 | 0.003 | 1.30E-13 |
| rs17362923 | 8 | 5546824 | G | C | 0.189 | 0.023 | 0.004 | 3.00E-11 |
| rs6997799 | 8 | 10606421 | C | A | 0.234 | 0.024 | 0.003 | 5.80E-13 |
| rs56109436 | 8 | 11543663 | G | C | 0.099 | 0.026 | 0.005 | 6.00E-09 |
| rs199787521 | 8 | 23400588 | TTTA | T | 0.24 | 0.029 | 0.003 | 8.50E-20 |
| rs17053931 | 8 | 25417528 | A | G | 0.199 | 0.021 | 0.004 | 4.50E-09 |
| rs11782259 | 8 | 37532984 | A | G | 0.911 | 0.031 | 0.005 | 1.10E-10 |
| rs11778724 | 8 | 49549726 | C | A | 0.013 | 0.07 | 0.013 | 1.50E-08 |
| rs10108398 | 8 | 59440824 | G | A | 0.279 | 0.018 | 0.003 | 1.10E-08 |
| rs13269725 | 8 | 72459889 | A | G | 0.921 | 0.03 | 0.005 | 1.80E-08 |
| rs1660322 | 8 | 1.01E+08 | T | C | 0.687 | 0.023 | 0.003 | 4.50E-15 |
| rs11774829 | 8 | 1.06E+08 | A | T | 0.101 | 0.056 | 0.005 | 5.80E-32 |
| rs4736359 | 8 | 1.44E+08 | T | G | 0.443 | 0.037 | 0.003 | 6.20E-40 |
| rs12683780 | 9 | 16252807 | A | C | 0.668 | 0.031 | 0.003 | 7.50E-26 |
| rs4961485 | 9 | 16360889 | T | C | 0.934 | 0.039 | 0.006 | 8.90E-14 |
| rs61237993 | 9 | 34130435 | A | G | 0.128 | 0.04 | 0.004 | 6.90E-23 |
| rs10821415 | 9 | 97713459 | C | A | 0.579 | 0.015 | 0.003 | 1.60E-08 |
| rs1547308 | 9 | 1.15E+08 | C | T | 0.8 | 0.032 | 0.004 | 9.20E-21 |
| rs10817260 | 9 | 1.15E+08 | C | T | 0.81 | 0.043 | 0.004 | 9.30E-35 |
| 9:127663551_AT_A | 9 | 1.28E+08 | AT | A | 0.582 | 0.018 | 0.003 | 2.60E-10 |
| rs776074878 | 9 | 1.3E+08 | CA | C | 0.691 | 0.021 | 0.003 | 4.40E-10 |
| rs494242 | 9 | 1.36E+08 | C | T | 0.659 | 0.017 | 0.003 | 1.70E-08 |
| rs35249079 | 10 | 1037737 | T | G | 0.531 | 0.022 | 0.003 | 1.40E-15 |
| 10:5034014_ATTAC_A | 10 | 5034014 | ATTAC | A | 0.994 | 0.183 | 0.019 | 2.40E-21 |
| rs36032941 | 10 | 5062752 | C | A | 0.704 | 0.063 | 0.003 | 3.00E-94 |
| rs1171617 | 10 | 61467182 | T | G | 0.767 | 0.052 | 0.003 | 5.50E-57 |
| rs10740131 | 10 | 65271488 | T | A | 0.473 | 0.028 | 0.003 | 2.10E-25 |
| rs674486 | 10 | 69819917 | C | T | 0.35 | 0.018 | 0.003 | 1.10E-08 |
| rs35199395 | 10 | 70983936 | C | G | 0.694 | 0.017 | 0.003 | 7.40E-09 |
| rs2147419 | 10 | 90759916 | T | G | 0.718 | 0.018 | 0.003 | 1.60E-09 |
| rs745932882 | 10 | 94441992 | AG | A | 0.546 | 0.027 | 0.003 | 6.70E-22 |
| rs111328885 | 10 | 96823812 | C | A | 0.878 | 0.033 | 0.004 | 1.50E-14 |
| rs11191421 | 10 | 1.05E+08 | C | G | 0.758 | 0.037 | 0.003 | 4.20E-31 |
| rs11191801 | 10 | 1.06E+08 | A | C | 0.708 | 0.023 | 0.003 | 8.70E-16 |
| 10:126834851_AT_A | 10 | 1.27E+08 | A | AT | 0.497 | 0.016 | 0.003 | 1.00E-08 |
| rs440150 | 11 | 2936657 | G | A | 0.093 | 0.026 | 0.005 | 2.20E-08 |
| rs11024458 | 11 | 18082483 | A | G | 0.728 | 0.019 | 0.003 | 2.30E-10 |
| rs10501081 | 11 | 27236061 | G | C | 0.138 | 0.022 | 0.004 | 1.30E-08 |
| rs11031005 | 11 | 30226356 | C | T | 0.143 | 0.033 | 0.004 | 7.20E-17 |
| rs1939769 | 11 | 62914202 | A | G | 0.066 | 0.083 | 0.006 | 1.30E-51 |
| rs35008345 | 11 | 64323613 | C | T | 0.995 | 0.183 | 0.019 | 1.20E-20 |
| rs312023 | 11 | 68097049 | G | A | 0.472 | 0.028 | 0.003 | 3.00E-24 |
| rs171021 | 11 | 72317557 | C | T | 0.703 | 0.038 | 0.003 | 4.20E-35 |
| rs11235688 | 11 | 72947934 | G | A | 0.583 | 0.022 | 0.003 | 3.70E-16 |
| 11:110176972_CT_C | 11 | 1.1E+08 | CT | C | 0.528 | 0.017 | 0.003 | 8.70E-10 |
| rs75848431 | 11 | 1.23E+08 | T | C | 0.152 | 0.032 | 0.004 | 4.20E-15 |
| 11:123438708_TGAG_T | 11 | 1.23E+08 | T | TGAG | 0.112 | 0.048 | 0.005 | 4.90E-28 |
| rs76299412 | 11 | 1.28E+08 | A | G | 0.153 | 0.035 | 0.004 | 2.00E-20 |
| rs56196860 | 12 | 2908330 | C | A | 0.969 | 0.058 | 0.008 | 3.70E-12 |
| rs881613 | 12 | 12738486 | A | G | 0.301 | 0.017 | 0.003 | 3.10E-08 |
| rs4149056 | 12 | 21331549 | C | T | 0.151 | 0.029 | 0.004 | 1.10E-14 |
| rs117913411 | 12 | 48254353 | T | A | 0.966 | 0.048 | 0.008 | 3.30E-10 |
| rs35801460 | 12 | 49180828 | G | A | 0.797 | 0.02 | 0.003 | 6.40E-09 |
| 12:49441189_CT_C | 12 | 49441189 | CT | C | 0.977 | 0.061 | 0.01 | 1.10E-09 |
| rs7977247 | 12 | 1.07E+08 | T | C | 0.578 | 0.018 | 0.003 | 3.90E-11 |
| rs35427 | 12 | 1.16E+08 | T | G | 0.617 | 0.017 | 0.003 | 3.40E-10 |
| rs1169289 | 12 | 1.21E+08 | G | C | 0.44 | 0.019 | 0.003 | 2.50E-11 |
| 12:123639761_TAAATA_T | 12 | 1.24E+08 | TAAATA | T | 0.186 | 0.019 | 0.004 | 2.20E-08 |
| rs837493 | 12 | 1.25E+08 | G | A | 0.452 | 0.016 | 0.003 | 1.10E-08 |
| rs9506725 | 13 | 22314146 | T | C | 0.629 | 0.052 | 0.003 | 4.80E-73 |
| rs9552597 | 13 | 22690127 | A | G | 0.191 | 0.035 | 0.004 | 5.20E-24 |
| rs4943729 | 13 | 32353100 | A | C | 0.518 | 0.015 | 0.003 | 1.10E-08 |
| rs17764067 | 13 | 33728353 | G | A | 0.78 | 0.021 | 0.003 | 5.50E-10 |
| rs17245822 | 13 | 73131694 | C | A | 0.373 | 0.028 | 0.003 | 2.30E-23 |
| rs9599996 | 13 | 73203811 | T | G | 0.599 | 0.017 | 0.003 | 2.10E-10 |
| rs72660136 | 13 | 1.1E+08 | T | C | 0.966 | 0.06 | 0.008 | 4.90E-16 |
| rs7342537 | 14 | 21555063 | G | A | 0.018 | 0.107 | 0.011 | 2.80E-24 |
| rs59397130 | 14 | 64898076 | G | A | 0.026 | 0.056 | 0.009 | 2.30E-10 |
| rs61987429 | 14 | 65606248 | C | T | 0.649 | 0.017 | 0.003 | 9.20E-10 |
| rs1314911 | 14 | 68698230 | A | G | 0.859 | 0.026 | 0.004 | 7.20E-10 |
| rs72731535 | 14 | 69237902 | G | A | 0.822 | 0.025 | 0.004 | 9.10E-12 |
| rs112635299 | 14 | 94838142 | G | T | 0.979 | 0.062 | 0.01 | 2.90E-11 |
| rs12436785 | 14 | 98550490 | C | T | 0.417 | 0.03 | 0.003 | 2.80E-26 |
| rs10147094 | 14 | 99727591 | A | G | 0.614 | 0.022 | 0.003 | 6.90E-16 |
| rs12893790 | 14 | 1.01E+08 | G | C | 0.816 | 0.021 | 0.004 | 1.00E-09 |
| rs77432559 | 14 | 1.07E+08 | G | C | 0.079 | 0.037 | 0.006 | 4.20E-11 |
| rs11638521 | 15 | 40360314 | T | C | 0.347 | 0.064 | 0.003 | 2.60E-110 |
| rs61661087 | 15 | 40720543 | C | T | 0.524 | 0.028 | 0.003 | 1.60E-24 |
| rs34584018 | 15 | 41531518 | G | GT | 0.59 | 0.016 | 0.003 | 1.00E-08 |
| rs72738949 | 15 | 50668999 | T | A | 0.42 | 0.019 | 0.003 | 3.20E-12 |
| rs2113944 | 15 | 60977882 | T | C | 0.223 | 0.027 | 0.003 | 2.70E-15 |
| 15:66049600_TG_T | 15 | 66049600 | TG | T | 0.562 | 0.019 | 0.003 | 2.60E-11 |
| rs12708515 | 15 | 75454879 | G | C | 0.364 | 0.021 | 0.003 | 2.10E-14 |
| rs4464040 | 15 | 79840557 | C | T | 0.85 | 0.045 | 0.004 | 4.40E-32 |
| rs12900736 | 15 | 85558140 | C | T | 0.83 | 0.04 | 0.004 | 1.90E-27 |
| rs2074585 | 15 | 91009484 | A | G | 0.514 | 0.016 | 0.003 | 8.80E-09 |
| rs437115 | 16 | 4156423 | T | C | 0.565 | 0.028 | 0.003 | 4.20E-24 |
| rs8045779 | 16 | 12907061 | T | C | 0.836 | 0.026 | 0.004 | 1.40E-11 |
| rs534645300 | 16 | 30813454 | AT | A | 0.244 | 0.021 | 0.003 | 7.50E-11 |
| rs8044588 | 16 | 67420328 | C | G | 0.089 | 0.028 | 0.005 | 1.50E-09 |
| rs58072681 | 16 | 81590541 | C | T | 0.07 | 0.108 | 0.006 | 7.90E-86 |
| rs187370584 | 17 | 2309972 | A | G | 0.987 | 0.066 | 0.012 | 1.50E-08 |
| rs9898480 | 17 | 6525861 | T | C | 0.569 | 0.018 | 0.003 | 9.80E-12 |
| rs62059839 | 17 | 7533015 | T | C | 0.261 | 0.032 | 0.003 | 9.50E-24 |
| rs2270445 | 17 | 8219478 | G | A | 0.487 | 0.017 | 0.003 | 4.60E-10 |
| rs34557412 | 17 | 16852187 | A | G | 0.993 | 0.107 | 0.017 | 1.80E-10 |
| rs1242518 | 17 | 17387899 | T | C | 0.747 | 0.02 | 0.003 | 4.80E-10 |
| 17:53371413_TA_T | 17 | 53371413 | TA | T | 0.377 | 0.019 | 0.003 | 3.80E-11 |
| rs232159 | 17 | 63708321 | C | T | 0.349 | 0.018 | 0.003 | 1.10E-08 |
| rs34931250 | 17 | 66879927 | C | T | 0.939 | 0.054 | 0.006 | 3.20E-21 |
| rs532213132 | 17 | 81058362 | GC | G | 0.646 | 0.016 | 0.003 | 2.10E-08 |
| rs28421540 | 18 | 3818842 | A | C | 0.714 | 0.031 | 0.003 | 1.90E-24 |
| rs2186945 | 18 | 13921536 | C | T | 0.162 | 0.025 | 0.004 | 2.00E-10 |
| rs34163044 | 18 | 51851616 | A | C | 0.419 | 0.017 | 0.003 | 2.70E-09 |
| rs112367565 | 18 | 71883232 | A | C | 0.046 | 0.049 | 0.007 | 1.30E-13 |
| rs9319895 | 18 | 71896469 | A | G | 0.515 | 0.023 | 0.003 | 2.80E-15 |
| rs117327231 | 18 | 71916636 | A | C | 0.023 | 0.173 | 0.009 | 6.80E-82 |
| rs12977787 | 19 | 1814025 | A | G | 0.541 | 0.015 | 0.003 | 7.20E-09 |
| rs8111359 | 19 | 10471462 | C | T | 0.905 | 0.06 | 0.005 | 1.10E-35 |
| rs4804181 | 19 | 12509536 | C | A | 0.221 | 0.037 | 0.003 | 1.30E-26 |
| rs10686842 | 19 | 18394549 | TAAA | T | 0.632 | 0.028 | 0.003 | 1.20E-22 |
| rs11673591 | 19 | 41985931 | A | T | 0.252 | 0.028 | 0.003 | 5.70E-19 |
| rs138983180 | 19 | 42914668 | A | G | 0.006 | 0.162 | 0.019 | 2.00E-17 |
| rs7256920 | 19 | 46203083 | G | A | 0.515 | 0.019 | 0.003 | 5.50E-11 |
| rs774224295 | 19 | 47715837 | T | TC | 0.681 | 0.019 | 0.003 | 1.40E-10 |
| rs2879910 | 19 | 48335029 | C | T | 0.481 | 0.016 | 0.003 | 1.70E-08 |
| rs75287599 | 19 | 49517140 | T | C | 0.077 | 0.043 | 0.005 | 1.70E-17 |
| rs11697333 | 20 | 31189078 | T | C | 0.674 | 0.019 | 0.003 | 1.80E-10 |
| rs1883711 | 20 | 39179822 | G | C | 0.968 | 0.05 | 0.008 | 2.00E-09 |
| rs6020423 | 20 | 48909667 | C | T | 0.76 | 0.04 | 0.003 | 1.10E-36 |
| rs6127099 | 20 | 52731402 | A | T | 0.721 | 0.018 | 0.003 | 1.10E-08 |
| rs6100174 | 20 | 57280575 | C | T | 0.625 | 0.02 | 0.003 | 1.70E-13 |
| 20:60691366_CT_C | 20 | 60691366 | C | CT | 0.775 | 0.019 | 0.004 | 3.40E-08 |
| rs8126001 | 20 | 62711459 | T | C | 0.49 | 0.02 | 0.003 | 6.30E-13 |
| rs2824138 | 21 | 18294462 | C | T | 0.182 | 0.026 | 0.004 | 1.70E-13 |
| rs74652944 | 21 | 33750632 | C | T | 0.008 | 0.183 | 0.016 | 3.70E-31 |
| 21:40645556_AAGGCTGAGATGGGGGGAC_A | 21 | 40645556 | A | AAGGCTGAGATGGGGGGAC | 0.211 | 0.021 | 0.004 | 3.40E-10 |
| 21:43349613_GC_G | 21 | 43349613 | G | GC | 0.087 | 0.033 | 0.005 | 9.10E-12 |
| rs12185851 | 21 | 43372219 | C | T | 0.231 | 0.021 | 0.003 | 3.20E-11 |
| rs9611014 | 22 | 22453513 | C | T | 0.784 | 0.023 | 0.003 | 3.10E-12 |
| rs8184986 | 22 | 29127224 | A | T | 0.866 | 0.023 | 0.004 | 2.40E-08 |
| rs12628709 | 22 | 29647854 | G | A | 0.109 | 0.026 | 0.005 | 1.80E-09 |
| rs4820829 | 22 | 30524248 | C | T | 0.977 | 0.058 | 0.009 | 4.10E-10 |
| 22:31626042_CTT_C | 22 | 31626042 | CTT | C | 0.421 | 0.016 | 0.003 | 4.50E-08 |
| rs5751229 | 22 | 42545221 | A | G | 0.228 | 0.025 | 0.003 | 6.10E-14 |
| rs6008259 | 22 | 46633782 | G | A | 0.821 | 0.034 | 0.004 | 2.20E-22 |
| rs7291444 | 22 | 46656246 | T | G | 0.852 | 0.033 | 0.004 | 2.90E-19 |
| rs12837203 | 23 | 70334233 | G | A | 0.676 | 0.019 | 0.003 | 1.80E-11 |
| rs7884765 | 23 | 1.1E+08 | T | C | 0.393 | 0.024 | 0.003 | 2.20E-17 |
| rs67596711 | 23 | 1.53E+08 | T | G | 0.501 | 0.039 | 0.003 | 2.00E-43 |

**Table S3** – Genome-wide significant single nucleotide polymorphisms (SNP) used as instrumental variants for sex hormone binding globulin in females..

| **SNP** | **Variant chromosome** | **Variant position** | **Effect allele** | **Non-effect allele** | **Effect allele frequency** | **Beta** | **Standard error** | **P-value** |
| --- | --- | --- | --- | --- | --- | --- | --- | --- |
| rs75077113 | 1 | 11214582 | C | A | 0.277 | 0.012 | 0.001 | 2.90E-16 |
| rs198358 | 1 | 11904076 | C | T | 0.248 | 0.008 | 0.001 | 9.00E-11 |
| 1:23747996_GA_G | 1 | 23747996 | G | GA | 0.726 | 0.01 | 0.001 | 6.80E-14 |
| rs35568851 | 1 | 25766501 | CT | C | 0.554 | 0.008 | 0.001 | 1.70E-10 |
| rs114165349 | 1 | 27021913 | G | C | 0.976 | 0.074 | 0.004 | 2.80E-82 |
| rs113408476 | 1 | 35868433 | A | ACTTTTTC | 0.909 | 0.013 | 0.002 | 2.50E-10 |
| rs201468966 | 1 | 39976059 | C | CA | 0.767 | 0.013 | 0.001 | 1.70E-21 |
| rs150584036 | 1 | 51067405 | TAC | T | 0.151 | 0.013 | 0.002 | 1.10E-12 |
| 1:57170378_AATC_A | 1 | 57170378 | A | AATC | 0.117 | 0.01 | 0.002 | 8.10E-09 |
| rs74090351 | 1 | 61705898 | A | G | 0.069 | 0.019 | 0.002 | 5.90E-18 |
| rs469721 | 1 | 91530001 | C | T | 0.803 | 0.011 | 0.002 | 5.40E-12 |
| 1:93787087_TA_T | 1 | 93787087 | T | TA | 0.165 | 0.01 | 0.002 | 4.40E-10 |
| rs1730862 | 1 | 1.08E+08 | G | A | 0.342 | 0.023 | 0.001 | 1.70E-73 |
| rs140584594 | 1 | 1.1E+08 | A | G | 0.27 | 0.015 | 0.001 | 1.90E-30 |
| rs68002561 | 1 | 1.5E+08 | G | A | 0.092 | 0.012 | 0.002 | 3.30E-10 |
| rs116279971 | 1 | 1.51E+08 | C | T | 0.012 | 0.04 | 0.006 | 5.40E-12 |
| rs267733 | 1 | 1.51E+08 | A | G | 0.839 | 0.014 | 0.002 | 3.70E-21 |
| rs9426829 | 1 | 1.55E+08 | C | T | 0.481 | 0.014 | 0.001 | 1.50E-30 |
| 1:155867257_CAA_C | 1 | 1.56E+08 | C | CAA | 0.663 | 0.011 | 0.001 | 1.50E-18 |
| rs10489206 | 1 | 1.68E+08 | C | T | 0.779 | 0.008 | 0.001 | 3.60E-08 |
| rs2064074 | 1 | 1.71E+08 | A | G | 0.528 | 0.006 | 0.001 | 3.60E-08 |
| rs12138803 | 1 | 1.72E+08 | C | T | 0.731 | 0.009 | 0.001 | 9.80E-10 |
| rs34331968 | 1 | 1.97E+08 | T | C | 0.534 | 0.011 | 0.001 | 7.80E-20 |
| rs17583875 | 1 | 1.98E+08 | A | G | 0.021 | 0.029 | 0.004 | 1.70E-12 |
| rs13303359 | 1 | 2.04E+08 | C | A | 0.528 | 0.008 | 0.001 | 5.90E-12 |
| rs202021413 | 1 | 2.05E+08 | TA | T | 0.517 | 0.01 | 0.001 | 5.10E-15 |
| rs1418652 | 1 | 2.06E+08 | C | T | 0.386 | 0.007 | 0.001 | 3.30E-08 |
| rs148911629 | 1 | 2.14E+08 | G | C | 0.991 | 0.037 | 0.006 | 9.50E-09 |
| rs1223796 | 1 | 2.14E+08 | G | C | 0.164 | 0.014 | 0.002 | 8.40E-19 |
| rs3001032 | 1 | 2.2E+08 | C | T | 0.32 | 0.015 | 0.001 | 3.60E-29 |
| rs61830291 | 1 | 2.21E+08 | C | A | 0.097 | 0.012 | 0.002 | 1.70E-08 |
| 1:221048577_TGA_T | 1 | 2.21E+08 | T | TGA | 0.484 | 0.01 | 0.001 | 1.50E-16 |
| rs1870927 | 1 | 2.26E+08 | A | T | 0.622 | 0.008 | 0.001 | 2.00E-11 |
| rs3768420 | 1 | 2.27E+08 | C | T | 0.786 | 0.009 | 0.002 | 1.10E-09 |
| rs146104952 | 1 | 2.35E+08 | CCAAAAAATA | C | 0.114 | 0.011 | 0.002 | 2.40E-09 |
| rs7567544 | 2 | 20392802 | C | G | 0.455 | 0.008 | 0.001 | 5.60E-10 |
| rs1260326 | 2 | 27730940 | C | T | 0.607 | 0.035 | 0.001 | 1.70E-188 |
| rs6736913 | 2 | 42510018 | A | G | 0.021 | 0.037 | 0.004 | 3.60E-21 |
| rs11690748 | 2 | 48584575 | C | G | 0.623 | 0.008 | 0.001 | 9.70E-11 |
| rs921153 | 2 | 61563408 | A | G | 0.159 | 0.009 | 0.002 | 2.80E-10 |
| rs6546096 | 2 | 64906295 | A | G | 0.263 | 0.025 | 0.001 | 3.50E-76 |
| rs12624244 | 2 | 70417138 | A | G | 0.936 | 0.022 | 0.003 | 9.50E-20 |
| rs13394092 | 2 | 85815954 | C | T | 0.17 | 0.008 | 0.002 | 1.40E-08 |
| rs72836346 | 2 | 1.12E+08 | C | G | 0.079 | 0.015 | 0.002 | 6.10E-11 |
| rs12613243 | 2 | 1.12E+08 | T | C | 0.937 | 0.019 | 0.003 | 7.60E-16 |
| rs13018007 | 2 | 1.15E+08 | G | A | 0.082 | 0.013 | 0.002 | 1.20E-08 |
| rs11688682 | 2 | 1.21E+08 | C | G | 0.27 | 0.01 | 0.001 | 1.30E-12 |
| rs1128249 | 2 | 1.66E+08 | T | G | 0.392 | 0.022 | 0.001 | 1.30E-73 |
| rs111270317 | 2 | 1.72E+08 | CGTGT | C | 0.34 | 0.007 | 0.001 | 3.50E-08 |
| rs2364717 | 2 | 1.78E+08 | T | C | 0.538 | 0.008 | 0.001 | 2.30E-12 |
| 2:191559843_GT_G | 2 | 1.92E+08 | GT | G | 0.772 | 0.013 | 0.001 | 4.20E-17 |
| 2:208469213_ATCTT_A | 2 | 2.08E+08 | ATCTT | A | 0.185 | 0.01 | 0.002 | 4.10E-11 |
| rs1047891 | 2 | 2.12E+08 | A | C | 0.315 | 0.018 | 0.001 | 1.50E-44 |
| rs10189479 | 2 | 2.19E+08 | A | C | 0.434 | 0.009 | 0.001 | 4.80E-14 |
| rs78058190 | 2 | 2.2E+08 | G | A | 0.949 | 0.026 | 0.003 | 7.10E-18 |
| rs2176040 | 2 | 2.27E+08 | A | G | 0.354 | 0.015 | 0.001 | 2.70E-36 |
| rs2924808 | 2 | 2.34E+08 | C | G | 0.345 | 0.007 | 0.001 | 2.00E-08 |
| rs62186584 | 2 | 2.42E+08 | C | T | 0.744 | 0.008 | 0.001 | 5.90E-09 |
| rs1801282 | 3 | 12393125 | G | C | 0.12 | 0.024 | 0.002 | 8.90E-43 |
| rs6792725 | 3 | 24520283 | G | A | 0.692 | 0.018 | 0.001 | 4.50E-43 |
| rs784504 | 3 | 39195260 | C | G | 0.812 | 0.01 | 0.002 | 1.70E-12 |
| rs10461018 | 3 | 46995242 | T | C | 0.42 | 0.011 | 0.001 | 2.50E-19 |
| rs549664712 | 3 | 49749648 | C | CA | 0.671 | 0.011 | 0.001 | 2.70E-15 |
| rs6772177 | 3 | 52497778 | C | T | 0.828 | 0.011 | 0.002 | 1.60E-12 |
| rs11130982 | 3 | 64728312 | T | G | 0.293 | 0.009 | 0.001 | 3.80E-11 |
| rs10511002 | 3 | 70631018 | A | C | 0.29 | 0.008 | 0.001 | 2.50E-09 |
| rs4530527 | 3 | 86800085 | C | A | 0.361 | 0.008 | 0.001 | 1.80E-09 |
| rs17202341 | 3 | 1.05E+08 | G | A | 0.348 | 0.007 | 0.001 | 1.30E-09 |
| rs201874554 | 3 | 1.15E+08 | A | ACC | 0.942 | 0.016 | 0.003 | 9.10E-09 |
| 3:122361173_TTTTTC_T | 3 | 1.22E+08 | TTTTTC | T | 0.757 | 0.009 | 0.001 | 9.10E-10 |
| rs11720108 | 3 | 1.23E+08 | T | C | 0.249 | 0.008 | 0.001 | 5.40E-11 |
| rs6803518 | 3 | 1.3E+08 | T | C | 0.754 | 0.007 | 0.001 | 4.60E-08 |
| rs687339 | 3 | 1.36E+08 | C | T | 0.227 | 0.031 | 0.001 | 5.30E-104 |
| rs9872754 | 3 | 1.38E+08 | C | T | 0.84 | 0.01 | 0.002 | 9.00E-11 |
| rs9834503 | 3 | 1.5E+08 | A | C | 0.54 | 0.008 | 0.001 | 1.90E-09 |
| rs62271373 | 3 | 1.5E+08 | T | A | 0.94 | 0.026 | 0.003 | 2.60E-25 |
| rs1126161 | 3 | 1.72E+08 | G | A | 0.673 | 0.008 | 0.001 | 5.60E-09 |
| rs79287178 | 3 | 1.72E+08 | G | A | 0.969 | 0.032 | 0.004 | 4.00E-19 |
| rs59837038 | 3 | 1.72E+08 | T | C | 0.269 | 0.009 | 0.001 | 1.50E-12 |
| rs3749228 | 3 | 1.84E+08 | C | G | 0.062 | 0.016 | 0.003 | 3.20E-10 |
| rs57158761 | 3 | 1.85E+08 | A | G | 0.564 | 0.01 | 0.001 | 9.30E-18 |
| rs34311866 | 4 | 951947 | T | C | 0.824 | 0.011 | 0.002 | 6.10E-14 |
| rs13108218 | 4 | 3443931 | A | G | 0.383 | 0.024 | 0.001 | 1.40E-83 |
| rs4450871 | 4 | 4990298 | G | A | 0.442 | 0.009 | 0.001 | 2.10E-14 |
| rs925098 | 4 | 17919811 | G | A | 0.265 | 0.009 | 0.001 | 2.80E-09 |
| rs2970871 | 4 | 23890582 | T | C | 0.441 | 0.007 | 0.001 | 3.20E-08 |
| rs6531735 | 4 | 39686332 | G | A | 0.492 | 0.006 | 0.001 | 1.30E-08 |
| rs62303689 | 4 | 56295873 | C | A | 0.875 | 0.012 | 0.002 | 3.20E-10 |
| rs7696472 | 4 | 69538180 | A | G | 0.475 | 0.007 | 0.001 | 4.50E-08 |
| rs28636815 | 4 | 77197397 | G | A | 0.377 | 0.012 | 0.001 | 1.50E-21 |
| rs13150068 | 4 | 88203828 | A | G | 0.564 | 0.017 | 0.001 | 6.20E-47 |
| rs34154818 | 4 | 89726823 | A | AT | 0.551 | 0.011 | 0.001 | 9.50E-18 |
| rs6831257 | 4 | 1E+08 | G | A | 0.34 | 0.008 | 0.001 | 3.30E-11 |
| 4:103877471_TA_T | 4 | 1.04E+08 | T | TA | 0.486 | 0.008 | 0.001 | 4.50E-10 |
| rs114816312 | 4 | 1.11E+08 | T | C | 0.008 | 0.083 | 0.007 | 1.10E-34 |
| rs138204164 | 4 | 1.2E+08 | C | G | 0.874 | 0.012 | 0.002 | 1.20E-10 |
| rs1433210 | 4 | 1.25E+08 | C | A | 0.245 | 0.01 | 0.001 | 2.90E-12 |
| rs3733321 | 4 | 1.29E+08 | T | G | 0.646 | 0.007 | 0.001 | 2.30E-08 |
| rs28925904 | 4 | 1.44E+08 | C | T | 0.975 | 0.023 | 0.004 | 4.90E-09 |
| rs10857228 | 4 | 1.49E+08 | T | C | 0.257 | 0.01 | 0.001 | 2.60E-13 |
| rs41280463 | 4 | 1.54E+08 | G | A | 0.833 | 0.01 | 0.002 | 1.30E-10 |
| rs28712547 | 4 | 1.58E+08 | G | A | 0.321 | 0.01 | 0.001 | 2.90E-14 |
| rs78890745 | 4 | 1.6E+08 | A | G | 0.109 | 0.02 | 0.002 | 2.40E-25 |
| rs11738093 | 5 | 53301425 | A | G | 0.748 | 0.014 | 0.001 | 6.00E-24 |
| rs40270 | 5 | 55804552 | A | C | 0.227 | 0.018 | 0.001 | 8.70E-35 |
| rs10939934 | 5 | 61531501 | A | G | 0.422 | 0.008 | 0.001 | 1.90E-10 |
| rs4976033 | 5 | 67714246 | A | G | 0.599 | 0.011 | 0.001 | 3.30E-17 |
| rs34651 | 5 | 72144005 | T | C | 0.918 | 0.011 | 0.002 | 3.00E-08 |
| rs35475471 | 5 | 90158645 | CAT | C | 0.325 | 0.008 | 0.001 | 1.20E-10 |
| rs6860245 | 5 | 1.27E+08 | C | G | 0.247 | 0.011 | 0.001 | 9.60E-14 |
| rs115209326 | 5 | 1.31E+08 | T | C | 0.003 | 0.06 | 0.011 | 3.60E-09 |
| rs2057655 | 5 | 1.32E+08 | A | G | 0.187 | 0.011 | 0.002 | 2.40E-11 |
| rs1650527 | 5 | 1.58E+08 | C | T | 0.768 | 0.014 | 0.001 | 4.10E-22 |
| rs6879874 | 5 | 1.77E+08 | T | A | 0.724 | 0.008 | 0.001 | 1.80E-09 |
| rs72648854 | 5 | 1.8E+08 | C | T | 0.051 | 0.019 | 0.003 | 2.90E-09 |
| rs9379084 | 6 | 7231843 | G | A | 0.884 | 0.013 | 0.002 | 1.50E-10 |
| rs2299055 | 6 | 15398331 | A | G | 0.117 | 0.011 | 0.002 | 2.20E-08 |
| rs9366291 | 6 | 19381870 | C | G | 0.577 | 0.006 | 0.001 | 4.20E-08 |
| rs34499031 | 6 | 20676414 | T | TAA | 0.736 | 0.011 | 0.001 | 1.10E-14 |
| rs1634791 | 6 | 31276777 | A | G | 0.456 | 0.012 | 0.001 | 8.10E-23 |
| rs9461793 | 6 | 32636518 | A | C | 0.722 | 0.011 | 0.001 | 2.00E-16 |
| rs28360642 | 6 | 41667506 | A | C | 0.839 | 0.02 | 0.002 | 5.80E-36 |
| rs11967262 | 6 | 43760327 | C | G | 0.512 | 0.011 | 0.001 | 1.30E-20 |
| rs13200245 | 6 | 95949746 | A | G | 0.841 | 0.009 | 0.002 | 2.20E-08 |
| rs150115323 | 6 | 1.18E+08 | G | C | 0.372 | 0.006 | 0.001 | 4.10E-09 |
| rs58321169 | 6 | 1.27E+08 | C | T | 0.732 | 0.01 | 0.001 | 5.60E-14 |
| 6:130386212_GGAGA_G | 6 | 1.3E+08 | GGAGA | G | 0.326 | 0.011 | 0.001 | 3.00E-17 |
| rs199607859 | 6 | 1.4E+08 | T | G | 0.594 | 0.01 | 0.001 | 3.70E-17 |
| rs1738386 | 6 | 1.52E+08 | C | T | 0.38 | 0.008 | 0.001 | 1.90E-11 |
| rs555754 | 6 | 1.61E+08 | A | G | 0.468 | 0.018 | 0.001 | 1.70E-59 |
| rs4709746 | 6 | 1.64E+08 | T | C | 0.134 | 0.012 | 0.002 | 4.50E-11 |
| rs73670309 | 7 | 1065947 | C | A | 0.894 | 0.012 | 0.002 | 1.10E-10 |
| rs2246223 | 7 | 6701189 | T | C | 0.555 | 0.008 | 0.001 | 3.60E-10 |
| rs10486782 | 7 | 15884421 | A | G | 0.761 | 0.008 | 0.001 | 2.50E-08 |
| rs2723555 | 7 | 17863633 | T | A | 0.488 | 0.006 | 0.001 | 3.50E-08 |
| rs28459049 | 7 | 21567331 | C | T | 0.787 | 0.009 | 0.002 | 1.30E-10 |
| rs4563785 | 7 | 26349213 | G | T | 0.913 | 0.014 | 0.002 | 6.60E-12 |
| rs10622246 | 7 | 28187111 | ATTTT | A | 0.5 | 0.012 | 0.001 | 8.00E-25 |
| rs13237750 | 7 | 46456878 | C | T | 0.952 | 0.016 | 0.003 | 1.60E-09 |
| rs17492269 | 7 | 70047405 | G | A | 0.824 | 0.01 | 0.002 | 2.80E-09 |
| rs799157 | 7 | 73020301 | T | C | 0.043 | 0.017 | 0.003 | 9.00E-09 |
| rs848476 | 7 | 77541673 | G | A | 0.292 | 0.01 | 0.001 | 1.10E-16 |
| rs1229498 | 7 | 81568750 | T | G | 0.275 | 0.011 | 0.001 | 2.40E-16 |
| rs768159759 | 7 | 86880738 | A | AG | 0.075 | 0.014 | 0.002 | 1.50E-09 |
| rs764029425 | 7 | 97984321 | TG | T | 0.816 | 0.038 | 0.002 | 4.50E-135 |
| rs10238028 | 7 | 99208899 | G | A | 0.067 | 0.014 | 0.002 | 6.50E-09 |
| rs6706 | 7 | 1E+08 | T | C | 0.184 | 0.017 | 0.002 | 2.50E-29 |
| rs781996653 | 7 | 1.02E+08 | TTATTTTATTG | T | 0.64 | 0.008 | 0.001 | 1.50E-08 |
| rs11556924 | 7 | 1.3E+08 | T | C | 0.389 | 0.011 | 0.001 | 3.20E-18 |
| 7:130438531_CTTTTTT_C | 7 | 1.3E+08 | C | CTTTTTT | 0.515 | 0.012 | 0.001 | 3.20E-22 |
| rs71531849 | 7 | 1.35E+08 | CT | C | 0.356 | 0.008 | 0.001 | 1.30E-09 |
| rs114949263 | 7 | 1.5E+08 | C | T | 0.111 | 0.014 | 0.002 | 2.70E-14 |
| rs9987289 | 8 | 9183358 | G | A | 0.908 | 0.022 | 0.002 | 1.40E-26 |
| rs62486442 | 8 | 12623463 | G | A | 0.665 | 0.008 | 0.001 | 2.60E-09 |
| rs9644032 | 8 | 23414822 | T | G | 0.368 | 0.008 | 0.001 | 1.20E-11 |
| rs10095380 | 8 | 36853217 | G | C | 0.834 | 0.012 | 0.002 | 7.50E-13 |
| rs12543287 | 8 | 42334511 | C | G | 0.372 | 0.011 | 0.001 | 1.80E-18 |
| 8:59415339_TTG_T | 8 | 59415339 | T | TTG | 0.642 | 0.009 | 0.001 | 4.20E-13 |
| rs555234816 | 8 | 72397810 | T | TA | 0.923 | 0.018 | 0.002 | 1.10E-15 |
| rs76767219 | 8 | 81426196 | A | C | 0.034 | 0.048 | 0.003 | 2.40E-50 |
| rs62515079 | 8 | 81442754 | G | A | 0.02 | 0.032 | 0.005 | 9.20E-15 |
| 8:81457499_CA_C | 8 | 81457499 | C | CA | 0.204 | 0.025 | 0.002 | 6.20E-69 |
| rs10095930 | 8 | 1.17E+08 | C | T | 0.417 | 0.01 | 0.001 | 1.10E-15 |
| rs11774700 | 8 | 1.18E+08 | C | T | 0.309 | 0.008 | 0.001 | 1.50E-09 |
| rs2980858 | 8 | 1.27E+08 | C | T | 0.696 | 0.01 | 0.001 | 4.70E-14 |
| rs4871015 | 8 | 1.28E+08 | A | G | 0.581 | 0.008 | 0.001 | 5.10E-10 |
| rs11780978 | 8 | 1.45E+08 | A | G | 0.403 | 0.009 | 0.001 | 2.80E-13 |
| rs10108150 | 8 | 1.46E+08 | A | G | 0.47 | 0.009 | 0.001 | 1.90E-15 |
| rs868655 | 9 | 1037118 | C | A | 0.692 | 0.009 | 0.001 | 8.60E-12 |
| rs568656 | 9 | 4133874 | C | A | 0.351 | 0.012 | 0.001 | 4.40E-18 |
| rs1330307 | 9 | 4305064 | A | C | 0.515 | 0.008 | 0.001 | 1.10E-12 |
| rs10815276 | 9 | 5737692 | G | A | 0.457 | 0.008 | 0.001 | 1.70E-10 |
| rs820504 | 9 | 6668278 | G | A | 0.864 | 0.014 | 0.002 | 2.20E-16 |
| rs10961205 | 9 | 13722479 | A | G | 0.583 | 0.007 | 0.001 | 4.60E-08 |
| rs696825 | 9 | 86583076 | T | C | 0.252 | 0.024 | 0.001 | 4.50E-63 |
| rs117522510 | 9 | 88886513 | A | G | 0.006 | 0.041 | 0.008 | 3.30E-08 |
| rs4876993 | 9 | 92281403 | T | C | 0.488 | 0.007 | 0.001 | 2.50E-09 |
| rs1962883 | 9 | 1.08E+08 | C | T | 0.53 | 0.008 | 0.001 | 6.70E-10 |
| rs62580766 | 9 | 1.13E+08 | T | C | 0.181 | 0.011 | 0.002 | 7.50E-16 |
| rs2986669 | 9 | 1.13E+08 | A | G | 0.681 | 0.009 | 0.001 | 4.90E-11 |
| rs112332688 | 9 | 1.19E+08 | A | G | 0.768 | 0.008 | 0.001 | 1.30E-08 |
| rs4837794 | 9 | 1.24E+08 | T | C | 0.332 | 0.01 | 0.001 | 1.40E-16 |
| 9:127474886_CA_C | 9 | 1.27E+08 | CA | C | 0.5 | 0.007 | 0.001 | 7.20E-09 |
| rs9697210 | 9 | 1.31E+08 | G | A | 0.854 | 0.015 | 0.002 | 9.00E-20 |
| rs143378550 | 9 | 1.33E+08 | A | C | 0.025 | 0.019 | 0.004 | 3.70E-08 |
| rs8176741 | 9 | 1.36E+08 | G | A | 0.938 | 0.016 | 0.003 | 1.70E-10 |
| rs72766607 | 9 | 1.37E+08 | T | G | 0.98 | 0.032 | 0.004 | 3.10E-15 |
| rs11791747 | 9 | 1.37E+08 | G | A | 0.307 | 0.01 | 0.001 | 2.20E-13 |
| rs80126506 | 9 | 1.37E+08 | A | G | 0.592 | 0.007 | 0.001 | 2.10E-10 |
| rs35233014 | 9 | 1.37E+08 | C | A | 0.255 | 0.015 | 0.001 | 3.80E-27 |
| rs11103377 | 9 | 1.39E+08 | G | A | 0.538 | 0.007 | 0.001 | 6.00E-11 |
| rs7475279 | 10 | 5252866 | A | C | 0.846 | 0.018 | 0.002 | 1.30E-30 |
| rs899865 | 10 | 36473044 | T | C | 0.6 | 0.007 | 0.001 | 1.00E-08 |
| rs1530439 | 10 | 63645959 | T | G | 0.309 | 0.011 | 0.001 | 9.70E-19 |
| 10:65158772_AAAG_A | 10 | 65158772 | A | AAAG | 0.472 | 0.044 | 0.001 | 5.7E-310 |
| rs35696875 | 10 | 70982136 | TCA | T | 0.694 | 0.011 | 0.001 | 8.50E-22 |
| rs2915023 | 10 | 77282899 | G | A | 0.903 | 0.011 | 0.002 | 3.90E-08 |
| rs1782652 | 10 | 81074125 | T | A | 0.619 | 0.014 | 0.001 | 1.20E-26 |
| rs11186719 | 10 | 93631956 | A | C | 0.522 | 0.012 | 0.001 | 9.30E-25 |
| rs2068888 | 10 | 94839642 | A | G | 0.451 | 0.013 | 0.001 | 2.00E-26 |
| rs11188601 | 10 | 97856899 | C | T | 0.364 | 0.01 | 0.001 | 6.10E-17 |
| rs10883451 | 10 | 1.02E+08 | C | T | 0.499 | 0.009 | 0.001 | 8.20E-11 |
| rs140312320 | 10 | 1.04E+08 | G | A | 0.933 | 0.017 | 0.002 | 1.10E-11 |
| rs548235873 | 10 | 1.05E+08 | ATTT | A | 0.222 | 0.01 | 0.001 | 6.60E-11 |
| rs35198068 | 10 | 1.15E+08 | T | C | 0.71 | 0.011 | 0.001 | 2.00E-19 |
| rs80235628 | 10 | 1.23E+08 | G | A | 0.95 | 0.025 | 0.003 | 2.00E-17 |
| rs7481219 | 11 | 307808 | A | G | 0.413 | 0.008 | 0.001 | 3.50E-10 |
| rs775181992 | 11 | 2179864 | A | AGCCCT | 0.24 | 0.012 | 0.001 | 1.30E-16 |
| rs11601507 | 11 | 5701074 | A | C | 0.069 | 0.018 | 0.002 | 3.20E-15 |
| rs1037169 | 11 | 13361005 | T | C | 0.313 | 0.013 | 0.001 | 1.20E-24 |
| rs778571122 | 11 | 16247860 | GTGTTTTTTTTGTTTTTGTTTT | G | 0.613 | 0.011 | 0.001 | 4.70E-22 |
| rs76491020 | 11 | 32673898 | C | G | 0.094 | 0.012 | 0.002 | 1.30E-09 |
| rs5791099 | 11 | 36328427 | TA | T | 0.37 | 0.007 | 0.001 | 1.90E-08 |
| rs185044544 | 11 | 58867435 | T | G | 0.975 | 0.022 | 0.004 | 5.30E-09 |
| rs174537 | 11 | 61552680 | G | T | 0.654 | 0.012 | 0.001 | 5.50E-21 |
| rs71468663 | 11 | 64018104 | A | AC | 0.953 | 0.017 | 0.003 | 2.10E-11 |
| rs12797706 | 11 | 65561369 | A | G | 0.235 | 0.013 | 0.001 | 1.60E-19 |
| rs3018695 | 11 | 68911500 | A | C | 0.493 | 0.009 | 0.001 | 9.30E-14 |
| rs12280075 | 11 | 69170389 | G | T | 0.32 | 0.008 | 0.001 | 3.70E-10 |
| rs12804411 | 11 | 69284200 | T | C | 0.231 | 0.014 | 0.001 | 2.60E-24 |
| rs11021232 | 11 | 95320808 | T | C | 0.82 | 0.013 | 0.002 | 4.00E-17 |
| rs73519353 | 11 | 95380754 | A | T | 0.003 | 0.07 | 0.011 | 2.00E-10 |
| rs12787996 | 11 | 1.02E+08 | C | A | 0.661 | 0.009 | 0.001 | 2.10E-12 |
| rs770971500 | 11 | 1.19E+08 | C | CTTTT | 0.238 | 0.008 | 0.002 | 4.60E-09 |
| rs10893876 | 11 | 1.28E+08 | C | T | 0.766 | 0.008 | 0.001 | 3.50E-08 |
| rs740893 | 12 | 676209 | G | C | 0.214 | 0.009 | 0.002 | 5.00E-11 |
| rs10774095 | 12 | 3125648 | A | G | 0.807 | 0.009 | 0.002 | 7.40E-09 |
| rs76895963 | 12 | 4384844 | G | T | 0.021 | 0.075 | 0.005 | 1.30E-64 |
| rs17887160 | 12 | 6877721 | C | T | 0.72 | 0.009 | 0.001 | 7.40E-12 |
| rs3782735 | 12 | 6885076 | A | G | 0.601 | 0.01 | 0.001 | 5.60E-15 |
| rs11054861 | 12 | 7769947 | A | G | 0.973 | 0.023 | 0.004 | 3.00E-10 |
| 12:12562340_GT_G | 12 | 12562340 | G | GT | 0.145 | 0.009 | 0.002 | 5.00E-09 |
| rs7298820 | 12 | 20581339 | T | A | 0.205 | 0.015 | 0.002 | 1.00E-23 |
| rs4149056 | 12 | 21331549 | T | C | 0.849 | 0.03 | 0.002 | 1.50E-74 |
| rs11047237 | 12 | 24206326 | A | T | 0.965 | 0.028 | 0.003 | 8.20E-17 |
| rs75130744 | 12 | 25410741 | G | C | 0.929 | 0.026 | 0.002 | 3.90E-31 |
| rs1872992 | 12 | 26457190 | A | G | 0.756 | 0.009 | 0.001 | 3.60E-11 |
| 12:26614614_CAT_C | 12 | 26614614 | C | CAT | 0.263 | 0.009 | 0.001 | 1.20E-10 |
| rs10880868 | 12 | 46322449 | C | T | 0.789 | 0.008 | 0.002 | 4.40E-08 |
| rs4307773 | 12 | 51144432 | T | C | 0.419 | 0.014 | 0.001 | 5.90E-31 |
| rs10747689 | 12 | 54425471 | T | C | 0.368 | 0.007 | 0.001 | 4.70E-09 |
| rs7484541 | 12 | 57714803 | T | A | 0.228 | 0.016 | 0.001 | 3.00E-29 |
| rs8756 | 12 | 66359752 | C | A | 0.485 | 0.008 | 0.001 | 1.70E-13 |
| rs2438109 | 12 | 67653617 | C | T | 0.657 | 0.007 | 0.001 | 1.60E-08 |
| rs147153202 | 12 | 93631004 | G | GA | 0.865 | 0.013 | 0.002 | 4.90E-14 |
| rs528806375 | 12 | 95881367 | A | AT | 0.564 | 0.007 | 0.001 | 2.10E-09 |
| rs191591035 | 12 | 99868285 | G | C | 0.997 | 0.099 | 0.014 | 6.90E-14 |
| rs61755050 | 12 | 1.01E+08 | T | C | 0.994 | 0.131 | 0.008 | 1.10E-72 |
| rs3751129 | 12 | 1.02E+08 | A | G | 0.216 | 0.011 | 0.001 | 1.70E-13 |
| 12:103483327_AT_A | 12 | 1.03E+08 | AT | A | 0.521 | 0.024 | 0.001 | 2.40E-91 |
| rs11830764 | 12 | 1.12E+08 | C | G | 0.068 | 0.042 | 0.002 | 9.00E-68 |
| rs183015141 | 12 | 1.12E+08 | G | A | 0.973 | 0.024 | 0.004 | 9.60E-11 |
| rs7139079 | 12 | 1.21E+08 | A | G | 0.593 | 0.013 | 0.001 | 5.40E-28 |
| rs12311848 | 12 | 1.24E+08 | G | A | 0.333 | 0.014 | 0.001 | 1.60E-29 |
| 13:50565104_ACT_A | 13 | 50565104 | A | ACT | 0.02 | 0.037 | 0.004 | 4.00E-19 |
| rs12864658 | 13 | 50666074 | T | C | 0.051 | 0.019 | 0.003 | 1.90E-12 |
| rs9556403 | 13 | 95236825 | G | A | 0.35 | 0.007 | 0.001 | 6.80E-10 |
| rs7321688 | 13 | 1.15E+08 | C | A | 0.767 | 0.009 | 0.001 | 1.70E-09 |
| rs17128091 | 14 | 23714682 | C | G | 0.256 | 0.013 | 0.001 | 3.20E-20 |
| rs11621792 | 14 | 24871926 | C | T | 0.548 | 0.026 | 0.001 | 4.00E-102 |
| rs72681869 | 14 | 50655357 | C | G | 0.011 | 0.047 | 0.006 | 3.90E-16 |
| rs11376788 | 14 | 69314054 | AC | A | 0.243 | 0.008 | 0.001 | 1.40E-08 |
| rs2239222 | 14 | 73011885 | G | A | 0.349 | 0.01 | 0.001 | 2.10E-16 |
| rs13379043 | 14 | 74250126 | C | T | 0.278 | 0.011 | 0.001 | 2.20E-16 |
| rs1005421 | 14 | 89886940 | C | T | 0.583 | 0.008 | 0.001 | 1.60E-12 |
| rs28929474 | 14 | 94844947 | T | C | 0.02 | 0.061 | 0.004 | 3.10E-43 |
| rs17580 | 14 | 94847262 | A | T | 0.048 | 0.024 | 0.003 | 8.90E-18 |
| rs28929470 | 14 | 94847386 | A | G | 0.004 | 0.052 | 0.01 | 4.40E-09 |
| rs34184867 | 14 | 1.01E+08 | C | G | 0.505 | 0.01 | 0.001 | 4.60E-16 |
| rs2498786 | 14 | 1.05E+08 | C | G | 0.385 | 0.011 | 0.001 | 5.50E-19 |
| rs12593818 | 15 | 35153930 | T | C | 0.263 | 0.009 | 0.001 | 2.40E-11 |
| rs275177 | 15 | 39449003 | C | T | 0.15 | 0.01 | 0.002 | 5.50E-09 |
| rs11637595 | 15 | 40387728 | C | T | 0.724 | 0.012 | 0.001 | 2.40E-18 |
| rs2009310 | 15 | 41965591 | T | G | 0.496 | 0.008 | 0.001 | 1.10E-12 |
| 15:43025006_CT_C | 15 | 43025006 | CT | C | 0.997 | 0.125 | 0.016 | 4.90E-17 |
| rs139974673 | 15 | 44027885 | T | C | 0.974 | 0.054 | 0.004 | 1.40E-49 |
| 15:51052455_CT_C | 15 | 51052455 | C | CT | 0.348 | 0.007 | 0.001 | 3.40E-09 |
| 15:53094375_TTTTG_T | 15 | 53094375 | TTTTG | T | 0.886 | 0.027 | 0.002 | 6.30E-47 |
| rs79391862 | 15 | 53739426 | A | C | 0.986 | 0.073 | 0.005 | 1.70E-46 |
| rs79237700 | 15 | 53741612 | T | C | 0.962 | 0.02 | 0.003 | 7.70E-11 |
| rs8027064 | 15 | 53741826 | A | G | 0.035 | 0.027 | 0.003 | 1.50E-15 |
| rs528350911 | 15 | 53747228 | C | G | 0.994 | 0.057 | 0.008 | 4.10E-13 |
| rs12438742 | 15 | 61947280 | G | C | 0.57 | 0.007 | 0.001 | 2.90E-08 |
| rs5813220 | 15 | 63792758 | G | GT | 0.659 | 0.016 | 0.001 | 1.10E-39 |
| rs12906447 | 15 | 96224270 | C | T | 0.552 | 0.009 | 0.001 | 4.30E-14 |
| rs56332871 | 15 | 96714816 | A | C | 0.272 | 0.039 | 0.001 | 9.20E-188 |
| rs1684608 | 16 | 4676852 | C | A | 0.807 | 0.009 | 0.002 | 1.50E-10 |
| rs72782727 | 16 | 11878033 | G | T | 0.754 | 0.009 | 0.001 | 2.30E-11 |
| rs4122352 | 16 | 15174571 | A | G | 0.296 | 0.011 | 0.001 | 1.80E-16 |
| rs144293483 | 16 | 71643415 | AC | A | 0.11 | 0.013 | 0.002 | 5.70E-10 |
| rs2925979 | 16 | 81534790 | C | T | 0.7 | 0.014 | 0.001 | 1.00E-26 |
| rs67890964 | 16 | 83979317 | C | T | 0.374 | 0.011 | 0.001 | 7.00E-19 |
| rs11641834 | 16 | 88070573 | C | T | 0.571 | 0.01 | 0.001 | 3.10E-17 |
| rs67651018 | 16 | 88527222 | A | G | 0.307 | 0.007 | 0.001 | 9.90E-09 |
| rs11078597 | 17 | 1618363 | C | T | 0.186 | 0.016 | 0.002 | 1.30E-25 |
| rs545206972 | 17 | 7491331 | C | T | 0.993 | 0.677 | 0.007 | 8.1E-1919 |
| rs858519 | 17 | 7531965 | C | T | 0.557 | 0.099 | 0.001 | 1.7E-1533 |
| rs560436873 | 17 | 8621357 | A | T | 0.998 | 0.135 | 0.016 | 5.70E-17 |
| rs17669311 | 17 | 13837051 | G | A | 0.61 | 0.009 | 0.001 | 2.30E-14 |
| rs112966033 | 17 | 17724466 | TACACAC | T | 0.606 | 0.016 | 0.001 | 1.00E-37 |
| rs2525570 | 17 | 29681245 | G | A | 0.601 | 0.007 | 0.001 | 1.00E-09 |
| rs17616365 | 17 | 38256401 | G | A | 0.968 | 0.028 | 0.003 | 7.10E-19 |
| rs5820605 | 17 | 44102682 | CT | C | 0.414 | 0.008 | 0.001 | 3.50E-11 |
| rs4264433 | 17 | 45737275 | T | A | 0.514 | 0.02 | 0.001 | 7.30E-62 |
| rs140302625 | 17 | 47379867 | T | G | 0.087 | 0.062 | 0.002 | 4.20E-193 |
| 17:48627860_CCT_C | 17 | 48627860 | C | CCT | 0.246 | 0.009 | 0.001 | 2.00E-11 |
| rs8178824 | 17 | 64224775 | C | T | 0.97 | 0.043 | 0.004 | 9.40E-36 |
| rs11079685 | 17 | 65257494 | A | G | 0.522 | 0.008 | 0.001 | 1.70E-12 |
| rs740516 | 17 | 67082962 | C | G | 0.849 | 0.009 | 0.002 | 2.40E-08 |
| rs72844546 | 17 | 73149850 | C | T | 0.346 | 0.01 | 0.001 | 3.10E-18 |
| rs78057960 | 17 | 73779075 | T | C | 0.294 | 0.011 | 0.001 | 4.00E-18 |
| rs10153315 | 17 | 79481772 | T | C | 0.581 | 0.009 | 0.001 | 7.50E-16 |
| rs11664106 | 18 | 2846812 | T | A | 0.373 | 0.008 | 0.001 | 3.60E-10 |
| rs4092465 | 18 | 55080437 | G | A | 0.649 | 0.012 | 0.001 | 3.60E-20 |
| rs12454712 | 18 | 60845884 | C | T | 0.376 | 0.01 | 0.001 | 3.20E-18 |
| rs34385891 | 18 | 60912627 | AT | A | 0.571 | 0.009 | 0.001 | 4.80E-13 |
| rs4327143 | 18 | 71925113 | A | G | 0.714 | 0.009 | 0.001 | 1.70E-11 |
| rs892225 | 19 | 1152656 | G | A | 0.38 | 0.008 | 0.001 | 2.00E-12 |
| rs550767741 | 19 | 2786763 | ATTTTTTTTTTTTTTTTTTTTTTTTTTTTTTTT | A | 0.295 | 0.021 | 0.001 | 1.10E-60 |
| rs11539938 | 19 | 3062857 | C | T | 0.421 | 0.009 | 0.001 | 7.20E-12 |
| rs60018147 | 19 | 3375572 | G | A | 0.12 | 0.016 | 0.002 | 6.10E-15 |
| rs2288926 | 19 | 4498245 | A | G | 0.311 | 0.007 | 0.001 | 9.00E-09 |
| 19:7223973_TTTG_T | 19 | 7223973 | TTTG | T | 0.579 | 0.019 | 0.001 | 4.40E-54 |
| rs8107967 | 19 | 7972615 | G | A | 0.567 | 0.009 | 0.001 | 5.00E-14 |
| rs4804669 | 19 | 12502457 | A | G | 0.217 | 0.009 | 0.002 | 5.70E-10 |
| rs7252372 | 19 | 14172896 | G | C | 0.557 | 0.007 | 0.001 | 1.70E-09 |
| rs202200760 | 19 | 17346854 | C | G | 0.039 | 0.076 | 0.003 | 5.20E-120 |
| rs111288118 | 19 | 18225002 | C | CTT | 0.755 | 0.011 | 0.001 | 1.40E-14 |
| rs58489806 | 19 | 19456917 | C | T | 0.913 | 0.012 | 0.002 | 4.90E-09 |
| rs7250869 | 19 | 33887405 | C | T | 0.69 | 0.011 | 0.001 | 2.70E-20 |
| rs2018519 | 19 | 35559787 | C | T | 0.181 | 0.02 | 0.002 | 3.40E-40 |
| rs11666245 | 19 | 38229926 | G | A | 0.954 | 0.016 | 0.003 | 1.80E-09 |
| rs5117 | 19 | 45418790 | C | T | 0.235 | 0.011 | 0.001 | 3.20E-15 |
| rs5112 | 19 | 45430280 | G | C | 0.533 | 0.009 | 0.001 | 2.20E-13 |
| rs73036519 | 19 | 45748362 | G | C | 0.7 | 0.009 | 0.001 | 7.30E-10 |
| rs34255979 | 19 | 46384830 | T | C | 0.121 | 0.028 | 0.002 | 7.90E-52 |
| rs62128735 | 19 | 48113604 | A | G | 0.697 | 0.01 | 0.001 | 1.80E-15 |
| rs59774409 | 19 | 50016748 | T | C | 0.082 | 0.018 | 0.002 | 5.20E-16 |
| rs4077285 | 19 | 56599405 | G | C | 0.906 | 0.011 | 0.002 | 1.80E-08 |
| rs11668201 | 19 | 59003632 | T | A | 0.19 | 0.009 | 0.002 | 1.00E-08 |
| rs1741344 | 20 | 4101800 | T | C | 0.634 | 0.007 | 0.001 | 3.40E-09 |
| rs771193934 | 20 | 17844518 | AGGCATGCTGCCAAGAAT | A | 0.343 | 0.01 | 0.001 | 3.80E-14 |
| rs13042148 | 20 | 32298286 | C | T | 0.845 | 0.016 | 0.002 | 1.90E-21 |
| rs6058067 | 20 | 33095095 | G | T | 0.305 | 0.009 | 0.001 | 3.80E-15 |
| rs6088776 | 20 | 33835773 | T | C | 0.861 | 0.01 | 0.002 | 1.70E-10 |
| rs2207132 | 20 | 39142516 | G | A | 0.966 | 0.028 | 0.003 | 9.30E-18 |
| rs6129778 | 20 | 39870578 | A | C | 0.19 | 0.012 | 0.002 | 1.60E-15 |
| rs6073431 | 20 | 43040569 | T | C | 0.532 | 0.017 | 0.001 | 6.20E-43 |
| rs4810580 | 20 | 45594295 | T | G | 0.782 | 0.01 | 0.002 | 6.20E-10 |
| rs16995626 | 20 | 49540925 | C | T | 0.072 | 0.018 | 0.002 | 3.70E-15 |
| rs390408 | 22 | 21912272 | A | G | 0.18 | 0.014 | 0.002 | 2.40E-18 |
| rs1033667 | 22 | 29130300 | T | C | 0.3 | 0.009 | 0.001 | 2.50E-12 |
| rs5753111 | 22 | 30779211 | T | C | 0.291 | 0.013 | 0.001 | 4.40E-23 |
| rs5750131 | 22 | 36007421 | G | A | 0.616 | 0.007 | 0.001 | 4.60E-08 |
| rs3747207 | 22 | 44324855 | A | G | 0.214 | 0.017 | 0.002 | 1.00E-30 |
| rs35143646 | 23 | 2856155 | T | C | 0.658 | 0.009 | 0.001 | 1.10E-12 |
| rs4830411 | 23 | 9425574 | G | A | 0.422 | 0.008 | 0.001 | 9.70E-11 |
| rs3747367 | 23 | 16852327 | A | C | 0.695 | 0.008 | 0.001 | 2.70E-09 |
| rs12012896 | 23 | 1.1E+08 | A | G | 0.393 | 0.027 | 0.001 | 8.80E-115 |

**Table S4** – Genome-wide significant single nucleotide polymorphisms (SNP) used as instrumental variants for bioavailable testosterone in males..

| **SNP** | **Variant chromosome** | **Variant position** | **Effect allele** | **Non-effect allele** | **Effect allele frequency** | **Beta** | **Standard error** | **P-value** |
| --- | --- | --- | --- | --- | --- | --- | --- | --- |
| rs72664935 | 1 | 32274901 | C | T | 0.575 | 0.019 | 0.003 | 1.7E-09 |
| rs12410063 | 1 | 65921318 | C | T | 0.444 | 0.026 | 0.003 | 4.5E-18 |
| rs71519251 | 1 | 163251833 | A | G | 0.167 | 0.024 | 0.004 | 1.5E-09 |
| rs35737316 | 1 | 204161534 | T | C | 0.246 | 0.04 | 0.004 | 4.7E-32 |
| rs13028479 | 2 | 11712075 | G | T | 0.446 | 0.031 | 0.003 | 2.7E-24 |
| rs6729954 | 2 | 18286651 | T | A | 0.435 | 0.019 | 0.003 | 3.3E-10 |
| rs829593 | 2 | 30641234 | G | A | 0.687 | 0.018 | 0.003 | 1.5E-08 |
| rs113017476 | 2 | 31989359 | A | G | 0.039 | 0.182 | 0.008 | 2.9E-122 |
| rs2438086 | 2 | 105871129 | G | A | 0.576 | 0.022 | 0.003 | 6.9E-13 |
| rs2594948 | 2 | 177526156 | T | C | 0.689 | 0.018 | 0.003 | 1.9E-08 |
| rs6718154 | 2 | 180497923 | T | C | 0.275 | 0.032 | 0.003 | 1.2E-22 |
| rs2011425 | 2 | 234627608 | T | G | 0.92 | 0.05 | 0.006 | 6.4E-20 |
| rs1112195 | 3 | 24085166 | G | A | 0.495 | 0.018 | 0.003 | 4.7E-10 |
| rs9824196 | 3 | 28807441 | T | G | 0.721 | 0.026 | 0.003 | 4.1E-16 |
| rs3821866 | 3 | 53805577 | G | C | 0.622 | 0.022 | 0.003 | 4.1E-14 |
| rs66956368 | 3 | 61279726 | T | A | 0.703 | 0.034 | 0.004 | 8.1E-23 |
| rs13065463 | 3 | 61662996 | G | A | 0.869 | 0.032 | 0.005 | 8.8E-13 |
| rs10510939 | 3 | 65507808 | C | T | 0.375 | 0.017 | 0.003 | 1.6E-08 |
| 3:88173922_GT_G | 3 | 88173922 | GT | G | 0.155 | 0.032 | 0.004 | 2.8E-15 |
| rs34040779 | 3 | 107235109 | T | C | 0.924 | 0.035 | 0.006 | 2.2E-09 |
| rs4678408 | 3 | 138053187 | G | A | 0.63 | 0.026 | 0.003 | 7.6E-17 |
| 3:152149372_CTT_C | 3 | 152149372 | C | CTT | 0.101 | 0.038 | 0.005 | 1E-14 |
| rs61762319 | 3 | 154801978 | G | A | 0.029 | 0.05 | 0.009 | 2.7E-08 |
| rs7679843 | 4 | 22028079 | G | C | 0.095 | 0.05 | 0.005 | 3.8E-22 |
| rs4274916 | 4 | 69988378 | C | T | 0.542 | 0.017 | 0.003 | 3.2E-09 |
| rs17254118 | 4 | 103651441 | C | T | 0.981 | 0.109 | 0.011 | 5.1E-24 |
| rs565931739 | 4 | 104590005 | C | CA | 0.836 | 0.047 | 0.004 | 1.1E-29 |
| rs528845403 | 4 | 104780790 | A | AATGTGT | 0.99 | 0.289 | 0.016 | 1.7E-70 |
| rs532320857 | 4 | 106230148 | C | T | 0.996 | 0.171 | 0.029 | 4E-09 |
| rs375286460 | 4 | 169077371 | ATAT | A | 0.562 | 0.019 | 0.003 | 2E-10 |
| rs72774885 | 5 | 95840231 | C | T | 0.166 | 0.027 | 0.004 | 3.5E-10 |
| rs3733897 | 5 | 134223593 | A | G | 0.866 | 0.025 | 0.004 | 3.8E-09 |
| rs950716 | 5 | 135680540 | A | G | 0.863 | 0.034 | 0.004 | 3.1E-14 |
| rs2961853 | 5 | 165932048 | C | T | 0.469 | 0.019 | 0.003 | 3.2E-10 |
| rs34192788 | 6 | 17416258 | T | A | 0.69 | 0.02 | 0.003 | 4.2E-09 |
| rs79310511 | 6 | 27496598 | G | A | 0.048 | 0.043 | 0.007 | 6.5E-10 |
| rs1611581 | 6 | 29850827 | C | T | 0.682 | 0.019 | 0.003 | 4.7E-08 |
| rs539017487 | 6 | 30903445 | GT | G | 0.366 | 0.039 | 0.003 | 4E-26 |
| rs204995 | 6 | 32154285 | A | G | 0.736 | 0.027 | 0.003 | 3.2E-13 |
| rs11751920 | 6 | 34655818 | G | C | 0.978 | 0.061 | 0.01 | 4.6E-09 |
| rs7454964 | 6 | 52728059 | T | C | 0.428 | 0.017 | 0.003 | 1.8E-08 |
| 6:100108564_TG_T | 6 | 100108564 | T | TG | 0.454 | 0.029 | 0.003 | 2.9E-22 |
| rs9322822 | 6 | 105369598 | C | T | 0.679 | 0.049 | 0.003 | 2.1E-52 |
| rs2184968 | 6 | 126760994 | C | T | 0.451 | 0.02 | 0.003 | 9.1E-11 |
| rs71753454 | 6 | 131929309 | A | AC | 0.784 | 0.02 | 0.004 | 3.6E-08 |
| rs190930099 | 6 | 152140576 | G | A | 0.005 | 0.135 | 0.021 | 8.1E-11 |
| rs112714881 | 6 | 152343950 | T | TAC | 0.675 | 0.019 | 0.003 | 6.5E-09 |
| rs9986829 | 7 | 15019259 | A | G | 0.507 | 0.056 | 0.003 | 2.8E-76 |
| rs10279715 | 7 | 40870935 | A | G | 0.538 | 0.022 | 0.003 | 3.1E-13 |
| rs1178947 | 7 | 72850178 | T | C | 0.796 | 0.024 | 0.004 | 5.6E-10 |
| 7:78482336_AG_A | 7 | 78482336 | AG | A | 0.964 | 0.075 | 0.009 | 3.7E-19 |
| rs55795858 | 7 | 146123500 | C | T | 0.329 | 0.018 | 0.003 | 1.5E-08 |
| rs2631864 | 8 | 21112084 | G | A | 0.104 | 0.033 | 0.005 | 8.3E-12 |
| rs4872310 | 8 | 25247181 | G | A | 0.753 | 0.023 | 0.004 | 3.7E-12 |
| rs4503095 | 8 | 34837357 | G | A | 0.91 | 0.03 | 0.005 | 1.6E-08 |
| rs4562360 | 8 | 61704817 | G | A | 0.757 | 0.032 | 0.004 | 3.6E-20 |
| rs71529289 | 8 | 77879487 | C | T | 0.748 | 0.036 | 0.003 | 2E-25 |
| rs4483209 | 9 | 1960629 | T | G | 0.472 | 0.017 | 0.003 | 4.3E-09 |
| rs745486 | 9 | 11242155 | C | T | 0.719 | 0.021 | 0.003 | 1.2E-10 |
| rs10738700 | 9 | 24973797 | A | G | 0.569 | 0.02 | 0.003 | 5.6E-11 |
| rs912202 | 9 | 77225603 | C | G | 0.343 | 0.04 | 0.003 | 2.5E-38 |
| rs2090409 | 9 | 108967088 | C | A | 0.684 | 0.031 | 0.003 | 1.7E-21 |
| rs10982156 | 9 | 117088064 | A | T | 0.07 | 0.048 | 0.006 | 4.5E-14 |
| rs7872329 | 9 | 131956152 | A | T | 0.689 | 0.017 | 0.003 | 3.8E-08 |
| 9:140774721_T_C | 9 | 140774721 | C | T | 0.489 | 0.023 | 0.003 | 6.8E-14 |
| rs7912521 | 10 | 67262089 | C | T | 0.416 | 0.061 | 0.003 | 6.2E-94 |
| rs796177770 | 10 | 101746911 | C | CA | 0.604 | 0.019 | 0.003 | 2E-09 |
| rs4919686 | 10 | 104592249 | A | C | 0.71 | 0.023 | 0.003 | 3.9E-13 |
| rs7915430 | 10 | 121660465 | T | G | 0.798 | 0.021 | 0.004 | 9.7E-09 |
| rs11389722 | 11 | 28077708 | G | GT | 0.512 | 0.02 | 0.003 | 1.2E-10 |
| rs116923389 | 11 | 29184521 | T | C | 0.981 | 0.098 | 0.011 | 5.5E-19 |
| rs2035837 | 11 | 29200527 | T | C | 0.852 | 0.073 | 0.004 | 6.7E-67 |
| rs55765314 | 11 | 72360935 | C | A | 0.838 | 0.025 | 0.004 | 4.6E-10 |
| rs12796488 | 11 | 94131557 | C | A | 0.823 | 0.056 | 0.004 | 6.9E-46 |
| rs503542 | 11 | 118590743 | G | A | 0.45 | 0.018 | 0.003 | 5.8E-10 |
| rs10892924 | 11 | 122773715 | T | A | 0.568 | 0.039 | 0.003 | 2.8E-37 |
| rs618888 | 11 | 125081521 | T | G | 0.283 | 0.028 | 0.003 | 1.4E-18 |
| rs56196860 | 12 | 2908330 | A | C | 0.032 | 0.321 | 0.009 | 5.4E-310 |
| rs34138930 | 12 | 57359261 | C | CA | 0.886 | 0.038 | 0.005 | 3.6E-15 |
| rs61932784 | 12 | 114132310 | C | A | 0.783 | 0.022 | 0.004 | 4E-09 |
| rs12810788 | 12 | 116196322 | G | A | 0.201 | 0.027 | 0.004 | 1.1E-11 |
| rs6486542 | 12 | 130952209 | C | T | 0.571 | 0.026 | 0.003 | 2.9E-18 |
| rs2038695 | 13 | 100559123 | C | A | 0.45 | 0.023 | 0.003 | 2.6E-15 |
| rs137969745 | 13 | 112723111 | C | CGGGCGT | 0.911 | 0.045 | 0.005 | 7.4E-17 |
| rs10137488 | 14 | 35797122 | C | T | 0.027 | 0.053 | 0.01 | 2.1E-08 |
| rs1272131 | 14 | 60886150 | C | T | 0.387 | 0.027 | 0.003 | 1.4E-17 |
| rs1812755 | 14 | 90007637 | T | C | 0.8 | 0.034 | 0.004 | 5.1E-19 |
| rs1454836 | 15 | 47551054 | T | A | 0.601 | 0.017 | 0.003 | 3.4E-08 |
| rs17703883 | 15 | 51530097 | C | T | 0.255 | 0.044 | 0.004 | 7.5E-40 |
| rs7496293 | 15 | 57300952 | T | C | 0.563 | 0.023 | 0.003 | 4.3E-14 |
| rs12907068 | 15 | 60759011 | G | A | 0.137 | 0.023 | 0.004 | 1.8E-08 |
| rs13835 | 15 | 89056040 | A | C | 0.424 | 0.019 | 0.003 | 4.9E-10 |
| rs2764772 | 16 | 20060653 | A | T | 0.334 | 0.037 | 0.003 | 5.4E-33 |
| rs8061590 | 16 | 28895130 | A | G | 0.597 | 0.029 | 0.003 | 3.4E-21 |
| 16:53822169_AT_A | 16 | 53822169 | AT | A | 0.653 | 0.018 | 0.003 | 1.1E-08 |
| rs62041532 | 16 | 73922719 | G | T | 0.213 | 0.023 | 0.004 | 1.6E-10 |
| rs545206972 | 17 | 7491331 | T | C | 0.007 | 0.252 | 0.019 | 4E-43 |
| rs1799941 | 17 | 7533423 | G | A | 0.739 | 0.034 | 0.003 | 7.7E-23 |
| rs58879558 | 17 | 44095467 | C | T | 0.23 | 0.023 | 0.004 | 2.4E-09 |
| rs8076703 | 17 | 75612643 | C | T | 0.296 | 0.024 | 0.003 | 1E-12 |
| rs2668776 | 18 | 44750365 | C | T | 0.469 | 0.029 | 0.003 | 1.7E-22 |
| 19:48369728_ATTT_A | 19 | 48369728 | A | ATTT | 0.853 | 0.028 | 0.004 | 1.9E-11 |
| rs6521 | 19 | 49519873 | C | G | 0.567 | 0.019 | 0.003 | 1.3E-10 |
| rs2327121 | 20 | 8878250 | C | G | 0.657 | 0.018 | 0.003 | 1.3E-08 |
| rs7265992 | 20 | 33525407 | G | A | 0.821 | 0.032 | 0.004 | 4E-16 |
| 22:31358580_CA_C | 22 | 31358580 | C | CA | 0.346 | 0.018 | 0.003 | 4.9E-08 |
| rs11703376 | 22 | 49678713 | T | C | 0.27 | 0.04 | 0.003 | 5.9E-32 |
| rs111386834 | 23 | 8906695 | C | T | 0.268 | 0.132 | 0.002 | 3.0E-670 |
| rs138271349 | 23 | 11209184 | T | C | 0.05 | 0.028 | 0.005 | 6.2E-09 |
| rs1034948 | 23 | 30331349 | G | A | 0.867 | 0.018 | 0.003 | 3.7E-09 |
| rs5915287 | 23 | 50372904 | G | T | 0.391 | 0.012 | 0.002 | 1.2E-08 |
| rs140498714 | 23 | 53317280 | C | T | 0.983 | 0.054 | 0.01 | 2.3E-08 |
| X:54424009_CT_C | 23 | 54424009 | CT | C | 0.912 | 0.027 | 0.005 | 4.8E-10 |
| rs5913997 | 23 | 56815741 | G | C | 0.196 | 0.053 | 0.003 | 8.7E-90 |
| rs146225865 | 23 | 63265012 | G | A | 0.987 | 0.094 | 0.01 | 3E-25 |
| rs73221538 | 23 | 65883683 | C | T | 0.877 | 0.056 | 0.003 | 1.9E-67 |
| rs776715248 | 23 | 66920179 | C | T | 0.001 | 0.648 | 0.037 | 1.8E-73 |
| rs147676232 | 23 | 67932642 | C | T | 0.987 | 0.067 | 0.01 | 1.6E-11 |
| rs6616174 | 23 | 99946825 | G | A | 0.359 | 0.016 | 0.002 | 2E-12 |
| rs41306249 | 23 | 109697443 | T | C | 0.954 | 0.042 | 0.005 | 2E-16 |
| rs243466 | 23 | 131802339 | C | G | 0.296 | 0.013 | 0.002 | 4.8E-08 |
| rs757309 | 23 | 133680547 | A | G | 0.641 | 0.018 | 0.002 | 9.9E-16 |
| X:135998134_CA_C | 23 | 135998134 | CA | C | 0.512 | 0.017 | 0.002 | 1.1E-15 |
| rs5905042 | 23 | 146444527 | C | A | 0.691 | 0.023 | 0.002 | 4.3E-22 |
| rs10218066 | 23 | 150343248 | G | T | 0.785 | 0.015 | 0.003 | 2.5E-08 |

**Table S5** – Genome-wide significant single nucleotide polymorphisms (SNP) used as instrumental variants for total testosterone in males..

| **SNP** | **Variant chromosome** | **Variant position** | **Effect allele** | **Non-effect allele** | **Effect allele frequency** | **Beta** | **Standard error** | **P-value** |
| --- | --- | --- | --- | --- | --- | --- | --- | --- |
| rs36086195 | 1 | 16510894 | T | C | 0.579 | 0.019 | 0.003 | 1.70E-09 |
| rs66844552 | 1 | 25802056 | A | G | 0.598 | 0.017 | 0.003 | 3.30E-08 |
| rs114165349 | 1 | 27021913 | G | C | 0.977 | 0.148 | 0.01 | 6.70E-51 |
| rs3768321 | 1 | 40035928 | G | T | 0.803 | 0.031 | 0.004 | 5.40E-15 |
| rs9970140 | 1 | 61684288 | G | A | 0.076 | 0.04 | 0.006 | 6.80E-12 |
| rs141509569 | 1 | 65973220 | G | GTA | 0.381 | 0.02 | 0.003 | 2.90E-12 |
| rs6676846 | 1 | 92942352 | A | G | 0.794 | 0.03 | 0.004 | 1.20E-15 |
| rs12406721 | 1 | 1.08E+08 | T | G | 0.343 | 0.033 | 0.003 | 2.30E-28 |
| rs267733 | 1 | 1.51E+08 | A | G | 0.839 | 0.023 | 0.004 | 1.80E-08 |
| rs746303202 | 1 | 1.55E+08 | A | AG | 0.698 | 0.02 | 0.003 | 1.50E-10 |
| rs34702488 | 1 | 1.63E+08 | A | T | 0.176 | 0.026 | 0.004 | 1.70E-11 |
| 1:167851935_TGTAGTGAGAGGTAGCCCAGC_T | 1 | 1.68E+08 | T | TGTAGTGAGAGGTAGCCCAGC | 0.287 | 0.017 | 0.003 | 4.30E-08 |
| rs12125882 | 1 | 1.72E+08 | A | T | 0.579 | 0.017 | 0.003 | 3.70E-08 |
| rs35737316 | 1 | 2.04E+08 | T | C | 0.246 | 0.036 | 0.004 | 3.50E-27 |
| rs10864086 | 1 | 2.14E+08 | C | A | 0.256 | 0.022 | 0.003 | 5.30E-11 |
| 1:221045347_AT_A | 1 | 2.21E+08 | A | AT | 0.679 | 0.021 | 0.003 | 2.00E-11 |
| rs12470971 | 2 | 11725241 | A | G | 0.338 | 0.017 | 0.003 | 2.60E-08 |
| rs11096640 | 2 | 20354039 | C | A | 0.515 | 0.017 | 0.003 | 6.40E-10 |
| rs1260326 | 2 | 27730940 | C | T | 0.605 | 0.062 | 0.003 | 2.70E-91 |
| rs113017476 | 2 | 31989359 | A | G | 0.039 | 0.19 | 0.008 | 7.00E-134 |
| rs6736913 | 2 | 42510018 | A | G | 0.021 | 0.062 | 0.01 | 1.20E-08 |
| rs11433420 | 2 | 58037203 | T | TA | 0.359 | 0.019 | 0.003 | 1.40E-09 |
| rs12614829 | 2 | 64893183 | T | C | 0.207 | 0.03 | 0.004 | 6.10E-17 |
| rs2540945 | 2 | 65289825 | G | A | 0.363 | 0.018 | 0.003 | 8.00E-09 |
| rs6750410 | 2 | 70417730 | A | G | 0.935 | 0.042 | 0.006 | 5.20E-12 |
| rs10649609 | 2 | 1.66E+08 | AAAAAT | A | 0.37 | 0.017 | 0.003 | 3.50E-08 |
| rs10192634 | 2 | 1.81E+08 | T | C | 0.269 | 0.023 | 0.003 | 2.00E-11 |
| rs71027236 | 2 | 1.92E+08 | T | TTG | 0.775 | 0.02 | 0.004 | 9.70E-09 |
| rs2551641 | 2 | 2.08E+08 | C | T | 0.189 | 0.022 | 0.004 | 7.20E-09 |
| rs2012736 | 2 | 2.35E+08 | C | A | 0.918 | 0.048 | 0.006 | 5.10E-19 |
| rs34396520 | 3 | 12673823 | CA | C | 0.362 | 0.021 | 0.003 | 4.20E-11 |
| rs6792725 | 3 | 24520283 | G | A | 0.694 | 0.019 | 0.003 | 3.20E-09 |
| rs7610366 | 3 | 28810588 | T | C | 0.726 | 0.021 | 0.003 | 2.20E-11 |
| rs112765699 | 3 | 61130525 | A | G | 0.015 | 0.075 | 0.013 | 2.20E-08 |
| rs66956368 | 3 | 61279726 | T | A | 0.703 | 0.03 | 0.004 | 2.10E-19 |
| rs55869022 | 3 | 61656512 | C | G | 0.868 | 0.027 | 0.005 | 2.00E-09 |
| rs3841946 | 3 | 88105684 | C | CA | 0.157 | 0.033 | 0.004 | 8.60E-17 |
| rs34040779 | 3 | 1.07E+08 | T | C | 0.924 | 0.034 | 0.006 | 5.30E-09 |
| rs645040 | 3 | 1.36E+08 | G | T | 0.227 | 0.043 | 0.004 | 1.50E-35 |
| rs6766859 | 3 | 1.38E+08 | T | C | 0.628 | 0.031 | 0.003 | 1.00E-21 |
| 3:152118069_ATTTATAATATGCCACTTTAGT_A | 3 | 1.52E+08 | A | ATTTATAATATGCCACTTTAGT | 0.099 | 0.035 | 0.005 | 4.50E-13 |
| rs61762319 | 3 | 1.55E+08 | G | A | 0.029 | 0.061 | 0.009 | 1.40E-11 |
| rs59194935 | 3 | 1.72E+08 | A | G | 0.432 | 0.02 | 0.003 | 3.70E-11 |
| rs13074711 | 3 | 1.72E+08 | T | C | 0.884 | 0.028 | 0.005 | 3.00E-09 |
| rs13108218 | 4 | 3443931 | A | G | 0.383 | 0.035 | 0.003 | 1.90E-29 |
| rs1203109 | 4 | 3455362 | C | T | 0.227 | 0.024 | 0.004 | 2.00E-11 |
| rs7679843 | 4 | 22028079 | G | C | 0.095 | 0.043 | 0.005 | 1.20E-17 |
| 4:69447407_GT_G | 4 | 69447407 | GT | G | 0.322 | 0.06 | 0.004 | 1.10E-60 |
| rs7696472 | 4 | 69538180 | A | G | 0.476 | 0.038 | 0.003 | 1.50E-39 |
| rs10028954 | 4 | 70466280 | C | T | 0.585 | 0.019 | 0.003 | 2.60E-09 |
| rs1441911 | 4 | 77193545 | G | T | 0.783 | 0.022 | 0.004 | 5.90E-09 |
| rs17408832 | 4 | 87222696 | C | G | 0.622 | 0.019 | 0.003 | 7.90E-10 |
| rs11735092 | 4 | 88226231 | T | C | 0.562 | 0.037 | 0.003 | 9.30E-35 |
| rs1154401 | 4 | 1E+08 | G | C | 0.338 | 0.028 | 0.003 | 2.90E-18 |
| rs201814405 | 4 | 1.04E+08 | A | T | 0.152 | 0.034 | 0.005 | 3.50E-13 |
| rs565931739 | 4 | 1.05E+08 | C | CA | 0.836 | 0.039 | 0.004 | 3.20E-20 |
| rs528845403 | 4 | 1.05E+08 | A | AATGTGT | 0.99 | 0.199 | 0.016 | 3.70E-34 |
| rs114816312 | 4 | 1.11E+08 | T | C | 0.007 | 0.119 | 0.018 | 2.00E-11 |
| rs11099675 | 4 | 1.49E+08 | T | C | 0.248 | 0.021 | 0.004 | 8.80E-10 |
| rs60701 | 5 | 10733776 | C | T | 0.257 | 0.019 | 0.003 | 4.30E-08 |
| rs7735249 | 5 | 53310139 | C | G | 0.887 | 0.028 | 0.005 | 1.80E-09 |
| rs40270 | 5 | 55804552 | A | C | 0.228 | 0.022 | 0.004 | 2.40E-09 |
| rs112530420 | 5 | 95871370 | C | T | 0.166 | 0.026 | 0.004 | 5.30E-10 |
| rs329122 | 5 | 1.34E+08 | G | A | 0.581 | 0.018 | 0.003 | 2.90E-09 |
| 5:135639923_GA_G | 5 | 1.36E+08 | GA | G | 0.865 | 0.031 | 0.004 | 8.20E-11 |
| rs6870458 | 5 | 1.38E+08 | T | G | 0.549 | 0.023 | 0.003 | 4.10E-13 |
| rs1349359 | 5 | 1.66E+08 | A | G | 0.438 | 0.017 | 0.003 | 2.10E-08 |
| rs9461224 | 6 | 25936402 | T | G | 0.397 | 0.018 | 0.003 | 4.00E-10 |
| rs2016572 | 6 | 31458983 | G | A | 0.332 | 0.026 | 0.003 | 5.80E-12 |
| rs543504257 | 6 | 32571403 | A | C | 0.506 | 0.024 | 0.003 | 1.30E-11 |
| rs6939861 | 6 | 41703041 | G | A | 0.738 | 0.025 | 0.003 | 1.70E-12 |
| rs62415384 | 6 | 43261767 | G | C | 0.548 | 0.016 | 0.003 | 2.60E-08 |
| 6:100108564_TG_T | 6 | 1E+08 | T | TG | 0.454 | 0.022 | 0.003 | 4.30E-14 |
| rs1933801 | 6 | 1.05E+08 | T | C | 0.678 | 0.038 | 0.003 | 7.80E-33 |
| 6:131936324_GTTT_G | 6 | 1.32E+08 | GTTT | G | 0.834 | 0.026 | 0.004 | 2.20E-11 |
| rs9383605 | 6 | 1.52E+08 | A | T | 0.705 | 0.019 | 0.003 | 2.40E-09 |
| rs7773995 | 6 | 1.54E+08 | C | T | 0.831 | 0.024 | 0.004 | 1.20E-09 |
| 6:160768849_CTGCCCTG_C | 6 | 1.61E+08 | C | CTGCCCTG | 0.467 | 0.02 | 0.003 | 8.60E-14 |
| rs9986829 | 7 | 15019259 | A | G | 0.507 | 0.041 | 0.003 | 5.20E-45 |
| rs1708302 | 7 | 28198677 | T | C | 0.502 | 0.018 | 0.003 | 2.60E-09 |
| rs10279715 | 7 | 40870935 | A | G | 0.538 | 0.018 | 0.003 | 9.90E-10 |
| 7:78482336_AG_A | 7 | 78482336 | AG | A | 0.964 | 0.052 | 0.009 | 2.50E-10 |
| rs1229498 | 7 | 81568750 | T | G | 0.278 | 0.019 | 0.003 | 6.50E-09 |
| rs7015 | 7 | 97920623 | G | A | 0.815 | 0.056 | 0.004 | 7.60E-49 |
| rs11772470 | 7 | 99181096 | A | G | 0.148 | 0.03 | 0.004 | 7.70E-14 |
| 7:150502468_AT_A | 7 | 1.51E+08 | A | AT | 0.111 | 0.033 | 0.005 | 8.10E-14 |
| rs4841133 | 8 | 9183664 | G | A | 0.909 | 0.031 | 0.005 | 8.90E-10 |
| rs7835492 | 8 | 21089517 | C | T | 0.147 | 0.025 | 0.004 | 1.10E-09 |
| rs4871844 | 8 | 22879734 | C | T | 0.344 | 0.017 | 0.003 | 3.10E-08 |
| rs10958704 | 8 | 38328302 | A | G | 0.598 | 0.02 | 0.003 | 2.70E-11 |
| rs12543287 | 8 | 42334511 | C | G | 0.371 | 0.02 | 0.003 | 9.80E-11 |
| rs7844586 | 8 | 61782304 | C | T | 0.751 | 0.02 | 0.004 | 1.10E-08 |
| rs55867305 | 8 | 77884459 | G | A | 0.749 | 0.033 | 0.003 | 1.80E-21 |
| rs7824394 | 8 | 81292599 | C | A | 0.641 | 0.024 | 0.003 | 1.20E-13 |
| rs76767219 | 8 | 81426196 | A | C | 0.035 | 0.055 | 0.008 | 2.10E-12 |
| rs34955534 | 8 | 81710349 | G | A | 0.9 | 0.042 | 0.005 | 4.10E-16 |
| rs7828742 | 8 | 1.17E+08 | A | G | 0.4 | 0.017 | 0.003 | 2.50E-08 |
| rs148204274 | 8 | 1.24E+08 | TGTGTCTGTACA | T | 0.343 | 0.016 | 0.003 | 4.00E-08 |
| rs2721195 | 8 | 1.46E+08 | T | C | 0.475 | 0.02 | 0.003 | 7.50E-11 |
| rs12336359 | 9 | 4129657 | C | G | 0.406 | 0.019 | 0.003 | 1.70E-09 |
| rs112107457 | 9 | 19103774 | T | C | 0.132 | 0.029 | 0.005 | 1.00E-10 |
| rs3808869 | 9 | 34622389 | C | A | 0.521 | 0.017 | 0.003 | 5.40E-09 |
| rs148262335 | 9 | 77222516 | TTA | T | 0.339 | 0.026 | 0.003 | 1.50E-17 |
| rs199950405 | 9 | 83271419 | C | G | 0.259 | 0.019 | 0.004 | 4.20E-08 |
| rs10868080 | 9 | 86626769 | T | A | 0.255 | 0.036 | 0.003 | 1.20E-27 |
| rs41310053 | 9 | 88940359 | T | C | 0.006 | 0.112 | 0.02 | 3.90E-08 |
| 9:97023094_ATTTTTTTTTTTT_A | 9 | 97023094 | A | ATTTTTTTTTTTT | 0.484 | 0.016 | 0.003 | 3.10E-08 |
| rs2090409 | 9 | 1.09E+08 | C | A | 0.684 | 0.026 | 0.003 | 2.20E-16 |
| rs10982156 | 9 | 1.17E+08 | A | T | 0.07 | 0.037 | 0.006 | 1.40E-09 |
| rs10982192 | 9 | 1.17E+08 | T | C | 0.222 | 0.024 | 0.004 | 6.30E-11 |
| rs13289095 | 9 | 1.31E+08 | G | T | 0.855 | 0.021 | 0.004 | 4.00E-08 |
| rs35182096 | 9 | 1.37E+08 | C | T | 0.256 | 0.023 | 0.004 | 1.30E-10 |
| 9:140774721_T_C | 9 | 1.41E+08 | C | T | 0.489 | 0.017 | 0.003 | 1.90E-08 |
| rs79717793 | 10 | 5262267 | G | A | 0.845 | 0.049 | 0.004 | 9.10E-36 |
| rs77044968 | 10 | 63657706 | C | T | 0.061 | 0.037 | 0.006 | 2.30E-09 |
| 10:65158772_AAAG_A | 10 | 65158772 | A | AAAG | 0.472 | 0.11 | 0.003 | 1.10E-304 |
| rs34131245 | 10 | 66174554 | C | A | 0.906 | 0.03 | 0.005 | 3.30E-08 |
| rs7912521 | 10 | 67262089 | C | T | 0.415 | 0.049 | 0.003 | 2.60E-61 |
| 10:69632729_AT_A | 10 | 69632729 | AT | A | 0.345 | 0.024 | 0.003 | 1.80E-13 |
| 10:81073027_CCCACCCACCA_C | 10 | 81073027 | CCCACCCACCA | C | 0.784 | 0.021 | 0.004 | 2.40E-08 |
| 10:93564864_CT_C | 10 | 93564864 | CT | C | 0.515 | 0.02 | 0.003 | 8.80E-11 |
| rs2862954 | 10 | 1.02E+08 | C | T | 0.5 | 0.018 | 0.003 | 5.80E-09 |
| rs5787948 | 10 | 1.14E+08 | C | CA | 0.267 | 0.018 | 0.003 | 2.00E-08 |
| rs7915430 | 10 | 1.22E+08 | T | G | 0.798 | 0.025 | 0.004 | 2.30E-12 |
| rs775181992 | 11 | 2179864 | A | AGCCCT | 0.24 | 0.023 | 0.004 | 1.40E-11 |
| rs2957683 | 11 | 10352175 | C | G | 0.528 | 0.016 | 0.003 | 5.90E-09 |
| rs4757142 | 11 | 13325695 | G | A | 0.391 | 0.018 | 0.003 | 6.40E-09 |
| rs10832570 | 11 | 16249510 | A | G | 0.612 | 0.025 | 0.003 | 1.80E-16 |
| rs1994721 | 11 | 29204531 | G | A | 0.852 | 0.049 | 0.004 | 1.90E-32 |
| rs145843487 | 11 | 29323066 | G | C | 0.981 | 0.076 | 0.011 | 3.90E-12 |
| rs200647710 | 11 | 32786939 | A | AAC | 0.471 | 0.016 | 0.003 | 2.00E-08 |
| rs11607114 | 11 | 48151287 | C | G | 0.844 | 0.025 | 0.004 | 4.20E-10 |
| rs10750766 | 11 | 65473798 | C | A | 0.289 | 0.022 | 0.003 | 1.60E-11 |
| rs631695 | 11 | 69283303 | T | G | 0.417 | 0.024 | 0.003 | 7.20E-17 |
| rs12787293 | 11 | 72394147 | G | A | 0.458 | 0.02 | 0.003 | 8.00E-11 |
| rs12796488 | 11 | 94131557 | C | A | 0.823 | 0.041 | 0.004 | 2.00E-26 |
| rs4754839 | 11 | 1.02E+08 | A | G | 0.562 | 0.018 | 0.003 | 1.10E-08 |
| rs10892924 | 11 | 1.23E+08 | T | A | 0.568 | 0.032 | 0.003 | 7.60E-26 |
| rs618888 | 11 | 1.25E+08 | T | G | 0.283 | 0.023 | 0.003 | 1.30E-12 |
| rs56196860 | 12 | 2908330 | A | C | 0.032 | 0.301 | 0.009 | 5.10E-276 |
| rs61922185 | 12 | 21039679 | T | G | 0.321 | 0.021 | 0.003 | 1.80E-11 |
| rs73079476 | 12 | 21343833 | A | C | 0.849 | 0.054 | 0.004 | 4.20E-40 |
| rs12320328 | 12 | 25408464 | A | G | 0.915 | 0.043 | 0.005 | 2.30E-16 |
| rs111538456 | 12 | 51042486 | C | CT | 0.651 | 0.026 | 0.003 | 1.50E-17 |
| rs540730 | 12 | 57807114 | T | C | 0.245 | 0.03 | 0.004 | 3.70E-19 |
| rs2583948 | 12 | 66194613 | A | G | 0.903 | 0.035 | 0.005 | 3.00E-11 |
| rs191591035 | 12 | 99868285 | G | C | 0.997 | 0.222 | 0.035 | 2.50E-10 |
| rs61755050 | 12 | 1.01E+08 | T | C | 0.994 | 0.211 | 0.02 | 1.30E-30 |
| rs7314285 | 12 | 1.12E+08 | G | T | 0.068 | 0.039 | 0.006 | 8.80E-12 |
| rs3809272 | 12 | 1.12E+08 | G | A | 0.697 | 0.02 | 0.003 | 3.90E-10 |
| rs12810788 | 12 | 1.16E+08 | G | A | 0.201 | 0.025 | 0.004 | 1.50E-10 |
| rs2393775 | 12 | 1.21E+08 | A | G | 0.623 | 0.026 | 0.003 | 2.70E-17 |
| rs534541609 | 12 | 1.25E+08 | C | CACCT | 0.742 | 0.022 | 0.004 | 3.20E-09 |
| rs6486542 | 12 | 1.31E+08 | C | T | 0.57 | 0.021 | 0.003 | 1.30E-11 |
| rs7997628 | 13 | 95217852 | A | T | 0.35 | 0.017 | 0.003 | 2.80E-08 |
| rs2038695 | 13 | 1.01E+08 | C | A | 0.449 | 0.021 | 0.003 | 1.10E-12 |
| rs3742223 | 13 | 1.13E+08 | T | C | 0.904 | 0.03 | 0.005 | 2.80E-10 |
| rs139713000 | 14 | 23731855 | CT | C | 0.196 | 0.028 | 0.004 | 1.30E-15 |
| rs11621792 | 14 | 24871926 | C | T | 0.546 | 0.018 | 0.003 | 6.70E-09 |
| rs72681869 | 14 | 50655357 | C | G | 0.011 | 0.103 | 0.015 | 3.70E-14 |
| rs140218129 | 14 | 61094365 | T | TAA | 0.139 | 0.025 | 0.005 | 3.80E-08 |
| rs2239222 | 14 | 73011885 | G | A | 0.349 | 0.021 | 0.003 | 1.80E-10 |
| rs72721770 | 14 | 74204686 | G | C | 0.652 | 0.019 | 0.003 | 1.00E-09 |
| rs1812755 | 14 | 90007637 | T | C | 0.8 | 0.026 | 0.004 | 1.50E-11 |
| rs145602600 | 14 | 94838202 | T | C | 0.005 | 0.133 | 0.021 | 8.20E-11 |
| rs28929474 | 14 | 94844947 | T | C | 0.02 | 0.222 | 0.011 | 1.20E-95 |
| rs17580 | 14 | 94847262 | A | T | 0.048 | 0.049 | 0.007 | 1.60E-12 |
| rs7143218 | 14 | 1.01E+08 | C | A | 0.269 | 0.025 | 0.003 | 4.90E-14 |
| rs45490496 | 14 | 1.05E+08 | A | T | 0.387 | 0.021 | 0.003 | 2.60E-11 |
| rs55707100 | 15 | 43820717 | C | T | 0.975 | 0.111 | 0.01 | 1.80E-33 |
| rs28892005 | 15 | 51519945 | A | AAAG | 0.351 | 0.028 | 0.003 | 2.40E-19 |
| rs140357247 | 15 | 52734389 | C | G | 0.998 | 0.241 | 0.038 | 7.20E-10 |
| rs79391862 | 15 | 53739426 | A | C | 0.986 | 0.157 | 0.013 | 5.60E-38 |
| rs12910403 | 15 | 57073464 | G | C | 0.634 | 0.018 | 0.003 | 2.30E-08 |
| rs200651966 | 15 | 63828891 | C | CA | 0.81 | 0.033 | 0.004 | 5.00E-18 |
| rs7182912 | 15 | 89038812 | G | T | 0.809 | 0.021 | 0.004 | 3.90E-09 |
| rs7166920 | 15 | 96219503 | A | G | 0.501 | 0.02 | 0.003 | 1.10E-10 |
| rs56332871 | 15 | 96714816 | A | C | 0.272 | 0.047 | 0.003 | 1.70E-47 |
| 16:1684027_CA_C | 16 | 1684027 | C | CA | 0.102 | 0.029 | 0.005 | 4.50E-08 |
| rs841194 | 16 | 4667690 | G | A | 0.822 | 0.027 | 0.004 | 6.70E-11 |
| rs12445820 | 16 | 11866703 | G | C | 0.148 | 0.025 | 0.004 | 8.00E-09 |
| rs2764772 | 16 | 20060653 | A | T | 0.334 | 0.032 | 0.003 | 2.50E-24 |
| 16:28704506_ATTT_A | 16 | 28704506 | ATTT | A | 0.598 | 0.034 | 0.003 | 1.30E-28 |
| rs1421085 | 16 | 53800954 | T | C | 0.595 | 0.022 | 0.003 | 1.20E-11 |
| rs42945 | 16 | 58545426 | A | G | 0.488 | 0.016 | 0.003 | 4.30E-08 |
| rs57349855 | 16 | 79709572 | C | CT | 0.369 | 0.022 | 0.003 | 5.20E-12 |
| rs79488654 | 16 | 82131688 | G | T | 0.999 | 0.235 | 0.041 | 7.90E-09 |
| rs4782568 | 16 | 83980529 | G | C | 0.452 | 0.025 | 0.003 | 7.90E-17 |
| rs12926107 | 16 | 88004092 | A | G | 0.542 | 0.024 | 0.003 | 7.10E-16 |
| rs550628400 | 17 | 1639795 | G | A | 0.006 | 0.134 | 0.02 | 8.60E-11 |
| rs4525526 | 17 | 1650125 | C | T | 0.219 | 0.033 | 0.004 | 2.50E-20 |
| rs1799941 | 17 | 7533423 | A | G | 0.261 | 0.197 | 0.003 | 9.1E-742 |
| rs6258 | 17 | 7534678 | C | T | 0.993 | 0.718 | 0.018 | 6.4E-347 |
| 17:17917246_AT_A | 17 | 17917246 | AT | A | 0.625 | 0.019 | 0.003 | 9.60E-10 |
| rs56853305 | 17 | 27648542 | G | A | 0.859 | 0.027 | 0.004 | 4.40E-10 |
| rs2905801 | 17 | 29524974 | T | C | 0.706 | 0.029 | 0.003 | 3.60E-19 |
| rs650558 | 17 | 40721042 | C | T | 0.752 | 0.022 | 0.003 | 1.60E-10 |
| rs62062271 | 17 | 44091988 | C | T | 0.228 | 0.031 | 0.004 | 5.40E-18 |
| rs576009056 | 17 | 46408186 | G | GA | 0.765 | 0.023 | 0.004 | 1.00E-10 |
| rs28394864 | 17 | 47450775 | G | A | 0.539 | 0.053 | 0.003 | 1.50E-72 |
| rs2306216 | 17 | 73240559 | A | G | 0.168 | 0.027 | 0.004 | 9.20E-12 |
| rs7216664 | 17 | 73825664 | G | A | 0.362 | 0.019 | 0.003 | 2.40E-10 |
| rs62076019 | 17 | 79481003 | G | T | 0.601 | 0.016 | 0.003 | 4.10E-08 |
| rs2668776 | 18 | 44750365 | C | T | 0.469 | 0.023 | 0.003 | 6.80E-14 |
| rs1624295 | 19 | 2792034 | A | G | 0.293 | 0.033 | 0.003 | 7.20E-24 |
| rs8107967 | 19 | 7972615 | G | A | 0.568 | 0.019 | 0.003 | 1.70E-10 |
| rs10421262 | 19 | 14172951 | T | G | 0.576 | 0.021 | 0.003 | 1.70E-12 |
| rs202200760 | 19 | 17346854 | C | G | 0.039 | 0.12 | 0.009 | 8.60E-48 |
| rs35824797 | 19 | 19456264 | C | T | 0.921 | 0.044 | 0.006 | 1.70E-15 |
| 19:35555222_GTC_G | 19 | 35555222 | G | GTC | 0.86 | 0.037 | 0.004 | 4.50E-18 |
| rs34851490 | 19 | 46384554 | G | A | 0.116 | 0.048 | 0.005 | 1.80E-23 |
| rs11671304 | 19 | 47564643 | C | T | 0.329 | 0.016 | 0.003 | 4.40E-08 |
| rs573396813 | 19 | 49487651 | CT | C | 0.558 | 0.018 | 0.003 | 2.90E-08 |
| rs139316391 | 19 | 50016971 | GTCTCTCTCTC | G | 0.077 | 0.053 | 0.006 | 1.60E-20 |
| 20:39951474_TA_T | 20 | 39951474 | T | TA | 0.4 | 0.022 | 0.003 | 1.30E-14 |
| rs6073431 | 20 | 43040569 | T | C | 0.531 | 0.031 | 0.003 | 5.00E-24 |
| rs1058319 | 20 | 62374389 | T | C | 0.135 | 0.024 | 0.005 | 1.50E-08 |
| rs575146 | 22 | 24295074 | G | A | 0.398 | 0.018 | 0.003 | 1.10E-08 |
| rs1033667 | 22 | 29130300 | T | C | 0.3 | 0.023 | 0.003 | 9.30E-13 |
| rs738409 | 22 | 44324727 | G | C | 0.216 | 0.051 | 0.004 | 4.30E-43 |
| rs11703376 | 22 | 49678713 | T | C | 0.27 | 0.034 | 0.003 | 2.70E-23 |
| rs112265145 | 23 | 8906893 | C | A | 0.267 | 0.098 | 0.002 | 1.1E-372 |
| rs782159242 | 23 | 50220830 | CT | C | 0.705 | 0.014 | 0.002 | 2.20E-09 |
| X:54243748_CA_C | 23 | 54243748 | CA | C | 0.693 | 0.016 | 0.003 | 1.40E-10 |
| rs761954625 | 23 | 56786833 | CTG | C | 0.227 | 0.032 | 0.003 | 4.60E-38 |
| rs146447930 | 23 | 63167507 | C | T | 0.986 | 0.071 | 0.009 | 2.50E-16 |
| rs74805556 | 23 | 65779640 | T | C | 0.724 | 0.04 | 0.002 | 1.40E-60 |
| rs72630041 | 23 | 71399338 | G | A | 0.192 | 0.018 | 0.003 | 1.90E-11 |
| rs881090 | 23 | 1.1E+08 | T | G | 0.391 | 0.063 | 0.002 | 5.90E-188 |
| rs7065171 | 23 | 1.34E+08 | G | C | 0.654 | 0.018 | 0.002 | 1.70E-16 |
| X:146434326_GTTT_G | 23 | 1.46E+08 | GTTT | G | 0.56 | 0.017 | 0.002 | 6.50E-15 |

**Table S6** – Genome-wide significant single nucleotide polymorphisms (SNP) used as instrumental variants for sex hormone binding globulin in males.

| **SNP** | **Variant chromosome** | **Variant position** | **Effect allele** | **Non-effect allele** | **Effect allele frequency** | **Beta** | **Standard error** | **P-value** |
| --- | --- | --- | --- | --- | --- | --- | --- | --- |
| **1:1510035_GGC_G** | **1** | **1510035** | **G** | **GGC** | **0.288** | **0.009** | **0.001** | **3.30E-12** |
| **rs4333851** | **1** | **10146179** | **A** | **G** | **0.154** | **0.008** | **0.002** | **1.30E-08** |
| **rs198384** | **1** | **11934378** | **A** | **G** | **0.551** | **0.007** | **0.001** | **2.20E-08** |
| **rs36086195** | **1** | **16510894** | **T** | **C** | **0.579** | **0.017** | **0.001** | **2.30E-52** |
| **1:23747996_GA_G** | **1** | **23747996** | **G** | **GA** | **0.727** | **0.007** | **0.001** | **4.80E-11** |
| **1:25827633_CT_C** | **1** | **25827633** | **CT** | **C** | **0.55** | **0.01** | **0.001** | **1.90E-20** |
| **rs114165349** | **1** | **27021913** | **G** | **C** | **0.977** | **0.09** | **0.004** | **1.10E-135** |
| **rs565471584** | **1** | **28022862** | **T** | **C** | **0.996** | **0.078** | **0.011** | **3.60E-12** |
| **1:35734986_AAGTGCATCTTT_A** | **1** | **35734986** | **A** | **AAGTGCATCTTT** | **0.908** | **0.011** | **0.002** | **1.10E-09** |
| **rs771435780** | **1** | **39859518** | **G** | **GGA** | **0.717** | **0.011** | **0.001** | **3.10E-20** |
| **rs141811210** | **1** | **51080191** | **GTAA** | **G** | **0.43** | **0.006** | **0.001** | **5.10E-09** |
| **rs1883783** | **1** | **54890956** | **T** | **G** | **0.43** | **0.006** | **0.001** | **3.20E-08** |
| **rs59708846** | **1** | **61687651** | **A** | **G** | **0.076** | **0.019** | **0.002** | **1.30E-20** |
| **rs35067979** | **1** | **61925076** | **C** | **T** | **0.929** | **0.016** | **0.002** | **1.00E-11** |
| **1:93033928_CT_C** | **1** | **93033928** | **C** | **CT** | **0.79** | **0.011** | **0.001** | **7.00E-14** |
| **rs35526088** | **1** | **1.02E+08** | **T** | **G** | **0.386** | **0.007** | **0.001** | **2.70E-08** |
| **rs1730865** | **1** | **1.08E+08** | **G** | **T** | **0.345** | **0.028** | **0.001** | **8.00E-127** |
| **rs140584594** | **1** | **1.1E+08** | **A** | **G** | **0.269** | **0.012** | **0.001** | **2.20E-21** |
| **rs71584764** | **1** | **1.13E+08** | **TA** | **T** | **0.204** | **0.008** | **0.001** | **1.30E-08** |
| **rs72708162** | **1** | **1.5E+08** | **T** | **G** | **0.079** | **0.015** | **0.002** | **5.70E-14** |
| **rs140386498** | **1** | **1.51E+08** | **T** | **A** | **0.013** | **0.034** | **0.005** | **4.30E-12** |
| **rs71586027** | **1** | **1.55E+08** | **TATCTC** | **T** | **0.48** | **0.011** | **0.001** | **1.50E-22** |
| **rs768651403** | **1** | **1.56E+08** | **GT** | **G** | **0.759** | **0.01** | **0.001** | **3.00E-14** |
| **rs2275560** | **1** | **1.65E+08** | **G** | **A** | **0.744** | **0.008** | **0.001** | **9.80E-09** |
| **1:167851935_TGTAGTGAGAGGTAGCCCAGC_T** | **1** | **1.68E+08** | **T** | **TGTAGTGAGAGGTAGCCCAGC** | **0.287** | **0.007** | **0.001** | **5.90E-09** |
| **rs12059956** | **1** | **1.71E+08** | **G** | **A** | **0.583** | **0.007** | **0.001** | **3.40E-10** |
| **rs78444298** | **1** | **1.85E+08** | **G** | **A** | **0.981** | **0.024** | **0.004** | **3.10E-11** |
| **1:196914907_AGT_A** | **1** | **1.97E+08** | **AGT** | **A** | **0.763** | **0.009** | **0.001** | **2.20E-11** |
| **rs17583875** | **1** | **1.98E+08** | **A** | **G** | **0.021** | **0.023** | **0.004** | **1.10E-09** |
| **rs7540115** | **1** | **2E+08** | **C** | **A** | **0.819** | **0.01** | **0.001** | **2.90E-11** |
| **rs148911629** | **1** | **2.14E+08** | **G** | **C** | **0.991** | **0.043** | **0.006** | **6.10E-14** |
| **rs10864086** | **1** | **2.14E+08** | **C** | **A** | **0.256** | **0.015** | **0.001** | **2.90E-32** |
| **rs2820441** | **1** | **2.2E+08** | **C** | **A** | **0.318** | **0.009** | **0.001** | **2.70E-13** |
| **rs2247213** | **1** | **2.21E+08** | **G** | **A** | **0.67** | **0.015** | **0.001** | **1.30E-36** |
| **rs1870927** | **1** | **2.26E+08** | **A** | **T** | **0.62** | **0.007** | **0.001** | **2.30E-08** |
| **rs144647926** | **1** | **2.35E+08** | **A** | **G** | **0.087** | **0.013** | **0.002** | **1.30E-10** |
| **rs907866** | **2** | **20371380** | **G** | **A** | **0.555** | **0.008** | **0.001** | **1.30E-15** |
| **rs1260326** | **2** | **27730940** | **C** | **T** | **0.606** | **0.041** | **0.001** | **1.30E-298** |
| **rs138529890** | **2** | **32478354** | **A** | **G** | **0.038** | **0.024** | **0.003** | **1.10E-14** |
| **rs6736913** | **2** | **42510018** | **A** | **G** | **0.021** | **0.03** | **0.004** | **1.50E-14** |
| **rs200883214** | **2** | **48725415** | **G** | **GT** | **0.443** | **0.006** | **0.001** | **2.10E-09** |
| **rs10657979** | **2** | **64907677** | **C** | **CCTT** | **0.264** | **0.016** | **0.001** | **5.90E-39** |
| **rs6750410** | **2** | **70417730** | **A** | **G** | **0.935** | **0.018** | **0.002** | **4.80E-16** |
| **2:111925731_CTTATGTT_C** | **2** | **1.12E+08** | **CTTATGTT** | **C** | **0.937** | **0.017** | **0.002** | **9.00E-14** |
| **rs3747647** | **2** | **1.12E+08** | **C** | **G** | **0.218** | **0.009** | **0.001** | **4.00E-10** |
| **rs2197771** | **2** | **1.12E+08** | **G** | **T** | **0.369** | **0.007** | **0.001** | **2.50E-09** |
| **rs17050272** | **2** | **1.21E+08** | **A** | **G** | **0.41** | **0.007** | **0.001** | **1.40E-10** |
| **rs142740991** | **2** | **1.49E+08** | **C** | **CA** | **0.29** | **0.008** | **0.001** | **1.70E-10** |
| **rs13389219** | **2** | **1.66E+08** | **T** | **C** | **0.394** | **0.012** | **0.001** | **2.30E-26** |
| **rs72948115** | **2** | **1.78E+08** | **C** | **T** | **0.905** | **0.012** | **0.002** | **2.20E-10** |
| **rs8176526** | **2** | **1.88E+08** | **C** | **T** | **0.73** | **0.008** | **0.001** | **1.60E-10** |
| **2:191559843_GT_G** | **2** | **1.92E+08** | **GT** | **G** | **0.772** | **0.01** | **0.001** | **1.60E-13** |
| **rs4675682** | **2** | **2.08E+08** | **T** | **C** | **0.54** | **0.009** | **0.001** | **9.20E-16** |
| **rs4673528** | **2** | **2.11E+08** | **C** | **T** | **0.556** | **0.005** | **0.001** | **4.20E-08** |
| **rs62182125** | **2** | **2.19E+08** | **G** | **A** | **0.449** | **0.008** | **0.001** | **1.20E-12** |
| **rs12694450** | **2** | **2.2E+08** | **T** | **C** | **0.325** | **0.007** | **0.001** | **3.00E-10** |
| **rs2222018** | **2** | **2.27E+08** | **C** | **A** | **0.352** | **0.014** | **0.001** | **5.10E-31** |
| **rs62195072** | **2** | **2.34E+08** | **C** | **T** | **0.325** | **0.009** | **0.001** | **6.30E-14** |
| **rs10153800** | **2** | **2.42E+08** | **A** | **G** | **0.23** | **0.007** | **0.001** | **2.90E-08** |
| **rs17036326** | **3** | **12389313** | **G** | **A** | **0.122** | **0.019** | **0.002** | **1.20E-26** |
| **rs6792725** | **3** | **24520283** | **G** | **A** | **0.694** | **0.013** | **0.001** | **4.40E-26** |
| **rs762450647** | **3** | **47125916** | **CT** | **C** | **0.466** | **0.007** | **0.001** | **6.50E-09** |
| **3:49562992_CT_C** | **3** | **49562992** | **CT** | **C** | **0.534** | **0.008** | **0.001** | **2.90E-12** |
| **rs2564923** | **3** | **53103262** | **A** | **G** | **0.443** | **0.006** | **0.001** | **8.10E-11** |
| **rs71115649** | **3** | **69142923** | **A** | **AC** | **0.346** | **0.008** | **0.001** | **9.20E-11** |
| **rs13315174** | **3** | **1.05E+08** | **G** | **A** | **0.784** | **0.008** | **0.001** | **1.50E-10** |
| **rs60005573** | **3** | **1.29E+08** | **GGATTAT** | **G** | **0.078** | **0.011** | **0.002** | **1.40E-08** |
| **rs687339** | **3** | **1.36E+08** | **C** | **T** | **0.228** | **0.025** | **0.001** | **5.30E-84** |
| **rs7623513** | **3** | **1.42E+08** | **C** | **A** | **0.129** | **0.01** | **0.002** | **6.70E-09** |
| **rs12696304** | **3** | **1.69E+08** | **G** | **C** | **0.266** | **0.008** | **0.001** | **9.90E-10** |
| **rs79287178** | **3** | **1.72E+08** | **G** | **A** | **0.968** | **0.041** | **0.003** | **9.60E-38** |
| **rs234051** | **3** | **1.72E+08** | **A** | **G** | **0.284** | **0.009** | **0.001** | **1.80E-15** |
| **rs7631981** | **3** | **1.85E+08** | **G** | **A** | **0.697** | **0.008** | **0.001** | **6.50E-13** |
| **rs35654957** | **4** | **1010077** | **T** | **C** | **0.63** | **0.008** | **0.001** | **5.10E-13** |
| **rs13108218** | **4** | **3443931** | **A** | **G** | **0.383** | **0.023** | **0.001** | **3.70E-96** |
| **rs11734408** | **4** | **23882519** | **G** | **A** | **0.293** | **0.008** | **0.001** | **2.10E-13** |
| **rs7438888** | **4** | **69474676** | **C** | **T** | **0.005** | **0.069** | **0.008** | **3.00E-18** |
| **rs4860987** | **4** | **69491284** | **T** | **A** | **0.26** | **0.029** | **0.001** | **1.30E-105** |
| **rs1349852** | **4** | **69533217** | **C** | **A** | **0.475** | **0.019** | **0.001** | **5.30E-69** |
| **rs28507491** | **4** | **77197651** | **A** | **G** | **0.376** | **0.014** | **0.001** | **2.00E-31** |
| **rs72663907** | **4** | **87185271** | **T** | **G** | **0.878** | **0.012** | **0.002** | **9.70E-12** |
| **rs7694379** | **4** | **88186509** | **G** | **A** | **0.567** | **0.02** | **0.001** | **5.50E-71** |
| **rs149675684** | **4** | **1E+08** | **G** | **A** | **0.003** | **0.085** | **0.012** | **2.90E-12** |
| **rs6831352** | **4** | **1E+08** | **T** | **C** | **0.302** | **0.02** | **0.001** | **2.90E-58** |
| **rs187325356** | **4** | **1.1E+08** | **A** | **T** | **0.008** | **0.066** | **0.007** | **7.50E-21** |
| **rs7655064** | **4** | **1.2E+08** | **T** | **C** | **0.875** | **0.008** | **0.002** | **4.00E-08** |
| **4:128987952_ATT_A** | **4** | **1.29E+08** | **A** | **ATT** | **0.607** | **0.007** | **0.001** | **8.30E-10** |
| **rs10027275** | **4** | **1.49E+08** | **G** | **C** | **0.259** | **0.013** | **0.001** | **3.90E-26** |
| **rs72729610** | **4** | **1.54E+08** | **A** | **G** | **0.834** | **0.009** | **0.002** | **9.10E-11** |
| **rs36103835** | **4** | **1.58E+08** | **T** | **C** | **0.249** | **0.008** | **0.001** | **1.50E-10** |
| **rs78890745** | **4** | **1.6E+08** | **A** | **G** | **0.109** | **0.014** | **0.002** | **6.20E-14** |
| **rs11732763** | **4** | **1.71E+08** | **A** | **G** | **0.106** | **0.011** | **0.002** | **1.90E-10** |
| **rs29681** | **5** | **190639** | **T** | **C** | **0.877** | **0.009** | **0.002** | **4.00E-08** |
| **rs10069690** | **5** | **1279790** | **C** | **T** | **0.742** | **0.007** | **0.001** | **3.60E-08** |
| **5:36206855_GAT_G** | **5** | **36206855** | **G** | **GAT** | **0.023** | **0.022** | **0.004** | **1.10E-09** |
| **rs7735249** | **5** | **53310139** | **C** | **G** | **0.887** | **0.02** | **0.002** | **4.90E-31** |
| **rs40270** | **5** | **55804552** | **A** | **C** | **0.228** | **0.014** | **0.001** | **4.00E-26** |
| **rs79354983** | **5** | **56221537** | **A** | **G** | **0.905** | **0.016** | **0.002** | **2.50E-21** |
| **rs2216707** | **5** | **61951416** | **C** | **T** | **0.829** | **0.008** | **0.002** | **9.90E-11** |
| **rs34651** | **5** | **72144005** | **T** | **C** | **0.919** | **0.012** | **0.002** | **6.50E-09** |
| **rs11739158** | **5** | **72927292** | **T** | **C** | **0.428** | **0.008** | **0.001** | **1.70E-13** |
| **rs6595447** | **5** | **1.23E+08** | **T** | **C** | **0.807** | **0.01** | **0.001** | **1.20E-12** |
| **rs10649697** | **5** | **1.27E+08** | **T** | **TAGA** | **0.244** | **0.011** | **0.001** | **1.80E-18** |
| **rs2106854** | **5** | **1.32E+08** | **T** | **C** | **0.187** | **0.009** | **0.001** | **1.40E-09** |
| **rs329122** | **5** | **1.34E+08** | **G** | **A** | **0.581** | **0.008** | **0.001** | **1.50E-14** |
| **rs11743810** | **5** | **1.38E+08** | **T** | **C** | **0.56** | **0.008** | **0.001** | **3.60E-11** |
| **rs112965849** | **5** | **1.58E+08** | **A** | **AT** | **0.787** | **0.009** | **0.001** | **3.20E-12** |
| **rs2431752** | **5** | **1.63E+08** | **A** | **G** | **0.106** | **0.011** | **0.002** | **7.50E-10** |
| **rs116483731** | **5** | **1.69E+08** | **A** | **G** | **0.008** | **0.051** | **0.006** | **1.10E-14** |
| **rs55646464** | **5** | **1.73E+08** | **G** | **T** | **0.7** | **0.009** | **0.001** | **1.30E-11** |
| **rs9379084** | **6** | **7231843** | **G** | **A** | **0.885** | **0.016** | **0.002** | **1.80E-20** |
| **rs62394490** | **6** | **25934018** | **T** | **A** | **0.405** | **0.009** | **0.001** | **4.80E-18** |
| **6:27223966_CTCTA_C** | **6** | **27223966** | **C** | **CTCTA** | **0.09** | **0.013** | **0.002** | **5.50E-10** |
| **6:28419903_GGT_G** | **6** | **28419903** | **G** | **GGT** | **0.464** | **0.007** | **0.001** | **6.50E-10** |
| **rs9266184** | **6** | **31324664** | **T** | **G** | **0.682** | **0.011** | **0.001** | **5.60E-21** |
| **rs199910997** | **6** | **32485655** | **A** | **G** | **0.44** | **0.011** | **0.001** | **1.70E-15** |
| **rs111643014** | **6** | **34165308** | **A** | **G** | **0.028** | **0.028** | **0.004** | **5.20E-15** |
| **rs11751920** | **6** | **34655818** | **C** | **G** | **0.022** | **0.028** | **0.004** | **3.10E-12** |
| **rs4714001** | **6** | **36638175** | **G** | **A** | **0.36** | **0.006** | **0.001** | **2.50E-08** |
| **rs6939861** | **6** | **41703041** | **G** | **A** | **0.738** | **0.011** | **0.001** | **2.50E-16** |
| **rs4715316** | **6** | **52628998** | **T** | **C** | **0.644** | **0.009** | **0.001** | **4.40E-16** |
| **rs17185536** | **6** | **1.01E+08** | **T** | **C** | **0.244** | **0.007** | **0.001** | **3.20E-09** |
| **rs1890426** | **6** | **1.16E+08** | **C** | **T** | **0.401** | **0.007** | **0.001** | **1.80E-09** |
| **rs58321169** | **6** | **1.27E+08** | **C** | **T** | **0.734** | **0.009** | **0.001** | **1.30E-10** |
| **rs6900473** | **6** | **1.3E+08** | **A** | **G** | **0.312** | **0.01** | **0.001** | **4.80E-16** |
| **rs501470** | **6** | **1.61E+08** | **G** | **T** | **0.475** | **0.017** | **0.001** | **3.40E-55** |
| **rs62442919** | **7** | **1978384** | **A** | **G** | **0.382** | **0.008** | **0.001** | **5.40E-13** |
| **rs2106727** | **7** | **17287998** | **G** | **A** | **0.637** | **0.006** | **0.001** | **2.80E-08** |
| **rs7780562** | **7** | **26022414** | **C** | **A** | **0.794** | **0.008** | **0.001** | **3.00E-09** |
| **rs860262** | **7** | **28194397** | **A** | **C** | **0.502** | **0.011** | **0.001** | **3.10E-23** |
| **rs7808613** | **7** | **41746922** | **C** | **G** | **0.755** | **0.008** | **0.001** | **1.40E-09** |
| **rs1799831** | **7** | **44199142** | **C** | **T** | **0.843** | **0.01** | **0.002** | **4.50E-09** |
| **rs73109480** | **7** | **44811221** | **T** | **C** | **0.071** | **0.015** | **0.002** | **1.60E-12** |
| **rs6965401** | **7** | **46269012** | **G** | **A** | **0.936** | **0.015** | **0.002** | **9.00E-10** |
| **rs12536766** | **7** | **70158864** | **T** | **G** | **0.57** | **0.007** | **0.001** | **5.90E-09** |
| **rs17145750** | **7** | **73026378** | **T** | **C** | **0.161** | **0.013** | **0.002** | **1.40E-16** |
| **7:74298343_TGAGA_T** | **7** | **74298343** | **T** | **TGAGA** | **0.243** | **0.009** | **0.001** | **3.70E-13** |
| **rs1229492** | **7** | **81564122** | **T** | **C** | **0.268** | **0.011** | **0.001** | **1.20E-18** |
| **rs768159759** | **7** | **86880738** | **A** | **AG** | **0.075** | **0.014** | **0.002** | **3.60E-12** |
| **rs445** | **7** | **92408370** | **C** | **T** | **0.905** | **0.013** | **0.002** | **7.90E-12** |
| **rs6950023** | **7** | **97915635** | **G** | **T** | **0.814** | **0.03** | **0.001** | **2.10E-101** |
| **rs1859690** | **7** | **99227172** | **G** | **A** | **0.067** | **0.013** | **0.002** | **9.50E-10** |
| **rs12705095** | **7** | **1E+08** | **G** | **T** | **0.182** | **0.018** | **0.001** | **2.80E-35** |
| **rs35225944** | **7** | **1.02E+08** | **T** | **TA** | **0.67** | **0.009** | **0.001** | **4.40E-17** |
| **rs187437** | **7** | **1.16E+08** | **G** | **A** | **0.547** | **0.009** | **0.001** | **3.20E-17** |
| **rs157935** | **7** | **1.31E+08** | **G** | **T** | **0.304** | **0.012** | **0.001** | **5.00E-27** |
| **rs3812275** | **7** | **1.35E+08** | **C** | **A** | **0.403** | **0.007** | **0.001** | **9.60E-09** |
| **rs34372369** | **7** | **1.43E+08** | **A** | **G** | **0.052** | **0.021** | **0.003** | **1.80E-14** |
| **rs4725944** | **7** | **1.5E+08** | **C** | **G** | **0.39** | **0.009** | **0.001** | **2.30E-15** |
| **rs114949263** | **7** | **1.5E+08** | **C** | **T** | **0.111** | **0.017** | **0.002** | **9.10E-23** |
| **rs4841133** | **8** | **9183664** | **G** | **A** | **0.909** | **0.023** | **0.002** | **5.60E-36** |
| **rs13280055** | **8** | **11522353** | **G** | **A** | **0.866** | **0.008** | **0.002** | **3.80E-08** |
| **rs876435** | **8** | **22873533** | **G** | **A** | **0.41** | **0.007** | **0.001** | **8.90E-12** |
| **rs12543287** | **8** | **42334511** | **C** | **G** | **0.371** | **0.01** | **0.001** | **2.90E-21** |
| **rs10107182** | **8** | **59392737** | **T** | **C** | **0.663** | **0.013** | **0.001** | **1.10E-27** |
| **rs75349541** | **8** | **71152803** | **C** | **T** | **0.867** | **0.009** | **0.002** | **2.00E-08** |
| **rs11994858** | **8** | **81273210** | **G** | **A** | **0.654** | **0.011** | **0.001** | **4.40E-23** |
| **8:81430449_AT_A** | **8** | **81430449** | **A** | **AT** | **0.098** | **0.022** | **0.002** | **2.30E-35** |
| **8:81457499_CA_C** | **8** | **81457499** | **C** | **CA** | **0.204** | **0.014** | **0.001** | **2.10E-23** |
| **rs2721195** | **8** | **1.46E+08** | **T** | **C** | **0.475** | **0.011** | **0.001** | **7.50E-24** |
| **rs1567353** | **9** | **1033773** | **C** | **G** | **0.693** | **0.007** | **0.001** | **4.60E-09** |
| **rs10116426** | **9** | **4145648** | **C** | **A** | **0.432** | **0.008** | **0.001** | **9.20E-13** |
| **rs820503** | **9** | **6667928** | **C** | **A** | **0.863** | **0.011** | **0.002** | **2.90E-11** |
| **9:19084633_CT_C** | **9** | **19084633** | **C** | **CT** | **0.24** | **0.007** | **0.001** | **3.80E-08** |
| **rs35234337** | **9** | **35661243** | **C** | **T** | **0.743** | **0.007** | **0.001** | **4.50E-08** |
| **rs10868080** | **9** | **86626769** | **T** | **A** | **0.256** | **0.021** | **0.001** | **9.50E-63** |
| **rs56237852** | **9** | **1E+08** | **C** | **A** | **0.826** | **0.008** | **0.002** | **1.00E-08** |
| **9:112204335_AT_A** | **9** | **1.12E+08** | **AT** | **A** | **0.864** | **0.01** | **0.002** | **2.60E-08** |
| **rs62580766** | **9** | **1.13E+08** | **T** | **C** | **0.182** | **0.01** | **0.002** | **1.60E-11** |
| **rs4979372** | **9** | **1.17E+08** | **C** | **T** | **0.487** | **0.012** | **0.001** | **2.60E-28** |
| **rs1570516** | **9** | **1.19E+08** | **T** | **C** | **0.766** | **0.007** | **0.001** | **2.10E-08** |
| **9:123780856_AAAGGAAGG_A** | **9** | **1.24E+08** | **AAAGGAAGG** | **A** | **0.26** | **0.007** | **0.001** | **9.20E-09** |
| **9:125949547_TA_T** | **9** | **1.26E+08** | **TA** | **T** | **0.052** | **0.017** | **0.003** | **2.00E-08** |
| **rs9697210** | **9** | **1.31E+08** | **G** | **A** | **0.855** | **0.018** | **0.002** | **5.00E-31** |
| **rs34771269** | **9** | **1.37E+08** | **CT** | **C** | **0.295** | **0.012** | **0.001** | **1.60E-21** |
| **rs3780190** | **9** | **1.39E+08** | **G** | **A** | **0.545** | **0.012** | **0.001** | **1.50E-25** |
| **rs79717793** | **10** | **5262267** | **G** | **A** | **0.845** | **0.023** | **0.002** | **4.80E-58** |
| **rs3781085** | **10** | **13370958** | **T** | **G** | **0.585** | **0.006** | **0.001** | **3.60E-09** |
| **rs3006593** | **10** | **31171626** | **C** | **G** | **0.38** | **0.007** | **0.001** | **2.70E-09** |
| **rs34390319** | **10** | **63960611** | **C** | **T** | **0.1** | **0.015** | **0.002** | **2.20E-13** |
| **rs10822153** | **10** | **65056813** | **A** | **C** | **0.472** | **0.063** | **0.001** | **3.9E-714** |
| **rs6480299** | **10** | **69631393** | **C** | **G** | **0.33** | **0.011** | **0.001** | **5.00E-18** |
| **rs1782652** | **10** | **81074125** | **T** | **A** | **0.619** | **0.012** | **0.001** | **2.70E-28** |
| **rs2259305** | **10** | **93615903** | **G** | **A** | **0.523** | **0.012** | **0.001** | **8.30E-28** |
| **rs856534** | **10** | **94810665** | **A** | **G** | **0.387** | **0.011** | **0.001** | **1.30E-22** |
| **rs3927496** | **10** | **1.02E+08** | **A** | **T** | **0.572** | **0.01** | **0.001** | **1.40E-19** |
| **rs997907** | **10** | **1.06E+08** | **C** | **A** | **0.504** | **0.008** | **0.001** | **8.00E-11** |
| **rs7096937** | **10** | **1.14E+08** | **T** | **C** | **0.269** | **0.009** | **0.001** | **1.40E-12** |
| **rs17747324** | **10** | **1.15E+08** | **T** | **C** | **0.772** | **0.008** | **0.001** | **5.60E-10** |
| **rs775181992** | **11** | **2179864** | **A** | **AGCCCT** | **0.239** | **0.01** | **0.001** | **3.60E-16** |
| **rs11601507** | **11** | **5701074** | **A** | **C** | **0.069** | **0.015** | **0.002** | **6.50E-12** |
| **rs1037169** | **11** | **13361005** | **T** | **C** | **0.313** | **0.01** | **0.001** | **4.00E-16** |
| **rs778571122** | **11** | **16247860** | **GTGTTTTTTTTGTTTTTGTTTT** | **G** | **0.613** | **0.01** | **0.001** | **7.40E-18** |
| **rs62618693** | **11** | **32956492** | **T** | **C** | **0.046** | **0.017** | **0.003** | **7.90E-11** |
| **rs12364060** | **11** | **45889775** | **T** | **C** | **0.237** | **0.008** | **0.001** | **1.80E-08** |
| **rs143709973** | **11** | **59572669** | **A** | **C** | **0.955** | **0.013** | **0.003** | **3.60E-08** |
| **rs174528** | **11** | **61543499** | **T** | **C** | **0.62** | **0.008** | **0.001** | **4.00E-11** |
| **rs2277283** | **11** | **61908440** | **C** | **T** | **0.317** | **0.008** | **0.001** | **1.20E-09** |
| **11:65227616_CAAA_C** | **11** | **65227616** | **CAAA** | **C** | **0.667** | **0.008** | **0.001** | **6.50E-11** |
| **rs4645936** | **11** | **65485478** | **G** | **A** | **0.947** | **0.018** | **0.003** | **4.10E-12** |
| **rs12797706** | **11** | **65561369** | **A** | **G** | **0.235** | **0.013** | **0.001** | **1.40E-22** |
| **rs4988308** | **11** | **68106842** | **G** | **A** | **0.557** | **0.006** | **0.001** | **4.70E-08** |
| **rs631695** | **11** | **69283303** | **T** | **G** | **0.418** | **0.018** | **0.001** | **2.00E-61** |
| **rs11398692** | **11** | **72501407** | **C** | **CT** | **0.52** | **0.007** | **0.001** | **3.60E-08** |
| **rs10895277** | **11** | **1.02E+08** | **A** | **G** | **0.659** | **0.01** | **0.001** | **1.30E-17** |
| **rs665731** | **11** | **1.14E+08** | **T** | **C** | **0.19** | **0.008** | **0.001** | **2.60E-09** |
| **rs757253787** | **11** | **1.19E+08** | **T** | **TC** | **0.271** | **0.007** | **0.001** | **3.40E-09** |
| **rs2156805** | **11** | **1.23E+08** | **G** | **A** | **0.508** | **0.006** | **0.001** | **4.40E-08** |
| **rs56196860** | **12** | **2908330** | **A** | **C** | **0.032** | **0.025** | **0.003** | **2.30E-15** |
| **rs76895963** | **12** | **4384844** | **G** | **T** | **0.021** | **0.066** | **0.004** | **1.90E-55** |
| **rs3782735** | **12** | **6885076** | **A** | **G** | **0.6** | **0.007** | **0.001** | **2.50E-09** |
| **rs4762755** | **12** | **20595535** | **T** | **C** | **0.57** | **0.007** | **0.001** | **5.10E-11** |
| **rs1871395** | **12** | **21352315** | **A** | **G** | **0.848** | **0.032** | **0.002** | **2.70E-100** |
| **rs10083137** | **12** | **24198178** | **A** | **G** | **0.965** | **0.02** | **0.003** | **9.20E-14** |
| **rs75130744** | **12** | **25410741** | **G** | **C** | **0.928** | **0.029** | **0.002** | **3.00E-44** |
| **rs864899** | **12** | **51221127** | **A** | **G** | **0.416** | **0.009** | **0.001** | **1.90E-17** |
| **rs12818938** | **12** | **53783182** | **T** | **G** | **0.832** | **0.008** | **0.002** | **1.10E-08** |
| **rs540730** | **12** | **57807114** | **T** | **C** | **0.246** | **0.018** | **0.001** | **1.90E-48** |
| **rs1351394** | **12** | **66351826** | **T** | **C** | **0.491** | **0.008** | **0.001** | **4.10E-11** |
| **rs61929307** | **12** | **69997422** | **G** | **T** | **0.341** | **0.007** | **0.001** | **2.00E-08** |
| **12:93632423_CA_C** | **12** | **93632423** | **CA** | **C** | **0.863** | **0.012** | **0.002** | **3.30E-14** |
| **rs191591035** | **12** | **99868285** | **G** | **C** | **0.997** | **0.092** | **0.013** | **1.00E-12** |
| **rs61755050** | **12** | **1.01E+08** | **T** | **C** | **0.994** | **0.125** | **0.007** | **3.80E-72** |
| **rs11111274** | **12** | **1.03E+08** | **G** | **A** | **0.263** | **0.008** | **0.001** | **1.30E-10** |
| **rs7314285** | **12** | **1.12E+08** | **G** | **T** | **0.069** | **0.031** | **0.002** | **4.20E-50** |
| **rs565728741** | **12** | **1.21E+08** | **C** | **CA** | **0.899** | **0.013** | **0.002** | **3.70E-10** |
| **rs9738226** | **12** | **1.21E+08** | **G** | **A** | **0.623** | **0.019** | **0.001** | **7.80E-61** |
| **rs41284816** | **13** | **50655989** | **T** | **G** | **0.019** | **0.022** | **0.004** | **5.50E-09** |
| **rs769447487** | **13** | **95247137** | **T** | **TTA** | **0.307** | **0.011** | **0.001** | **1.50E-19** |
| **rs116338429** | **13** | **1.15E+08** | **T** | **C** | **0.171** | **0.01** | **0.002** | **2.40E-11** |
| **13:115013423_TTCTC_T** | **13** | **1.15E+08** | **TTCTC** | **T** | **0.773** | **0.01** | **0.001** | **2.00E-12** |
| **rs75170914** | **14** | **23698566** | **T** | **C** | **0.255** | **0.013** | **0.001** | **5.10E-26** |
| **rs11621792** | **14** | **24871926** | **C** | **T** | **0.546** | **0.013** | **0.001** | **2.00E-28** |
| **14:25948829_AAC_A** | **14** | **25948829** | **AAC** | **A** | **0.235** | **0.008** | **0.001** | **2.70E-09** |
| **rs72683923** | **14** | **50735947** | **C** | **T** | **0.02** | **0.029** | **0.004** | **6.30E-15** |
| **rs2239222** | **14** | **73011885** | **G** | **A** | **0.35** | **0.012** | **0.001** | **8.80E-20** |
| **rs13379043** | **14** | **74250126** | **C** | **T** | **0.28** | **0.008** | **0.001** | **2.90E-11** |
| **rs1005421** | **14** | **89886940** | **C** | **T** | **0.585** | **0.006** | **0.001** | **7.20E-09** |
| **rs28929474** | **14** | **94844947** | **T** | **C** | **0.02** | **0.133** | **0.004** | **1.20E-252** |
| **rs773816354** | **14** | **1.01E+08** | **TAACAGGAGTTAAAAGTGTCG** | **T** | **0.511** | **0.011** | **0.001** | **8.30E-25** |
| **rs3742366** | **14** | **1.04E+08** | **C** | **T** | **0.346** | **0.009** | **0.001** | **3.60E-17** |
| **rs45490496** | **14** | **1.05E+08** | **A** | **T** | **0.387** | **0.009** | **0.001** | **2.40E-14** |
| **rs28562483** | **15** | **31660799** | **G** | **T** | **0.872** | **0.01** | **0.002** | **3.40E-08** |
| **rs8038032** | **15** | **35302869** | **G** | **A** | **0.322** | **0.01** | **0.001** | **6.60E-16** |
| **rs7175361** | **15** | **41048058** | **A** | **G** | **0.158** | **0.009** | **0.002** | **3.40E-09** |
| **rs139974673** | **15** | **44027885** | **T** | **C** | **0.975** | **0.078** | **0.004** | **4.60E-110** |
| **rs549677458** | **15** | **45038700** | **A** | **G** | **0.998** | **0.116** | **0.016** | **2.60E-13** |
| **rs191884522** | **15** | **52694917** | **C** | **T** | **0.998** | **0.117** | **0.014** | **1.60E-14** |
| **rs149663666** | **15** | **53062068** | **T** | **C** | **0.991** | **0.048** | **0.006** | **1.10E-15** |
| **rs149624078** | **15** | **53728710** | **C** | **T** | **0.986** | **0.096** | **0.005** | **1.00E-91** |
| **rs8030357** | **15** | **53746169** | **G** | **T** | **0.285** | **0.01** | **0.001** | **9.30E-17** |
| **rs528350911** | **15** | **53747228** | **C** | **G** | **0.994** | **0.043** | **0.008** | **4.60E-10** |
| **rs62012358** | **15** | **59923927** | **G** | **A** | **0.335** | **0.008** | **0.001** | **1.70E-13** |
| **rs56187480** | **15** | **63789479** | **G** | **A** | **0.655** | **0.014** | **0.001** | **1.20E-33** |
| **rs370654105** | **15** | **66821676** | **C** | **CT** | **0.754** | **0.009** | **0.001** | **1.10E-11** |
| **rs8038465** | **15** | **73978337** | **T** | **C** | **0.425** | **0.007** | **0.001** | **4.60E-10** |
| **rs72753908** | **15** | **83334856** | **C** | **T** | **0.925** | **0.013** | **0.002** | **1.90E-09** |
| **rs11856926** | **15** | **96223649** | **G** | **A** | **0.551** | **0.011** | **0.001** | **1.10E-24** |
| **rs56332871** | **15** | **96714816** | **A** | **C** | **0.272** | **0.031** | **0.001** | **6.00E-140** |
| **16:1786202_TGTGTGACCATCC_T** | **16** | **1786202** | **T** | **TGTGTGACCATCC** | **0.116** | **0.011** | **0.002** | **1.50E-10** |
| **rs36108764** | **16** | **4624130** | **G** | **A** | **0.805** | **0.009** | **0.001** | **1.70E-11** |
| **rs55962409** | **16** | **11799367** | **C** | **A** | **0.707** | **0.007** | **0.001** | **1.80E-09** |
| **rs12928099** | **16** | **15150505** | **A** | **C** | **0.295** | **0.009** | **0.001** | **7.20E-13** |
| **rs11419346** | **16** | **24837838** | **C** | **CT** | **0.797** | **0.008** | **0.001** | **3.50E-08** |
| **rs2288004** | **16** | **31054040** | **G** | **C** | **0.619** | **0.009** | **0.001** | **3.40E-16** |
| **rs148082013** | **16** | **34599725** | **G** | **C** | **0.995** | **0.056** | **0.01** | **1.30E-09** |
| **rs185073832** | **16** | **47314028** | **C** | **A** | **0.998** | **0.111** | **0.016** | **4.20E-12** |
| **rs246192** | **16** | **58544295** | **G** | **C** | **0.48** | **0.008** | **0.001** | **9.80E-14** |
| **rs34006916** | **16** | **68428844** | **G** | **A** | **0.945** | **0.018** | **0.003** | **7.20E-12** |
| **rs562609617** | **16** | **79745672** | **GT** | **G** | **0.314** | **0.011** | **0.001** | **4.50E-17** |
| **rs28650012** | **16** | **80497341** | **G** | **C** | **0.271** | **0.008** | **0.001** | **4.50E-11** |
| **rs4782568** | **16** | **83980529** | **G** | **C** | **0.452** | **0.017** | **0.001** | **7.00E-49** |
| **rs11641834** | **16** | **88070573** | **C** | **T** | **0.568** | **0.01** | **0.001** | **9.20E-22** |
| **rs904801** | **16** | **88517105** | **A** | **G** | **0.27** | **0.011** | **0.001** | **1.50E-18** |
| **rs2287322** | **17** | **1641035** | **G** | **A** | **0.223** | **0.018** | **0.001** | **3.00E-42** |
| **rs184640919** | **17** | **6274048** | **G** | **A** | **0.999** | **0.106** | **0.018** | **8.80E-09** |
| **rs1799941** | **17** | **7533423** | **A** | **G** | **0.261** | **0.12** | **0.001** | **9.2E-2048** |
| **rs6258** | **17** | **7534678** | **C** | **T** | **0.993** | **0.608** | **0.007** | **1.2E-1823** |
| **17:8567107_CA_C** | **17** | **8567107** | **CA** | **C** | **0.994** | **0.04** | **0.007** | **1.90E-09** |
| **rs8066941** | **17** | **9588450** | **T** | **G** | **0.761** | **0.016** | **0.001** | **7.70E-33** |
| **rs17669311** | **17** | **13837051** | **G** | **A** | **0.611** | **0.01** | **0.001** | **4.40E-18** |
| **17:16062400_TA_T** | **17** | **16062400** | **T** | **TA** | **0.556** | **0.007** | **0.001** | **5.10E-12** |
| **rs12950562** | **17** | **17995166** | **T** | **C** | **0.614** | **0.012** | **0.001** | **1.60E-28** |
| **rs2002094** | **17** | **28044991** | **C** | **A** | **0.908** | **0.014** | **0.002** | **4.00E-13** |
| **rs2905801** | **17** | **29524974** | **T** | **C** | **0.705** | **0.013** | **0.001** | **1.20E-28** |
| **rs11867902** | **17** | **38592070** | **G** | **A** | **0.974** | **0.022** | **0.003** | **2.30E-10** |
| **rs591939** | **17** | **40698075** | **A** | **G** | **0.751** | **0.007** | **0.001** | **8.70E-09** |
| **rs111767734** | **17** | **42111443** | **T** | **G** | **0.988** | **0.032** | **0.005** | **9.10E-10** |
| **rs142627977** | **17** | **45562744** | **C** | **CAAGT** | **0.509** | **0.012** | **0.001** | **1.10E-26** |
| **rs11655704** | **17** | **47448172** | **C** | **T** | **0.315** | **0.03** | **0.001** | **1.50E-147** |
| **rs753645751** | **17** | **59240686** | **G** | **GA** | **0.797** | **0.008** | **0.001** | **2.90E-09** |
| **rs1991401** | **17** | **62502435** | **G** | **A** | **0.313** | **0.007** | **0.001** | **9.90E-09** |
| **rs1801689** | **17** | **64210580** | **A** | **C** | **0.97** | **0.034** | **0.003** | **6.20E-25** |
| **rs9914426** | **17** | **65126641** | **G** | **C** | **0.539** | **0.009** | **0.001** | **1.20E-14** |
| **17:67080811_CT_C** | **17** | **67080811** | **C** | **CT** | **0.646** | **0.01** | **0.001** | **2.40E-13** |
| **rs202128511** | **17** | **73226458** | **T** | **TTG** | **0.214** | **0.012** | **0.001** | **1.10E-17** |
| **rs7210574** | **17** | **73824121** | **C** | **T** | **0.329** | **0.01** | **0.001** | **2.30E-20** |
| **rs754959778** | **17** | **77784494** | **CGT** | **C** | **0.661** | **0.006** | **0.001** | **2.30E-08** |
| **rs36013981** | **17** | **79493307** | **A** | **G** | **0.604** | **0.006** | **0.001** | **8.20E-11** |
| **rs111917063** | **18** | **2656893** | **C** | **G** | **0.979** | **0.02** | **0.004** | **2.30E-08** |
| **rs55855238** | **18** | **55089715** | **C** | **T** | **0.649** | **0.008** | **0.001** | **3.00E-14** |
| **rs10871794** | **18** | **59342210** | **A** | **G** | **0.697** | **0.007** | **0.001** | **6.00E-09** |
| **rs12454712** | **18** | **60845884** | **C** | **T** | **0.377** | **0.008** | **0.001** | **1.20E-13** |
| **rs1788641** | **18** | **71949629** | **A** | **G** | **0.688** | **0.008** | **0.001** | **8.00E-12** |
| **rs62134282** | **19** | **677646** | **T** | **C** | **0.5** | **0.007** | **0.001** | **5.80E-10** |
| **rs1640267** | **19** | **2789337** | **C** | **T** | **0.286** | **0.017** | **0.001** | **1.90E-45** |
| **rs11539938** | **19** | **3062857** | **C** | **T** | **0.421** | **0.012** | **0.001** | **3.40E-19** |
| **rs60018147** | **19** | **3375572** | **G** | **A** | **0.121** | **0.013** | **0.002** | **1.50E-12** |
| **19:7223973_TTTG_T** | **19** | **7223973** | **TTTG** | **T** | **0.578** | **0.012** | **0.001** | **1.00E-26** |
| **rs10411958** | **19** | **13113641** | **C** | **T** | **0.533** | **0.007** | **0.001** | **2.20E-09** |
| **rs10421262** | **19** | **14172951** | **T** | **G** | **0.576** | **0.011** | **0.001** | **9.30E-24** |
| **rs202200760** | **19** | **17346854** | **C** | **G** | **0.039** | **0.071** | **0.003** | **5.00E-112** |
| **rs35824797** | **19** | **19456264** | **C** | **T** | **0.922** | **0.012** | **0.002** | **1.00E-11** |
| **rs67527887** | **19** | **33913745** | **T** | **C** | **0.513** | **0.009** | **0.001** | **2.00E-14** |
| **rs45512696** | **19** | **35550878** | **T** | **C** | **0.174** | **0.021** | **0.002** | **9.80E-47** |
| **rs11666245** | **19** | **38229926** | **G** | **A** | **0.953** | **0.017** | **0.003** | **1.00E-11** |
| **rs7254776** | **19** | **45227742** | **C** | **T** | **0.352** | **0.006** | **0.001** | **3.60E-08** |
| **rs190712692** | **19** | **45425178** | **A** | **G** | **0.054** | **0.017** | **0.003** | **8.60E-12** |
| **rs34255979** | **19** | **46384830** | **T** | **C** | **0.119** | **0.028** | **0.002** | **2.70E-63** |
| **rs111981233** | **19** | **50016479** | **G** | **T** | **0.079** | **0.025** | **0.002** | **7.70E-36** |
| **rs4077285** | **19** | **56599405** | **G** | **C** | **0.906** | **0.012** | **0.002** | **1.70E-08** |
| **rs144794875** | **19** | **58391476** | **G** | **A** | **0.145** | **0.009** | **0.002** | **2.80E-09** |
| **rs771193934** | **20** | **17844518** | **AGGCATGCTGCCAAGAAT** | **A** | **0.342** | **0.007** | **0.001** | **1.80E-09** |
| **rs186915841** | **20** | **22433293** | **C** | **T** | **0.998** | **0.098** | **0.015** | **7.40E-11** |
| **rs13042148** | **20** | **32298286** | **C** | **T** | **0.844** | **0.013** | **0.002** | **5.60E-20** |
| **rs749761903** | **20** | **32971168** | **A** | **AT** | **0.447** | **0.007** | **0.001** | **4.60E-11** |
| **rs4812336** | **20** | **37547374** | **A** | **G** | **0.303** | **0.008** | **0.001** | **5.70E-12** |
| **rs3795128** | **20** | **39774163** | **C** | **T** | **0.484** | **0.01** | **0.001** | **1.70E-23** |
| **rs3746575** | **20** | **43058096** | **G** | **C** | **0.597** | **0.017** | **0.001** | **4.70E-49** |
| **rs4809604** | **20** | **45550489** | **T** | **G** | **0.574** | **0.007** | **0.001** | **1.70E-09** |
| **rs201764793** | **20** | **47531640** | **TTTC** | **T** | **0.342** | **0.007** | **0.001** | **4.20E-09** |
| **rs78319058** | **20** | **49020571** | **T** | **C** | **0.022** | **0.025** | **0.004** | **2.30E-11** |
| **rs73909848** | **20** | **49570403** | **G** | **A** | **0.073** | **0.016** | **0.002** | **2.50E-14** |
| **rs1058319** | **20** | **62374389** | **T** | **C** | **0.135** | **0.01** | **0.002** | **9.00E-10** |
| **rs2234694** | **21** | **33038865** | **A** | **C** | **0.956** | **0.014** | **0.003** | **8.90E-09** |
| **rs2238799** | **22** | **20109325** | **G** | **A** | **0.39** | **0.005** | **0.001** | **9.90E-09** |
| **rs41412647** | **22** | **24292178** | **C** | **A** | **0.431** | **0.01** | **0.001** | **5.10E-20** |
| **rs6005840** | **22** | **29101357** | **A** | **G** | **0.327** | **0.016** | **0.001** | **1.50E-40** |
| **rs5749082** | **22** | **30770603** | **T** | **A** | **0.293** | **0.009** | **0.001** | **8.70E-15** |
| **rs7286550** | **22** | **38196760** | **G** | **A** | **0.701** | **0.007** | **0.001** | **2.50E-09** |
| **rs738409** | **22** | **44324727** | **G** | **C** | **0.216** | **0.029** | **0.001** | **3.40E-98** |
| **rs112352679** | **22** | **50318514** | **T** | **C** | **0.81** | **0.009** | **0.001** | **2.10E-11** |
| **rs211644** | **23** | **2862721** | **C** | **T** | **0.691** | **0.006** | **0.001** | **6.90E-11** |
| **rs73631501** | **23** | **9572187** | **T** | **C** | **0.097** | **0.011** | **0.001** | **6.50E-17** |
| **rs780309846** | **23** | **15286456** | **AAAAAAC** | **A** | **0.472** | **0.005** | **0.001** | **8.10E-11** |
| **rs6632893** | **23** | **16789928** | **A** | **C** | **0.706** | **0.006** | **0.001** | **6.80E-11** |
| **rs7892835** | **23** | **20184037** | **G** | **A** | **0.854** | **0.008** | **0.001** | **6.00E-17** |
| **rs148427769** | **23** | **54004538** | **G** | **C** | **0.029** | **0.014** | **0.002** | **2.00E-09** |
| **rs778239811** | **23** | **55529635** | **T** | **TAC** | **0.732** | **0.006** | **0.001** | **6.80E-11** |
| **rs200138078** | **23** | **66173624** | **C** | **T** | **0.852** | **0.009** | **0.001** | **5.30E-17** |
| **rs1418334** | **23** | **1.1E+08** | **G** | **A** | **0.39** | **0.042** | **0.001** | **1.6E-604** |
| **rs73266316** | **23** | **1.11E+08** | **T** | **C** | **0.072** | **0.013** | **0.002** | **1.90E-14** |
| **rs12690320** | **23** | **1.15E+08** | **G** | **A** | **0.557** | **0.005** | **0.001** | **3.40E-10** |
| **rs73547906** | **23** | **1.23E+08** | **C** | **T** | **0.712** | **0.006** | **0.001** | **8.40E-11** |
| **rs180956060** | **23** | **1.29E+08** | **G** | **A** | **0.897** | **0.014** | **0.001** | **3.00E-23** |
| **X:133611871_AT_A** | **23** | **1.34E+08** | **A** | **AT** | **0.098** | **0.009** | **0.001** | **2.00E-11** |
| **rs2283760** | **23** | **1.54E+08** | **C** | **T** | **0.189** | **0.005** | **0.001** | **5.00E-09** |

**Table S7.** Definitions for heart failure used within this study, based on the World Health Organization International Statistical Classification of Diseases and Related Health Problems 10th Revision (ICD-10) codes

| ***ICD-10 code*** | ***Sub-code*** | ***Explanation*** |
| --- | --- | --- |
| I11.0 |  | Hypertensive heart disease with heart failure |
| I13.0 |  | Hypertensive heart and chronic kidney disease with heart failure and stage 1 through stage 4 chronic kidney disease, or unspecified chronic kidney disease |
| I13.2 |  | Hypertensive heart and chronic kidney disease with heart failure and with stage 5 chronic kidney disease, or end stage renal disease |
| I50.X | 150 | Heart failure |
|  | I50.1 | Left ventricular failure, unspecified |
|  | I50.2 | Systolic (congestive) heart failure |
|  | I50.3 | Diastolic (congestive) heart failure |
|  | I50.4 | Combined systolic (congestive) and diastolic (congestive) heart failure |
|  | I50.8 | Other heart failure |
|  | I50.9 | Heart failure, unspecified |
| ***ICD-9 code*** | ***Sub-code*** | ***Explanation*** |
| 402.01 |  | Malignant hypertensive heart disease with heart failure |
| 402.11, 402.91 |  | Hypertensive heart failure |
| 404.01, 404.11, 404.91 |  | Hypertensive heart and chronic kidney disease, benign, with heart failure |
| 404.03, 404.13, 404.93 |  | Hypertensive heart and chronic kidney disease with heart failure and with stage 5 chronic kidney disease, or end stage renal disease |
| 428.X | 428 | Heart failure |
|  | 428 | Congestive heart failure, unspecified |
|  | 428.1 | Left heart failure |
|  | 428.2 | Systolic heart failure |
|  | 428.3 | Diastolic heart failure |
|  | 428.4 | Combined systolic and diastolic heart failure |
|  | 428.9 | Heart failure, unspecified |
| ***UK Biobank*** |  | ***Explanation*** |
| 1076 |  | Self-reported medical conditions: heart failure/pulmonary odema |

**Table S8 –** F-statistics for instrument strength in all analyses..

| **Exposure** | **F-statistic** | **R-squared (%)** |
| --- | --- | --- |
| **Male bioavailable testosterone** | 72.77 | 6.5 |
| **Female bioavailable testosterone** | 140.01 | 8.92 |
| **Male total testosterone** | 136.81 | 13.99 |
| **Female total testosterone** | 91.75 | 9.19 |
| **Male sex hormone binding globulin** | 253.91 | 32.52 |
| **Female sex hormone binding globulin** | 296.23 | 36.96 |

**Table S9 –** Genome-wide significant (p<5.10^-8^) associations of all instrumental variants used for bioavailable testosterone in females.

| **SNP** | **Allele1** | **Allele2** | **Associated trait** | **Study** |
| --- | --- | --- | --- | --- |
| rs1989147 | T | C | Getting up in morning | Neale B |
| rs1989147 | T | C | Morning or evening person | Neale B |
| rs114165349 | C | G | Plateletcrit | Astle W |
| rs114165349 | C | G | Height | Neale B |
| rs114165349 | C | G | Self-reported gout | Neale B |
| rs114165349 | C | G | Treatment with cholesterol lowering medication | Neale B |
| rs2391139 | G | T | Granulocyte percentage of myeloid white cells | Astle W |
| rs2391139 | G | T | Lymphocyte percentage of white cells | Astle W |
| rs2391139 | G | T | Monocyte count | Astle W |
| rs2391139 | G | T | Monocyte percentage of white cells | Astle W |
| rs2391139 | G | T | Height | Neale B |
| rs2391139 | G | T | Sitting height | Neale B |
| rs7418101 | A | G | Eosinophil count | Astle W |
| rs7418101 | A | G | Eosinophil percentage of granulocytes | Astle W |
| rs7418101 | A | G | Eosinophil percentage of white cells | Astle W |
| rs7418101 | A | G | Monocyte count | Astle W |
| rs7418101 | A | G | Neutrophil percentage of granulocytes | Astle W |
| rs7418101 | A | G | Sum eosinophil basophil counts | Astle W |
| rs7418101 | A | G | Qualifications: college or university degree | Neale B |
| rs140584594 | A | G | Platelet count | Astle W |
| rs2282248 | T | C | Mean platelet volume | Astle W |
| rs351370 | C | T | Platelet count | Astle W |
| rs351370 | C | T | Platelet distribution width | Astle W |
| rs351370 | C | T | Medication for cholesterol, blood pressure or diabetes: blood pressure medication | Neale B |
| rs351370 | C | T | Self-reported hypertension | Neale B |
| rs351370 | C | T | Treatment with amlodipine | Neale B |
| rs351370 | C | T | Vascular or heart problems diagnosed by doctor: high blood pressure | Neale B |
| rs351370 | C | T | Vascular or heart problems diagnosed by doctor: none of the above | Neale B |
| rs3832126 | - | T | Mean corpuscular hemoglobin | Astle W |
| rs3832126 | - | T | Monocyte percentage of white cells | Astle W |
| rs3832126 | - | T | Red cell distribution width | Astle W |
| rs113247979 | C | T | Cause of death: inquest adjourned death | Neale B |
| rs590097 | G | T | Mean corpuscular hemoglobin | Astle W |
| rs590097 | G | T | Dihydroepiandrosterone sulphate | Prins B |
| rs78058190 | A | G | HDL cholesterol | Surakka I |
| rs1515098 | C | T | Type II diabetes | DIAGRAM |
| rs1515098 | C | T | Type II diabetes adjusted for BMI | DIAGRAM |
| rs1515098 | C | T | Waist circumference in males | GIANT |
| rs1515098 | C | T | High density lipoprotein | GLGC |
| rs1515098 | C | T | High density lipoprotein | GLGC |
| rs1515098 | C | T | Triglycerides | GLGC |
| rs1515098 | C | T | HDL cholesterol | GLGC |
| rs1515098 | C | T | Body fat percentage | Lu Y |
| rs1515098 | C | T | log Fasting insulin adjusted for BMI | MAGIC |
| rs1515098 | C | T | Insulin sensitivity index adjusted for BMI interaction | Walford GA |
| rs1515098 | C | T | Insulin sensitivity index adjusted for BMI | Walford GA |
| rs1515098 | C | T | Arm fat percentage left | Neale B |
| rs1515098 | C | T | Arm fat percentage right | Neale B |
| rs1515098 | C | T | Arm fat-free mass left | Neale B |
| rs1515098 | C | T | Arm fat-free mass right | Neale B |
| rs1515098 | C | T | Arm predicted mass left | Neale B |
| rs1515098 | C | T | Arm predicted mass right | Neale B |
| rs1515098 | C | T | Body fat percentage | Neale B |
| rs1515098 | C | T | Diabetes diagnosed by doctor | Neale B |
| rs1515098 | C | T | Impedance of arm left | Neale B |
| rs1515098 | C | T | Impedance of arm right | Neale B |
| rs1515098 | C | T | Impedance of whole body | Neale B |
| rs1515098 | C | T | Medication for cholesterol, blood pressure or diabetes: blood pressure medication | Neale B |
| rs1515098 | C | T | Medication for cholesterol, blood pressure or diabetes: none of the above | Neale B |
| rs1515098 | C | T | Self-reported diabetes | Neale B |
| rs1515098 | C | T | Self-reported high cholesterol | Neale B |
| rs1515098 | C | T | Trunk fat mass | Neale B |
| rs1515098 | C | T | Trunk fat percentage | Neale B |
| rs1515098 | C | T | Trunk fat-free mass | Neale B |
| rs1515098 | C | T | Trunk predicted mass | Neale B |
| rs4135247 | A | G | Granulocyte percentage of myeloid white cells | Astle W |
| rs4135247 | A | G | Hematocrit | Astle W |
| rs4135247 | A | G | Hemoglobin concentration | Astle W |
| rs4135247 | A | G | Lymphocyte count | Astle W |
| rs4135247 | A | G | Monocyte count | Astle W |
| rs4135247 | A | G | Platelet count | Astle W |
| rs4135247 | A | G | Plateletcrit | Astle W |
| rs13072623 | A | G | Mean corpuscular volume | Astle W |
| rs6788984 | A | G | Heel bone mineral density | Neale B |
| rs13092573 | C | T | Lymphocyte count | Astle W |
| rs13092573 | C | T | Lymphocyte percentage of white cells | Astle W |
| rs13092573 | C | T | Monocyte count | Astle W |
| rs13092573 | C | T | Neutrophil percentage of white cells | Astle W |
| rs13092573 | C | T | White blood cell count | Astle W |
| rs62263023 | A | T | High light scatter percentage of red cells | Astle W |
| rs62263023 | A | T | High light scatter reticulocyte count | Astle W |
| rs62263023 | A | T | Immature fraction of reticulocytes | Astle W |
| rs62263023 | A | T | Reticulocyte count | Astle W |
| rs62263023 | A | T | Reticulocyte fraction of red cells | Astle W |
| rs563306865 | - | T | High light scatter percentage of red cells | Astle W |
| rs563306865 | - | T | High light scatter reticulocyte count | Astle W |
| rs563306865 | - | T | Immature fraction of reticulocytes | Astle W |
| rs563306865 | - | T | Reticulocyte count | Astle W |
| rs563306865 | - | T | Reticulocyte fraction of red cells | Astle W |
| rs71633359 | C | T | Platelet count | Astle W |
| rs71633359 | C | T | Platelet count | Astle W |
| rs11733695 | A | G | Alcohol intake frequency | Neale B |
| rs11733695 | A | G | Alcohol intake versus 10 years previously | Neale B |
| rs11733695 | A | G | Average weekly red wine intake | Neale B |
| rs11733695 | A | G | Self-reported gout | Neale B |
| rs40270 | C | A | Hemoglobin concentration | Astle W |
| rs40270 | C | A | Body fat percentage | Neale B |
| rs40270 | C | A | Height | Neale B |
| rs40270 | C | A | Impedance of arm left | Neale B |
| rs40270 | C | A | Impedance of arm right | Neale B |
| rs40270 | C | A | Impedance of leg left | Neale B |
| rs40270 | C | A | Impedance of leg right | Neale B |
| rs40270 | C | A | Impedance of whole body | Neale B |
| rs40270 | C | A | Trunk fat mass | Neale B |
| rs40270 | C | A | Trunk fat percentage | Neale B |
| rs34341 | A | T | Body mass index | Akiyama M |
| rs34341 | A | T | Body mass index adjusted for smoking | GIANT |
| rs34341 | A | T | Body mass index adjusted for smoking | GIANT |
| rs34341 | A | T | Body mass index | GIANT |
| rs34341 | A | T | Body mass index | GIANT |
| rs34341 | A | T | Waist circumference in females | GIANT |
| rs34341 | A | T | Waist circumference | GIANT |
| rs34341 | A | T | Waist circumference | GIANT |
| rs34341 | A | T | Low density lipoprotein | GLGC |
| rs34341 | A | T | Low density lipoprotein | GLGC |
| rs34341 | A | T | Total cholesterol | GLGC |
| rs34341 | A | T | Total cholesterol | GLGC |
| rs34341 | A | T | LDL cholesterol | GLGC |
| rs34341 | A | T | Total cholesterol | GLGC |
| rs34341 | A | T | Arm fat mass left | Neale B |
| rs34341 | A | T | Arm fat mass right | Neale B |
| rs34341 | A | T | Arm fat percentage left | Neale B |
| rs34341 | A | T | Arm fat percentage right | Neale B |
| rs34341 | A | T | Arm fat-free mass left | Neale B |
| rs34341 | A | T | Arm fat-free mass right | Neale B |
| rs34341 | A | T | Arm predicted mass left | Neale B |
| rs34341 | A | T | Arm predicted mass right | Neale B |
| rs34341 | A | T | Basal metabolic rate | Neale B |
| rs34341 | A | T | Body fat percentage | Neale B |
| rs34341 | A | T | Body mass index | Neale B |
| rs34341 | A | T | Hip circumference | Neale B |
| rs34341 | A | T | Impedance of arm left | Neale B |
| rs34341 | A | T | Impedance of arm right | Neale B |
| rs34341 | A | T | Impedance of leg left | Neale B |
| rs34341 | A | T | Impedance of leg right | Neale B |
| rs34341 | A | T | Impedance of whole body | Neale B |
| rs34341 | A | T | Leg fat mass left | Neale B |
| rs34341 | A | T | Leg fat mass right | Neale B |
| rs34341 | A | T | Leg fat percentage left | Neale B |
| rs34341 | A | T | Leg fat percentage right | Neale B |
| rs34341 | A | T | Leg fat-free mass left | Neale B |
| rs34341 | A | T | Leg fat-free mass right | Neale B |
| rs34341 | A | T | Leg predicted mass left | Neale B |
| rs34341 | A | T | Leg predicted mass right | Neale B |
| rs34341 | A | T | Self-reported high cholesterol | Neale B |
| rs34341 | A | T | Trunk fat mass | Neale B |
| rs34341 | A | T | Trunk fat percentage | Neale B |
| rs34341 | A | T | Trunk fat-free mass | Neale B |
| rs34341 | A | T | Trunk predicted mass | Neale B |
| rs34341 | A | T | Waist circumference | Neale B |
| rs34341 | A | T | Weight | Neale B |
| rs34341 | A | T | Whole body fat mass | Neale B |
| rs34341 | A | T | Whole body fat-free mass | Neale B |
| rs34341 | A | T | Whole body water mass | Neale B |
| rs34341 | A | T | Low density lipoprotein | Spracklen CN |
| rs34341 | A | T | Total cholesterol | Spracklen CN |
| rs73350117 | A | C | Red cell distribution width | Astle W |
| rs1432679 | C | T | Breast cancer | Michailidou K |
| rs1432679 | C | T | Breast cancer Estrogen receptor positive breast cancer | Michailidou K |
| rs1432679 | C | T | Breast cancer invasive breast cancer | Michailidou K |
| rs1432679 | C | T | Breast cancer | Michailidou K |
| rs1432679 | C | T | Breast cancer | Michailidou K |
| rs1432679 | C | T | Breast cancer | Michailidou K |
| rs1432679 | C | T | Breast cancer estrogen receptor negative | Milne RL |
| rs1432679 | C | T | Hair or balding pattern: pattern 4 | Neale B |
| rs1432679 | C | T | Systolic blood pressure | Neale B |
| rs1432679 | C | T | Varicose veins of lower extremities | Neale B |
| rs13153019 | C | T | Hematocrit | Astle W |
| rs13153019 | C | T | Red blood cell count | Astle W |
| rs13153019 | C | T | Allergic disease asthma hay fever or eczema | Ferreira M |
| rs9379084 | A | G | Height | GIANT |
| rs9379084 | A | G | Height | GIANT |
| rs9379084 | A | G | Height | GIANT |
| rs9379084 | A | G | Arm fat-free mass left | Neale B |
| rs9379084 | A | G | Arm fat-free mass right | Neale B |
| rs9379084 | A | G | Arm predicted mass left | Neale B |
| rs9379084 | A | G | Arm predicted mass right | Neale B |
| rs9379084 | A | G | Basal metabolic rate | Neale B |
| rs9379084 | A | G | Birth weight of first child | Neale B |
| rs9379084 | A | G | Comparative height size at age 10 | Neale B |
| rs9379084 | A | G | Diabetes diagnosed by doctor | Neale B |
| rs9379084 | A | G | Forced expiratory volume in 1-second, predicted | Neale B |
| rs9379084 | A | G | Forced vital capacity | Neale B |
| rs9379084 | A | G | Forced vital capacity, best measure | Neale B |
| rs9379084 | A | G | Heel bone mineral density | Neale B |
| rs9379084 | A | G | Height | Neale B |
| rs9379084 | A | G | Self-reported diabetes | Neale B |
| rs9379084 | A | G | Sitting height | Neale B |
| rs9379084 | A | G | Trunk fat-free mass | Neale B |
| rs9379084 | A | G | Trunk predicted mass | Neale B |
| rs9379084 | A | G | Whole body fat-free mass | Neale B |
| rs9379084 | A | G | Whole body water mass | Neale B |
| rs4712976 | C | T | Hematocrit | Astle W |
| rs4712976 | C | T | Hemoglobin concentration | Astle W |
| rs4712976 | C | T | Mean corpuscular hemoglobin | Astle W |
| rs4712976 | C | T | Mean corpuscular hemoglobin concentration | Astle W |
| rs4712976 | C | T | Mean corpuscular volume | Astle W |
| rs4712976 | C | T | Red cell distribution width | Astle W |
| rs4712976 | C | T | Reticulocyte count | Astle W |
| rs4712976 | C | T | Reticulocyte fraction of red cells | Astle W |
| rs4712976 | C | T | Serum urate | GUGC |
| rs4712976 | C | T | Serum urate | GUGC |
| rs4712976 | C | T | Arm fat mass left | Neale B |
| rs4712976 | C | T | Arm fat mass right | Neale B |
| rs4712976 | C | T | Arm fat-free mass left | Neale B |
| rs4712976 | C | T | Arm fat-free mass right | Neale B |
| rs4712976 | C | T | Arm predicted mass left | Neale B |
| rs4712976 | C | T | Arm predicted mass right | Neale B |
| rs4712976 | C | T | Basal metabolic rate | Neale B |
| rs4712976 | C | T | Body fat percentage | Neale B |
| rs4712976 | C | T | Body mass index | Neale B |
| rs4712976 | C | T | Disorders of mineral metabolism | Neale B |
| rs4712976 | C | T | Hayfever, allergic rhinitis or eczema | Neale B |
| rs4712976 | C | T | Height | Neale B |
| x | C | T | Hip circumference | Neale B |
| rs4712976 | C | T | Leg fat mass left | Neale B |
| rs4712976 | C | T | Leg fat mass right | Neale B |
| rs4712976 | C | T | Leg fat percentage left | Neale B |
| rs4712976 | C | T | Leg fat percentage right | Neale B |
| rs4712976 | C | T | Leg fat-free mass left | Neale B |
| rs4712976 | C | T | Leg fat-free mass right | Neale B |
| rs4712976 | C | T | Leg predicted mass left | Neale B |
| rs4712976 | C | T | Leg predicted mass right | Neale B |
| rs4712976 | C | T | Mouth or teeth dental problems: dentures | Neale B |
| rs4712976 | C | T | Self-reported hereditary or genetic haematological disorder | Neale B |
| rs4712976 | C | T | Sitting height | Neale B |
| rs4712976 | C | T | Trunk fat mass | Neale B |
| rs4712976 | C | T | Trunk fat percentage | Neale B |
| rs4712976 | C | T | Trunk fat-free mass | Neale B |
| rs4712976 | C | T | Trunk predicted mass | Neale B |
| rs4712976 | C | T | Weight | Neale B |
| rs4712976 | C | T | Whole body fat mass | Neale B |
| rs4712976 | C | T | Whole body fat-free mass | Neale B |
| rs4712976 | C | T | Whole body water mass | Neale B |
| rs4712976 | C | T | Rheumatoid arthritis | Okada Y |
| rs1214759 | A | G | Plateletcrit | Astle W |
| rs1214759 | A | G | Height | Neale B |
| rs1214759 | A | G | Self-reported hypertension | Neale B |
| rs1214759 | A | G | Treatment with blood pressure medication | Neale B |
| rs1214759 | A | G | Vascular or heart problems diagnosed by doctor: high blood pressure | Neale B |
| rs1214759 | A | G | Vascular or heart problems diagnosed by doctor: none of the above | Neale B |
| rs2397112 | A | G | Hemoglobin concentration | Astle W |
| rs2397112 | A | G | Platelet count | Astle W |
| rs2397112 | A | G | Plateletcrit | Astle W |
| rs6486122 | C | T | Plasminogen activator inhibitor 1 PAI 1 concentration | Huang J |
| rs6486122 | C | T | Plasminogen activator inhibitor type 1 levels PAI 1 | Huang J |
| rs6486122 | C | T | Body mass index | Neale B |
| rs6486122 | C | T | Worry too long after embarrassment | Neale B |
| rs6486122 | C | T | Age at menarche | ReproGen |
| rs11031005 | C | T | Menarche age at onset | Pickrell JK |
| rs11031005 | C | T | Sex hormone levels | Ruth KS |
| rs11031005 | C | T | Bilateral oophorectomy | Neale B |
| rs11031005 | C | T | Excessive, frequent and irregular menstruation | Neale B |
| rs11031005 | C | T | Length of menstrual cycle | Neale B |
| rs11031005 | C | T | Years since last cervical smear test | Neale B |
| rs11031005 | C | T | Age at menopause | ReproGen |
| rs3814707 | G | A | High light scatter percentage of red cells | Astle W |
| rs3814707 | G | A | High light scatter reticulocyte count | Astle W |
| rs3814707 | G | A | Immature fraction of reticulocytes | Astle W |
| rs3814707 | G | A | Atopic dermatitis | EAGLE |
| rs3814707 | G | A | Allergic disease | Ferreira M |
| rs3814707 | G | A | Heel bone mineral density | Neale B |
| rs3814707 | G | A | Self-reported eczema or dermatitis | Neale B |
| rs11235688 | A | G | Mean platelet volume | Astle W |
| rs11235688 | A | G | Plateletcrit | Astle W |
| rs11235688 | A | G | Mean platelet volume | Astle W |
| rs11235688 | A | G | Plateletcrit | Astle W |
| rs879619 | A | G | Self-reported hypertension | Neale B |
| rs879619 | A | G | Vascular or heart problems diagnosed by doctor: high blood pressure | Neale B |
| rs8046391 | C | G | Comparative body size at age 10 | Neale B |
| rs8046391 | C | G | Pulse rate | Neale B |
| rs8046391 | C | G | Waist circumference | Neale B |
| rs11078597 | C | T | Serum albumin | Franceschini N |
| rs11078597 | C | T | Serum albumin level | Franceschini N |
| rs11078597 | C | T | Serum albumin level | Franceschini N |
| rs6258 | T | C | Serum sex hormone binding globulin concentrations in men | Ohlsson C |
| rs6258 | T | C | Serum testosterone concentrations in men | Ohlsson C |
| rs6258 | T | C | Sex hormone binding globulin SHBG concentrations | Coviello AD |
| rs6258 | T | C | Untransformed total testosterone in men | Ohlsson C |
| rs6258 | T | C | Sex hormone binding globulin levels | Coviello AD |
| rs6258 | T | C | Testosterone levels | Ohlsson C |
| rs6258 | T | C | Testosterone | Ohlsson C |
| rs727428 | T | C | Circulating sex hormone binding globulin levels | Prescott J |
| rs727428 | T | C | Circulating sex hormone binding globulin levels in non postmenopausal hormone users | Prescott J |
| rs727428 | T | C | Serum dihydrotestosterone DHT level | Jin G |
| rs727428 | T | C | Serum dihydrotestosterone level in dutasteride or placebo treatment group | Jin G |
| rs727428 | T | C | Serum testosterone T level in dutasteride or placebo treatment group | Jin G |
| rs727428 | T | C | Serum testosterone T level in placebo group | Jin G |
| rs727428 | T | C | Androgen levels | Jin G |
| rs727428 | T | C | Androgen levels | Jin G |
| rs727428 | T | C | Sex hormone binding globulin levels | Prescott J |
| rs727428 | T | C | Testosterone | Prins B |
| rs7248104 | A | G | Height | GIANT |
| rs7248104 | A | G | Height | GIANT |
| rs7248104 | A | G | Height | GIANT |
| rs7248104 | A | G | Triglycerides | GLGC |
| rs7248104 | A | G | Triglyceride levels | Spracklen CN |
| rs7248104 | A | G | Triglycerides | GLGC |
| rs7248104 | A | G | Height | Neale B |
| rs7248104 | A | G | Self-reported hypothyroidism or myxoedema | Neale B |
| rs1688043 | T | C | C reactive protein levels or triglyceride levels pleiotropy | Ligthart S |
| rs181255261 | A | G | Cause of death: fatty liver | Neale B |
| rs34954997 | - | TTCG | High light scatter percentage of red cells | Astle W |
| rs34954997 | - | TTCG | High light scatter reticulocyte count | Astle W |
| rs34954997 | - | TTCG | Immature fraction of reticulocytes | Astle W |
| rs34954997 | - | TTCG | Age-related macular degeneration | Fritsche LG |
| rs6020423 | T | C | Granulocyte percentage of myeloid white cells | Astle W |
| rs6020423 | T | C | Monocyte count | Astle W |
| rs6020423 | T | C | Monocyte percentage of white cells | Astle W |
| rs62223042 | A | G | Age at menarche | Neale B |
| rs62223042 | A | G | Body mass index | Neale B |
| rs62223042 | A | G | Relative age of first facial hair | Neale B |
| rs62223042 | A | G | Time spent watching television | Neale B |
| rs738409 | G | C | Hematocrit | Astle W |
| rs738409 | G | C | Hemoglobin concentration | Astle W |
| rs738409 | G | C | Mean corpuscular hemoglobin | Astle W |
| rs738409 | G | C | Platelet count | Astle W |
| rs738409 | G | C | Plateletcrit | Astle W |
| rs738409 | G | C | Red cell distribution width | Astle W |
| rs738409 | G | C | Alanine aminotransferase in plasma | Chambers JC |
| rs738409 | G | C | CT hepatic steatosis | Speliotes EK |
| rs738409 | G | C | Fatty liver | Feitosa |
| rs738409 | G | C | Hepatic steatosis assessed by CT | Chambers JC |
| rs738409 | G | C | Non alcoholic fatty liver disease | Kawaguchi T |
| rs738409 | G | C | Non alcoholic fatty liver disease Matteoni classification Type4 | Kawaguchi T |
| rs738409 | G | C | Nonalcoholic fatty liver disease | Zhang H |
| rs738409 | G | C | Nonalcoholic fatty liver disease | Speliotes EK |
| rs738409 | G | C | Soluble intercellular adhesion molecule 1 ICAM 1 | Pare G |
| rs738409 | G | C | Alanine aminotransferase ALT levels after remission induction therapy in actute lymphoblastic leukemia ALL | Liu Y |
| rs738409 | G | C | Cirrhosis alcohol related | Buch S |
| rs738409 | G | C | Liver enzyme levels alanine transaminase | Chambers JC |
| rs738409 | G | C | Nonalcoholic fatty liver disease | Speliotes EK |
| rs738409 | G | C | Nonalcoholic fatty liver disease | Kawaguchi T |
| rs738409 | G | C | Nonalcoholic fatty liver disease | Chung GE |
| rs738409 | G | C | Alcoholic liver disease | Neale B |
| rs738409 | G | C | Self-reported gout | Neale B |
| rs738409 | G | C | Alanine transaminase | Chambers JC |
| rs738409 | G | C | Non alcoholic fatty liver disease supplementary concept | Speliotes EK |
